# Diagnostic lncRNA biomarkers and immune-related ceRNA networks for osteonecrosis of the femoral head in metabolic syndrome identified by plasma RNA sequencing and machine learning

**DOI:** 10.3389/fimmu.2025.1640657

**Published:** 2025-09-03

**Authors:** Haoyan Sun, Meng Xu, Dianlong Mi, Qingyu Li, Haipeng Sun, Yang Song

**Affiliations:** Orthopedic Medical Center, The Second Affiliated Hospital of Jilin University, Changchun, Jilin, China

**Keywords:** osteonecrosis of the femoral head, lncRNA, diagnostic biomarker, immune infiltration, metabolic syndrome

## Abstract

Osteonecrosis of the femoral head (ONFH) is a disabling orthopedic condition that remains challenging to diagnose at an early stage. Recent evidence suggests that immune dysregulation plays a central role in the development of both ONFH and metabolic syndrome (MetS), a cluster of metabolic abnormalities associated with increased ONFH risk. However, reliable noninvasive diagnostic biomarkers for ONFH, particularly in high-risk MetS populations, are still lacking. This study aimed to identify key diagnostic long non-coding RNAs (lncRNAs) in ONFH patients with MetS and to construct an immune-related competitive endogenous RNA (ceRNA) network. Plasma lncRNA and mRNA expression profiles from 9 ONFH patients and 6 healthy controls were analyzed to identify differentially expressed lncRNAs (DElncRNAs) and mRNAs (DEmRNAs), followed by ceRNA network construction. The MetS dataset from the Gene Expression Omnibus (GEO) was integrated, and weighted gene co-expression network analysis (WGCNA), functional enrichment, protein-protein interaction (PPI) network analysis, MCODE, CytoHubba-MCC, and random forest (RF) algorithms were employed to identify hub mRNAs and their associated lncRNAs. A nomogram model was developed, and diagnostic potential was evaluated using receiver operating characteristic (ROC) analysis and validation in an independent cohort (45 ONFH and 45 control samples). A total of 424 DElncRNAs and 1,431 DEmRNAs were identified, and a ceRNA network involving 7 lncRNAs, 24 miRNAs, and 683 mRNAs was constructed. Integration with the MetS dataset yielded 506 intersecting mRNAs, from which 11 hub mRNAs and 6 related lncRNAs were screened. Five key lncRNAs were selected by RF analysis to construct a diagnostic model with strong predictive performance (AUC > 0.7 in both RNA-seq and qRT-PCR validation). The immune-related ceRNA network also demonstrated significant associations with immune cell infiltration patterns. In conclusion, five candidate lncRNAs (MRPS30-DT, LINC01106, MIR100HG, WDR11-AS1, and PELATON) were identified as promising noninvasive diagnostic biomarkers for ONFH in MetS populations. These findings offer novel insights into immune-related regulatory mechanisms and may support early diagnosis using peripheral blood.

## 1 Introduction

Osteonecrosis of the femoral head (ONFH) is pathologically characterized by localized death of bone cells (osteocytes, osteoblasts, osteoclasts, etc.) and bone marrow, which is primarily caused by disruption of the femoral head’s blood supply (1, 2). As reparative processes fail to restore the necrotic area, structural deterioration and eventual collapse of the femoral head occur (3). According to epidemiological estimates, over 20,000 new ONFH cases are diagnosed annually in the United States, with a total patient population ranging from 300,000 to 600,000 (4). Despite its high disease burden, early ONFH is difficult to diagnose due to its insidious onset, lack of specific symptoms, and limited sensitivity of imaging modalities. Osteoimmunological studies have demonstrated that immune cells regulate the activity of bone marrow mesenchymal stem cells (BMSCs), osteoblasts, and osteoclasts, thereby influencing bone formation and repair (5–9). However, current clinical tools are inadequate for detecting early immune microenvironmental disturbances before structural bone damage becomes apparent. This highlights the urgent need for novel strategies to monitor early immunopathological changes and identify minimally invasive biomarkers for timely ONFH diagnosis (10).

Metabolic syndrome (MetS) is a state of metabolic dysregulation, clinically characterized by obesity, dyslipidemia, hyperglycemia, and hypertension (11). Obesity, dyslipidemia, and hyperglycemia have been shown to increase the risk of ONFH and are considered to be associated with its development (12, 13). Evidence also indicates that immune cells participate in the physiological and pathological processes of MetS and its complications (14, 15).

Recent studies suggest that ONFH and MetS share overlapping molecular mechanisms, including lipid metabolic disorders, dysregulated signaling pathways, and chronic low-grade inflammation. Impaired fatty acid degradation and lipid accumulation are implicated in vascular injury and bone necrosis in ONFH, as well as in insulin resistance and inflammation in MetS (12, 16, 17). The Wnt/β-catenin signaling pathway, which regulates bone formation and metabolic homeostasis, is suppressed in both glucocorticoid-induced ONFH and MetS, suggesting a shared pathogenic mechanism (18–21). Likewise, the Hedgehog signaling pathway regulates hepatic lipid metabolism and osteogenesis, underscoring its dual involvement in ONFH and metabolic disorders (22–24). Although this study does not primarily focus on MetS itself, we chose to investigate ONFH within the MetS population based on their potential associations in terms of metabolic abnormalities, immune mechanisms, and key signaling pathways. Moreover, compared with the general population, MetS patients represent a clinically high-risk group for ONFH and are more likely to undergo routine blood testing, making them well-suited for non-invasive plasma biomarker–based screening strategies. Therefore, identifying ONFH-related immune biomarkers in this population is not only feasible but also of greater clinical relevance.

Increasing evidence suggests that long non-coding RNAs (lncRNAs) play important roles in immune regulation (25, 26), and have been widely explored as potential diagnostic biomarkers in various diseases, such as cancer (27), periodontitis (28), and cardiovascular diseases (29). In the context of ONFH, lncRNAs such as MALAT1 (30, 31), HOTAIR (32), GAS5 (33), EPIC1 (34), NORAD (35), MIAT (36), and DGCR5 (37) have been implicated in dexamethasone-induced cytotoxicity or in the regulation of osteogenic and adipogenic differentiation of BMSCs—both of which are important to ONFH pathogenesis. Moreover, lncRNA expression profiles in necrotic bone tissue and BMSCs from ONFH patients differ significantly from those of fracture patients (38–41), further supporting the diagnostic potential of lncRNAs in early ONFH. Recent studies have shown that several lncRNAs participate in MetS-related processes and may serve as valuable biomarkers (42, 43). For example, APQ AS expression correlates with inflammatory biomarkers, while MALAT1 modulates inflammation and oxidative stress by targeting NF-κB and Keap1–Nrf2 pathways, and is positively associated with pro-inflammatory cytokines such as IL-6 and TNF-α (44–46). HOTAIR and GAS5 have been implicated in metabolic dysregulation, particularly in processes such as insulin resistance, adipose tissue inflammation, and lipid metabolism abnormalities (47). These findings suggest that certain lncRNAs may have overlapping relevance to both ONFH and MetS. In particular, MALAT1, HOTAIR, and GAS5 have been studied in the context of both conditions, indicating their potential as molecular links. However, most ONFH-related lncRNA studies have focused on bone tissue or BMSCs, with limited analyses using peripheral blood and few comparisons to healthy controls. Given their accessibility and noninvasive nature, plasma lncRNAs hold promise as biomarkers for early ONFH detection, especially in high-risk populations such as those with MetS.

Despite the potential of lncRNAs in ONFH diagnosis, functional annotation remains challenging due to their non-coding nature and lack of well-defined ontology. In contrast, mRNAs are better characterized. The competitive endogenous RNA (ceRNA) hypothesis provides an indirect approach to infer lncRNA function by constructing regulatory networks based on shared microRNA (miRNA) interactions (48). In this model, lncRNAs competitively bind miRNAs, relieving their inhibition on target mRNAs and thus influencing downstream gene expression (48). Recent studies have highlighted the relevance of ceRNA networks in bone-related diseases. For example, MALAT1 promotes osteoclast differentiation by sponging miR-329-5p and upregulating PRIP (49), and FGD5-AS1 enhances STAT3 expression via miR-296-5p to reduce steroid-induced apoptosis in ONFH cells (50). HOTAIR has also been shown to regulate osteogenic differentiation in non-traumatic ONFH by targeting miR-17-5p (51). Additionally, immune cell-associated gene signatures have been identified in steroid-induced ONFH, suggesting that immune infiltration plays a key role in its progression (52). Given the contribution of immune dysregulation to ONFH, especially in the context of MetS, constructing an immune-associated lncRNA–miRNA–mRNA ceRNA network may help uncover novel regulatory pathways and diagnostic biomarkers.

In this study, we aimed to identify plasma lncRNA biomarkers for the early diagnosis of ONFH, particularly in individuals with MetS, a high-risk population. To this end, we first performed high-throughput RNA sequencing to identify differentially expressed lncRNAs (DElncRNAs) and mRNAs (DEmRNAs) in plasma samples from ONFH patients and healthy controls. A lncRNA–miRNA–mRNA ceRNA network was then constructed to explore potential regulatory interactions. To further enhance disease specificity and functional relevance, we incorporated the GSE98895 dataset to identify MetS-related mRNA co-expression modules via weighted gene co-expression network analysis (WGCNA). MRNAs shared between the ONFH ceRNA network and MetS modules were defined as ONFH–MetS–related targets, from which upstream regulatory lncRNAs were extracted. Finally, machine learning algorithms were employed to select robust lncRNA biomarkers with diagnostic potential. This integrated approach may provide new insights into ONFH pathogenesis and facilitate the development of noninvasive plasma-based screening strategies.

## 2 Materials and methods

### 2.1 Clinical sample collection

**Figure 1:**
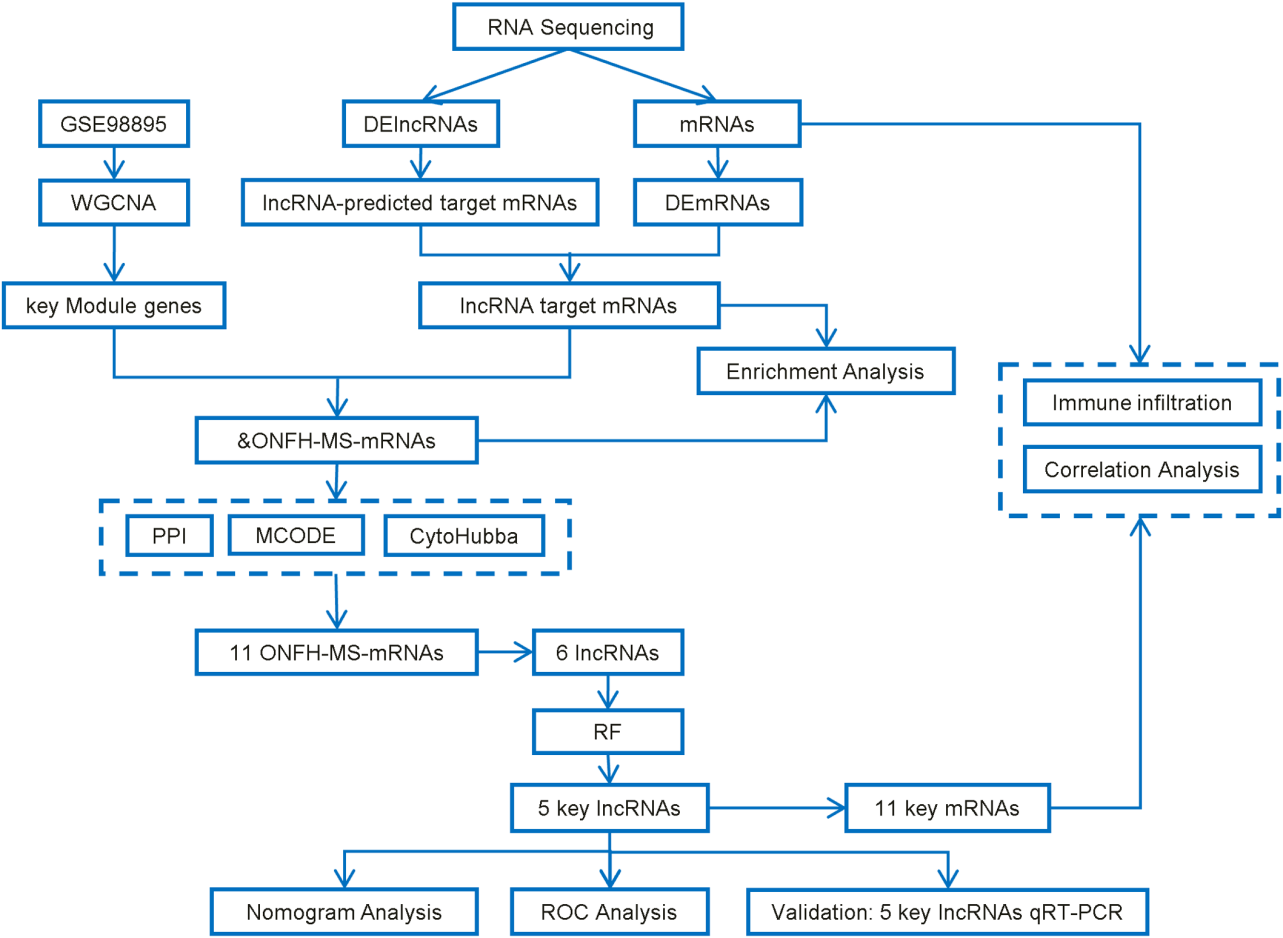
Workflow of this study.

**Figure 1** illustrates the study’s workflow. This study was approved by the Ethics Committee of Second Affiliated Hospital of Jilin University (Ethical Approval No.: 2023-207). Between February and November 2023, nine patients diagnosed with non-traumatic ONFH who underwent hip replacement at the Department of Joint Surgery, The Second Affiliated Hospital of Jilin University, were enrolled in the ONFH group, alongside six healthy controls from routine physical exams. The diagnosis of ONFH was based on imaging, clinical history, and Ficat staging. Patients with ONFH secondary to trauma, tumors, autoimmune diseases, congenital hip anomalies, or genetic disorders were excluded from the study. The controls had no history of ONFH, hip disorders, malignancies, immune diseases, heavy alcohol consumption, or prolonged corticosteroid use. Fasting peripheral blood samples (5 mL) were collected between 6:00 and 7:00 AM on the second day after admission. Plasma was isolated and stored at −80 °C.

### 2.2 RNA extraction and RNA sequencing

Total RNA was extracted from plasma using TRIzol™ reagent (Invitrogen, Thermo Fisher Scientific, China) according to the manufacturer’s protocol. rRNA was removed using the MGIEasy rRNA Depletion Kit (MGI Tech Co., Ltd., China) to enrich lncRNAs and mRNAs. The remaining RNA was fragmented and reverse-transcribed into double-stranded cDNA using the MGIEasy RNA Directional Library Preparation Kit (MGI Tech Co., Ltd., China). The double-stranded cDNA was adenylated and ligated with adapters, followed by amplifying the ligated product and circularizing the PCR product to generate a single-stranded circular DNA library. Sequencing was performed on the DNBSEQ platform (MGI Tech Co., Ltd., China).

This RNA extraction was performed on a total of 15 plasma samples (9 ONFH patients and 6 healthy controls), which were used exclusively for RNA sequencing to identify differentially expressed lncRNAs.

### 2.3 Quality control and differential expression analysis of lncRNAs and mRNAs

The raw sequencing data were strictly filtered using SOAPnuke (v1.5.2). Clean reads were aligned to the human reference genome using HISAT (v2.0.4), and Bowtie2 was further employed to align the clean reads to the reference genome, generating alignment results. Transcriptome sequencing was conducted on human plasma samples using the NCBI reference genome Homo_sapiens (GCF_000001405.39_GRCh38.p13). In the quantitative transcript expression analysis, transcript randomness, coverage, and sequencing saturation were evaluated to ensure the biological and statistical validity of the data. Pearson correlation coefficients of gene expression levels between samples were calculated to assess sample correlations. Principal component analysis (PCA) was applied to gene expression data for dimensionality reduction, identifying outliers and closely related sample clusters. Box plots of gene expression were generated for each sample to assess data dispersion and gene expression density plots were created to identify the primary distribution range of gene expression. Genes were classified based on expression levels (TPM ≤ 1, TPM: 1-10, TPM ≥ 10), and TPM values were log^2^-transformed before downstream analysis to stabilize variance. Their distribution across samples was visualized using stacked bar charts. Differentially expressed lncRNAs and mRNAs between the ONFH and control groups were identified using DESeq2, with thresholds of |log^2^ (fold change) | ≥ 1 and Q-value ≤ 0.05. Volcano plots and heatmaps were generated using the R (Version 4.4.2) packages ggplot2 and pheatmap.

### 2.4 Construction of the lncRNA ceRNA network and enrichment analysis of mRNAs in the network

Seven lncRNAs were selected for ceRNA network analysis based on the following criteria: (1) |log^2^(fold change)| ≥ 1 and adjusted p-value < 0.05; (2) consistent and detectable expression across ONFH plasma samples; (3) annotation in both starBase v2.0 (53) (https://rnasysu.com/encori/index.php) and LncBase v3 databases (54) (https://diana.e-ce.uth.gr/lncbasev3/interactions); and (4) reliable primer design feasibility for Quantitative real-time Polymerase Chain Reaction (qRT-PCR).

MiRNAs predicted to interact with the selected lncRNAs were identified using both starBase v2.0 and LncBase v3, and only the overlapping miRNAs from the two databases were retained. Subsequently, miRNA-targeted mRNAs were predicted using the MiRWalk database (http://mirwalk.umm.uni-heidelberg.de/), and intersected with the DEmRNAs identified from ONFH plasma samples to generate the final target mRNA set. The ceRNA regulatory network was then constructed based on these filtered lncRNA–miRNA and miRNA–mRNA interaction pairs.

Gene Ontology (GO) and Kyoto Encyclopedia of Genes and Genomes (KEGG) enrichment analyses were performed on the final mRNAs using the Metascape database (55) (https://metascape.org). GO terms and KEGG pathways with p-values less than 0.05 were considered statistically significant, and the enrichment results were visualized using the ggplot2 R package.

### 2.5 Acquisition of the MetS dataset (GSE98895) and WGCNA analysis

The raw dataset GSE98895 (56), which contains samples from 20 normal individuals and 20 patients with MetS, was downloaded from the Gene Expression Omnibus (GEO) database (57) (https://www.ncbi.nlm.nih.gov/geo/). Weighted Gene Co-expression Network Analysis (WGCNA) (58) was executed to identify modules associated with MetS. A weighted adjacency matrix was constructed using a power function, and the optimal soft threshold (β) was determined using the “pickSoftThreshold” function. The adjacency matrix was then transformed into a Topological Overlap Matrix (TOM). The network connectivity of each gene was defined as the sum of its adjacency relationships with all other genes, and dissimilarity (1-TOM) was computed. Hierarchical clustering was applied to group genes with similar expression profiles into gene modules, with a minimum module size of n = 30. A gene dendrogram was constructed, and module eigengene dissimilarities were calculated. Finally, the feature gene network was visualized.

### 2.6 ONFH-MetS-mRNAs enrichment analysis

The mRNAs from the ONFH lncRNA ceRNA network were intersected with genes from the key modules in the MetS dataset to identify ONFH-MetS-mRNAs, which were subsequently subjected to GO and KEGG enrichment analysis using the method described in Section 2.4.

### 2.7 Identification of key mRNAs and lncRNAs

A protein-protein interaction (PPI) network was constructed using the STRING database (59) (www.string-db.org) with a minimum interaction score threshold of 0.400 and subsequently visualized using Cytoscape (60). Key nodes in the network were identified using the CytoHubba-MCC and MCODE plugins, which were applied to select the intersection of key ONFH-MetS mRNAs. The lncRNA ceRNA network for these mRNAs was then identified based on the constructed ceRNA network. To further prioritize candidate lncRNAs, Random Forest (RF) analysis (61) was performed using the “randomForest” R package (62) (version 4.7.1.2), with the number of trees (ntree) set to 500. A fixed random seed was applied before model training using set.seed(123) to ensure reproducibility. Variable importance was evaluated using the Mean Decrease in Gini index.

### 2.8 The construction and evaluation of key lncRNAs risk prediction model

A nomogram was developed to provide clinical value for the diagnosis of ONFH. Based on the selected candidate lncRNAs, the nomogram was constructed using the “rms” R package. “Points” represent the score for each candidate lncRNA, and “Total Points” refers to the sum of the scores of all selected lncRNAs. We used the ‘pROC’ R package to evaluate the accuracy of our model by plotting the receiver operating characteristic (ROC) curve and calculating the area under the curve (AUC), with an AUC > 0.7 considered an ideal diagnostic threshold.

To mitigate overfitting and assess generalizability, we further implemented 3-fold cross-validation using the “caret” package. Cross-validated predictions were pooled to construct an overall ROC curve, which was used to evaluate model discrimination. These predictions were also used to generate Decision Curve Analysis (DCA) curves to assess clinical utility. Finally, to simulate real-world deployment, a final logistic regression model was retrained on the full dataset, and the corresponding nomogram and calibration curve were plotted. Calibration was performed using bootstrap resampling (B = 1000), demonstrating strong agreement between predicted and observed risks.

### 2.9 Validation of key lncRNAs risk prediction model by qRT-PCR

To validate the reliability of the results, RNA was extracted from plasma samples of 45 ONFH patients and 45 controls using the Starvio cfRNA Easy Kit (Shanghai Starvio Biotechnology Co., Ltd., China). Reverse transcription was conducted using the SureScript™ First-Strand cDNA Synthesis Kit (Cat. No. QP056, GeneCopoeia, China). qRT-PCR was used according to the manufacturer’s instructions using BlazeTaq™ SYBR^®^ Green qPCR Mix 2.0 (Cat. No. QP031, GeneCopoeia, China). The results were analyzed using the 2^−ΔΔCt^ method, with ACTB as the internal reference gene for normalizing lncRNA expression data. Five candidate lncRNAs (MRPS30-DT, LINC01106, MIR100HG, PELATON, and WDR11-AS1) were selected for validation. Primer sequences used for each lncRNA are provided in **Table 1** at the end of the manuscript. ROC curves for each lncRNA were plotted, and AUC values were calculated to construct the diagnostic model.

**Table 1:** Primers designed for qRT-PCR validation of lncRNAs in the diagnostic model.

LncRNA name	Forward and reverse primer
MRPS30_DT	F:GTGGGGATCTGGAGTGGAAG
	R:TGGGTTGCAAAAAGCCCCTT
LINC01106	F:GTGGGGATCTGGAGTGGAAG
	R:TGGGTTGCAAAAAGCCCCTT
MIR100HG	F:TCGAACTTTGGAGTGTGGCA
	R:GGCACAAAGCTCCCTGGTTA
PELATON	F:CCTGAGGACTGTGTGTTCCC
	R:CCTCAGCAGCCAACAGGTTA
WDR11_AS1	F:TGTGGTGCCCAAGAGCTATG
	R:ATGGCTCAAGTGTCAGAGGC
ACTB	F:GTGGCCGAGGACTTTGATTG
	R:CCTGTAACAACGCATCTCATATT

This validation step involved an independent cohort of 90 plasma samples (45 ONFH patients and 45 healthy controls) and aimed to confirm the differential expression of key lncRNAs revealed by RNA sequencing.

### 2.10 Immune infiltration analysis

Immune cell infiltration analysis was conducted using the “CIBERSORT” R package (63) to estimate the relative abundance of 22 lymphocyte subtypes in both normal and ONFH samples. The LM22 signature matrix was used as a reference, with 100 permutations (perm = 100). A fixed random seed (set.seed(123)) was applied prior to model execution to ensure reproducibility. Cell types with zero abundance across all samples were removed from the results. To evaluate differences in immune cell composition between groups, Wilcoxon rank-sum tests were performed. Additionally, Spearman correlation analysis was executed between the expression levels of lncRNA-regulated key mRNAs and immune cell proportions, using ONFH samples only.

### 2.11 Statistical analysis

Data processing and analyses were performed using SPSS version 26.0 and R software (Version 4.4.2). Comparisons between samples were carried out using t-tests or chi-square tests. Patient baseline characteristics are presented as the mean ± standard deviation, and a p-value < 0.05 was considered statistically significant. Spearman correlation analysis was applied to examine the relationship between mRNAs regulated by key lncRNAs and immune cells. A p-value < 0.05 was considered statistically significant.

## 3 Results

### 3.1 Patient basic information and quality control

The average age of patients in the ONFH group was 58.67 ± 9.46 years, whereas the average age of healthy participants in the control group was 63.33 ± 9.22 years (**Supplementary Material, Table 1**). Statistical analysis revealed no significant differences between the two groups in terms of age (P > 0.05). The results of data quality control were carefully evaluated to ensure the reliability of downstream transcriptomic analysis and are provided in the supplementary material (**Supplementary Material, Figure 1**-**11**, **Tables 2**-**4**).

### 3.2 Identification of DElncRNAs and DEmRNAs

**Figure 2:**
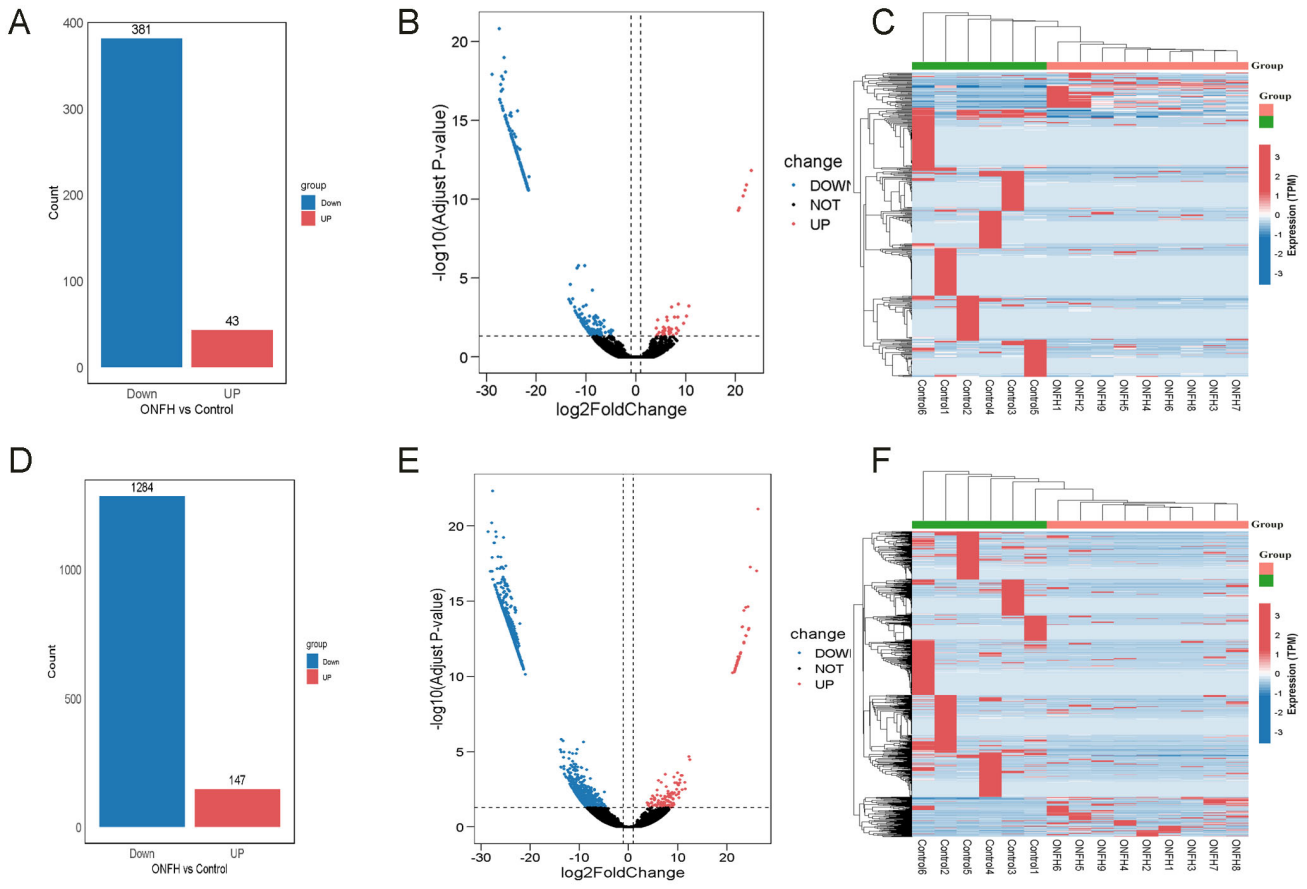
The bar plots, volcano plots, and heatmaps of DElncRNAs and DEmRNAs. **(A)** Bar plot of the number of DElncRNAs. **(B)** Volcano plot of DElncRNAs. **(C)** Clustering heatmap of DElncRNAs. **(D)** Bar plot of the number of DEmRNAs. **(E)** Volcano plot of DEmRNAs. **(F)** Clustering heatmap of DEmRNAs.

**Figure 3:**
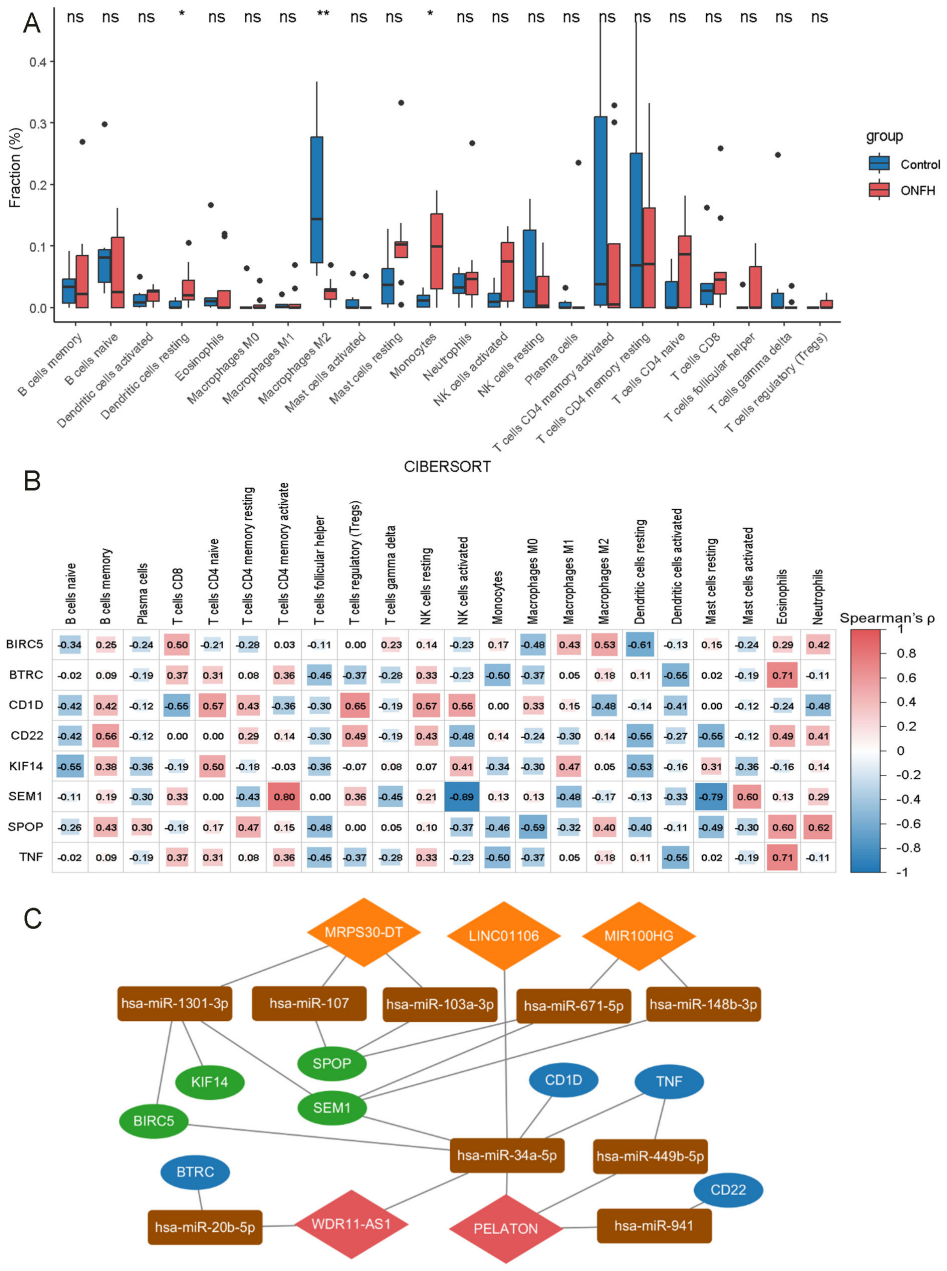
Immune cell infiltration analysis in ONFH. **(A)** Comparative analysis of 22 immune cell types between ONFH and control groups (ns: no significance, *p < 0.05, **p < 0.01). **(B)** Correlation analysis of 8 mRNAs with various immune cell types. **(C)** Core lncRNA immune-related ceRNA network. Orange diamonds represent upregulated lncRNAs, red diamonds indicate downregulated lncRNAs, rectangles represent miRNAs, green ovals correspond to upregulated mRNAs, and blue ovals represent downregulated mRNAs.

In the ONFH patient group, 424 DElncRNAs (43 upregulated and 381 downregulated) (**Figures 2A–C**) and 1431 DEmRNAs (147 upregulated and 1284 downregulated) (**Figures 2D–F**) were identified, compared to the control group. These results indicate a substantial transcriptomic alteration associated with ONFH. Notably, among the differentially expressed mRNAs, several immune-related genes, including SPOP, TNF, CD22, CD1D, and others, were known to play key roles in immune cell activation and inflammatory regulation. Furthermore, ceRNA network analysis revealed that these immune genes are regulated by lncRNAs including MRPS30-DT, LINC01106, MIR100HG, PELATON, and WDR11-AS1 (**Figure 3C**). Detailed differential expression statistics of these lncRNA–mRNA pairs are provided in **Supplementary Material, Table 5**.

### 3.3 Construction of the lncRNA ceRNA network and enrichment analysis of mRNAs in the network

**Figure 4:**
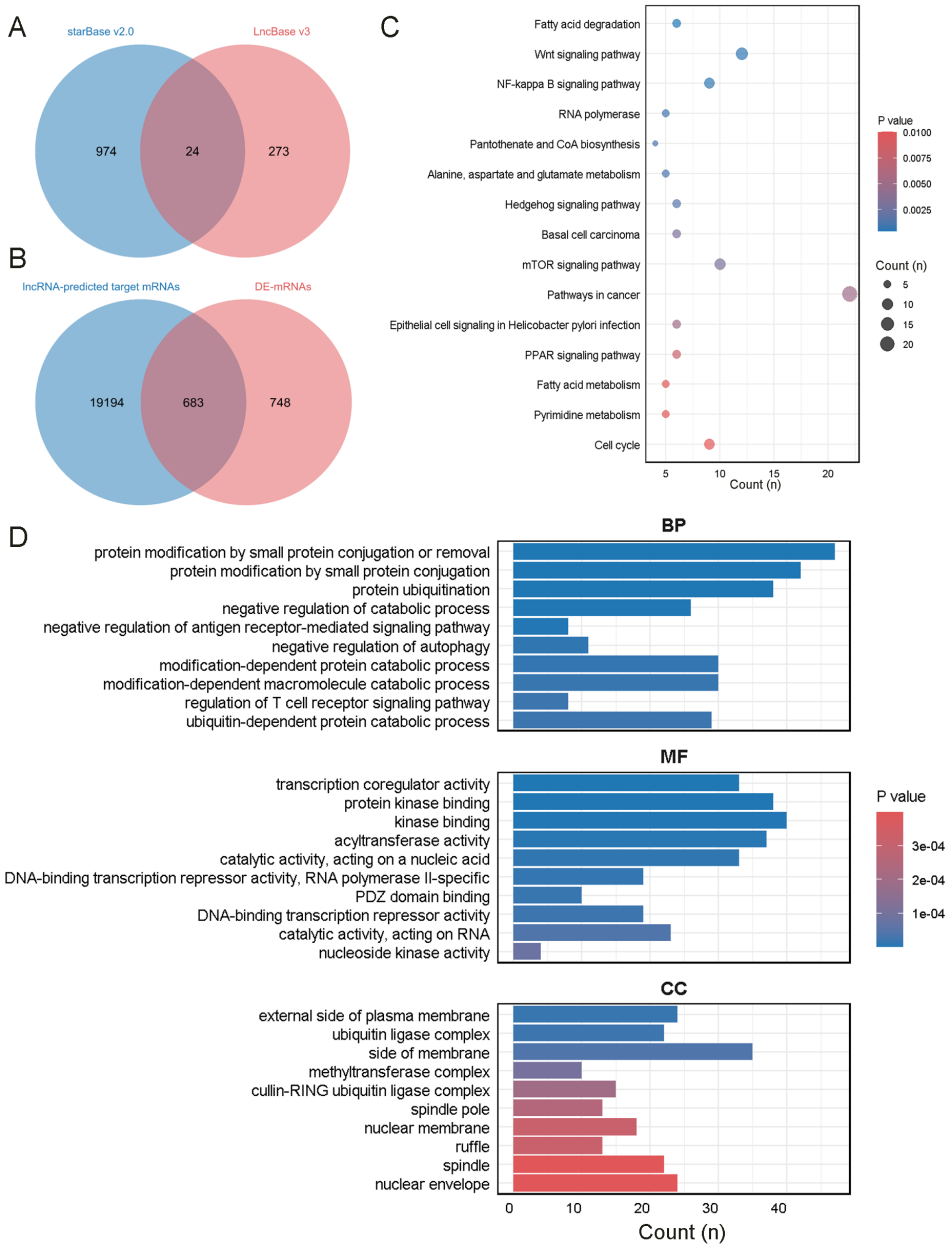
Enrichment analysis of mRNAs in the network. **(A)** The intersection of miRNAs predicted by starBase v2.0 and LncBase v3. **(B)** The intersection of mRNAs predicted by miRWalk and DEmRNAs in the plasma of ONFH patients. **(C)** KEGG analysis of the mRNAs. **(D)** GO analysis of the mRNAs.

Seven lncRNAs were selected for further analysis based on their FC value, Q-value, expression levels in the samples, and inclusion in starBase v2.0 and LncBase v3. Annotation details for the lncRNAs are summarized in **Table 2**. These lncRNAs overlapped with 24 miRNAs predicted by starBase v2.0 and LncBase v3 (**Figure 4A**). 19,877 mRNAs were predicted as targets of the 24 miRNAs using miRWalk. By intersecting the lncRNA-predicted target mRNAs with 1,431 DEmRNAs from the plasma of ONFH patients, 683 target mRNAs were identified (**Figure 4B**). A ceRNA regulatory network was constructed, consisting of 7 lncRNAs, 24 miRNAs, and 683 mRNAs (**Supplementary Material, Table 6**). This network reflects the potential post-transcriptional regulatory landscape mediated by lncRNAs in ONFH. KEGG analysis further revealed significant enrichment in fatty acid degradation, Wnt signaling pathways, NF-kappa B signaling pathways, Hedgehog signaling pathways, mTOR signaling pathways, PPAR signaling pathways, and fatty acid metabolism (**Figure 4C**). These pathways are associated with inflammation, lipid metabolism, and cell proliferation, suggesting their involvement in ONFH pathogenesis. GO analysis of these 683 mRNAs revealed significant enrichment in the biological process of protein modification by small protein conjugation or removal. The most enriched molecular functions included transcription coregulator activity, protein kinase binding, and kinase binding. The cellular component analysis revealed that the most enriched category was the external side of the plasma membrane (**Figure 4D**).

**Table 2:** Annotation information of lncRNAs.

Gene symbol	Regulation	Chromosome	Map loc	Start	End	Strand	log2FoldChange	Q-value
LINC00630	UP	NC_000023.11	Xq22.1	10276913	10264323	+	22.18125013	1.56048E-12
MRPS30-DT	UP	NC_000005.10	5p12	4474328	4408793	-	6.527571158	0.024635353
LINC01106	UP	NC_000002.12	2q13	110375109	110384536	-	6.631867245	0.035579441
FAM201A	UP	NC_000009.12	9p13.1	38621088	38623384	+	5.450417187	0.0124588
MIR100HG	UP	NC_000011.10	11q24.1	12202839	12242871	-	7.103866475	6.66E-04
PELATON	DOWN	NC_000020.11	20q13.13	50267478	50279788	+	-6.954937628	0.039044083
WDR11-AS1	DOWN	NC_000011.10	11q26.12	120761812	120851179	-	-6.123165599	0.027309225

### 3.4 WGCNA analysis and identification of key gene modules in the MetS dataset (GSE98895)

**Figure 5:**
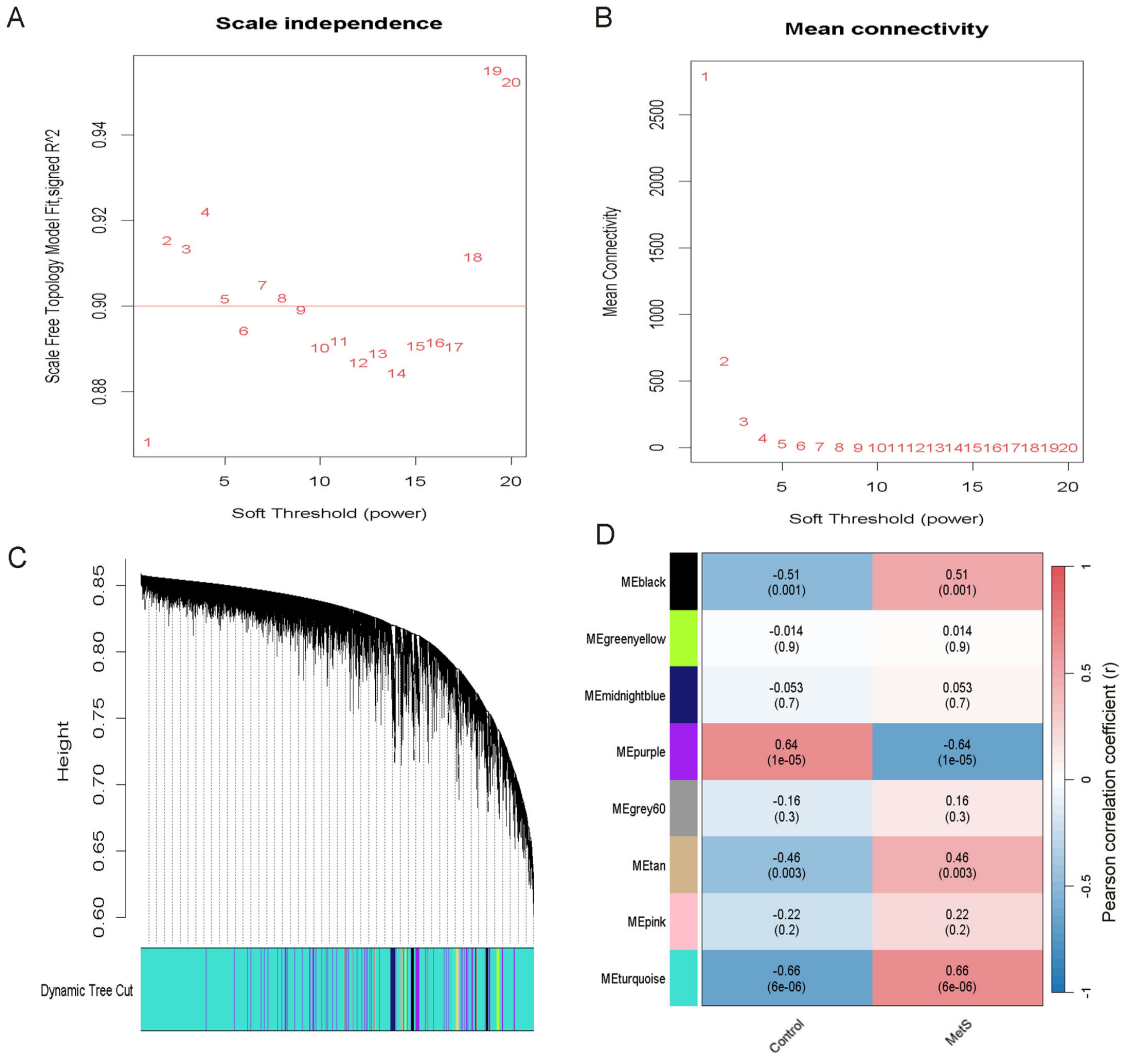
Identification of the most relevant module genes in MetS through WGCNA. **(A)** Scale-free Topology Fit Index Plot, selecting β = 1 as the optimal soft threshold. **(B)** Mean Connectivity Plot. **(C)** Gene co-expression modules in MetS. **(D)** Heatmap showing the association between modules and MetS. The turquoise module shows a strong positive correlation with MetS. The correlation coefficients and p-values are represented by the numbers and the numbers in parentheses, respectively.

WGCNA was performed to identify gene modules significantly associated with MetS. A scale-free network was constructed using a soft threshold of β = 1, achieving a scale independence of R² = 0.9 (**Figures 5A, B**). A hierarchical clustering tree was generated, followed by dynamic tree cutting (**Figure 5C**), which resulted in eight distinct gene modules visualized as a heatmap (**Figure 5D**). The MEturquoise module (cor = 0.66, P = 6 × 10^-6^) and the MEpurple module (cor = −0.64, P = 1 × 10^-5^) showed the strongest positive and negative correlations with MetS, respectively. The MEturquoise module comprised 15,840 genes, indicating a potentially critical gene set for metabolic regulation.

### 3.5 ONFH-MetS-mRNAs enrichment analysis

**Figure 6:**
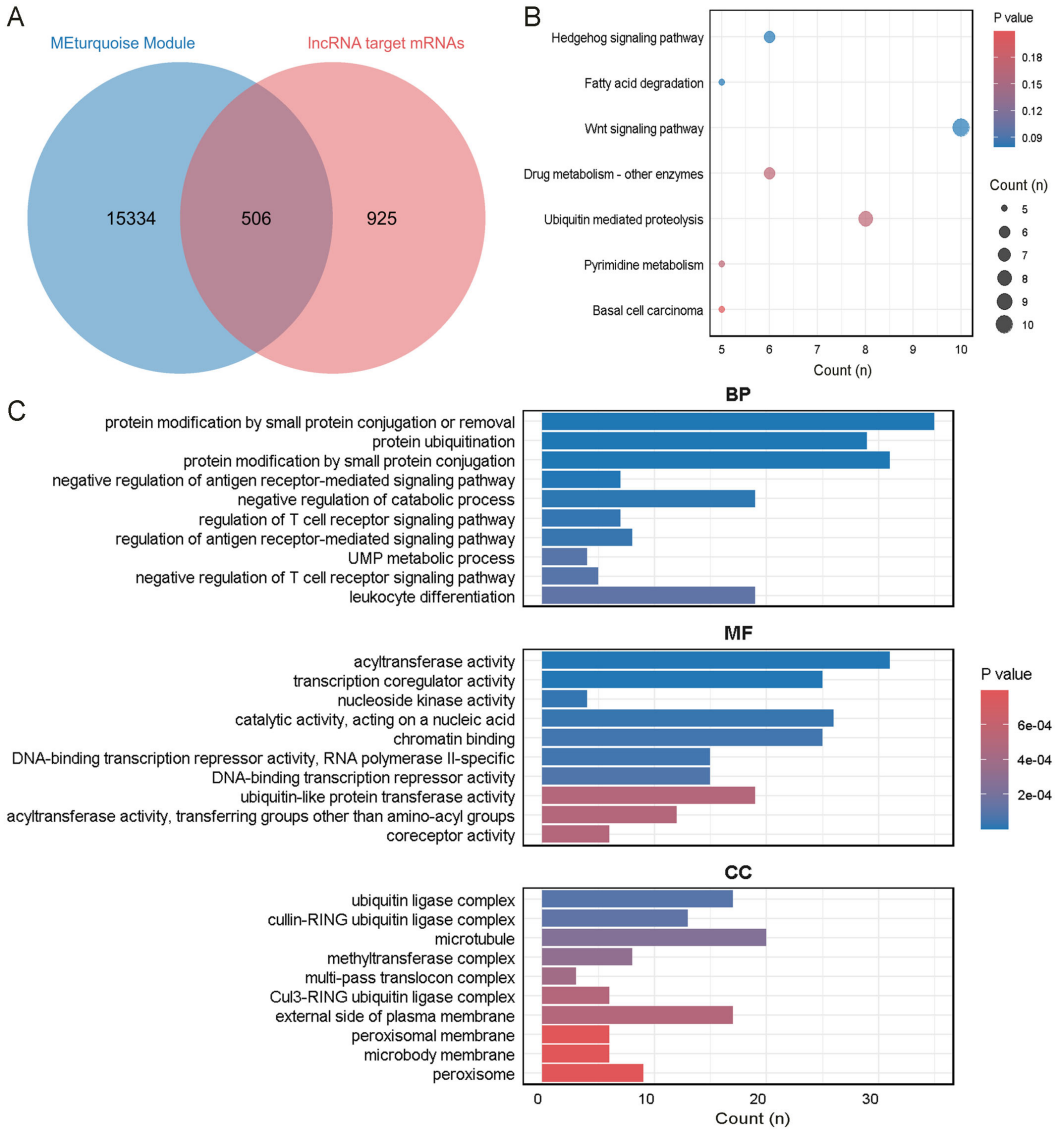
Enrichment analysis of the intersecting genes between ONFH and MetS. **(A)** Intersection of mRNAs in the ONFH lncRNA ceRNA network and genes in the MEturquoise module. **(B)** KEGG analysis of the mRNAs. **(C)** GO analysis of the mRNAs.

A total of 506 overlapping mRNAs were identified by intersecting the mRNAs from the lncRNA ceRNA network with genes in the MetS-associated MEturquoise module (**Figure 6A**). KEGG pathway analysis indicated significant enrichment in the Hedgehog signaling pathway, fatty acid degradation, and Wnt signaling pathway (**Figure 6B**). These pathways are known to be involved in tissue development, lipid metabolism, and inflammatory signaling, and may contribute toxONFH pathogenesis through metabolic–immune crosstalk. GOxenrichment analysis revealed that the most significantly enriched biological process was protein modification by small protein conjugation or removal. The top molecular functions included acyltransferase activity and transcription coregulator activity. The most enriched cellular components included the ubiquitin ligase complex, cullin-RING ubiquitin ligase complex, and microtubule (**Figure 6C**).

### 3.6 Identification of key mRNAs and lncRNAs

**Figure 7:**
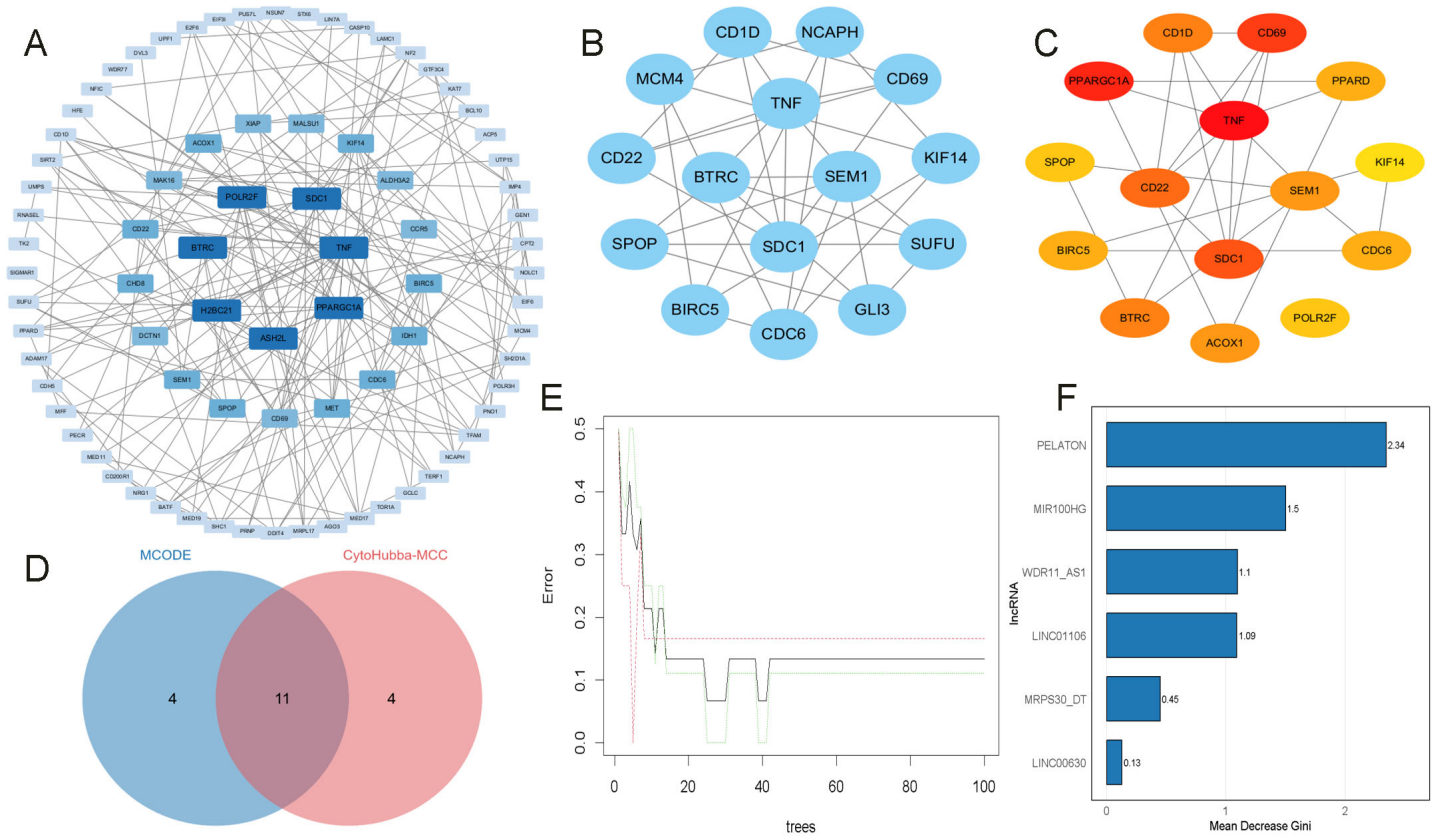
Identification of key mRNAs and lncRNAs. **(A)** PPI analysis of OMFH-MetS-mRNAs, displaying only nodes with a degree ≥ 5. **(B)** Selection of the key gene cluster with 15 genes using MCODE. **(C)** Top 15 hub genes identified by CytoHubba-MCC. **(D)** Intersection of mRNAs from MCODE and CytoHubba-MCC plugins. **(E, F)** RF screening of key DMPs, ranked by their importance scores.

A PPI network analysis was first conducted on 506 ONFH-MetS-related mRNAs (**Supplementary Material, Table 7**), and hub genes with a degree ≥ 5 were visualized using Cytoscape v3.10.3 (**Figure 7A**). A key cluster of 15 mRNAs was further identified using MCODE (**Figure 7B**). The top 15 hub mRNAs were selected using the CytoHubba-MCC plugin (**Figure 7C**), and their intersection yielded 11 candidate mRNAs (**Figure 7D**), including BIRC5, KIF14, SEM1, SPOP, BTRC, CD1D, CD69, CDC6, TNF, CD22, and SDC1. These 11 mRNAs were regulated by six lncRNAs in the ceRNA network, namely LINC00630, MRPS30-DT, LINC01106, MIR100HG, PELATON, and WDR11-AS1. To construct a more robust diagnostic model, Random Forest was used to select the top five most important lncRNAs (**Figures 7E, F**), MRPS30-DT, LINC01106, MIR100HG, PELATON, and WDR11-AS1.

### 3.7 The construction and evaluation of key lncRNAs risk prediction model

**Figure 8:**
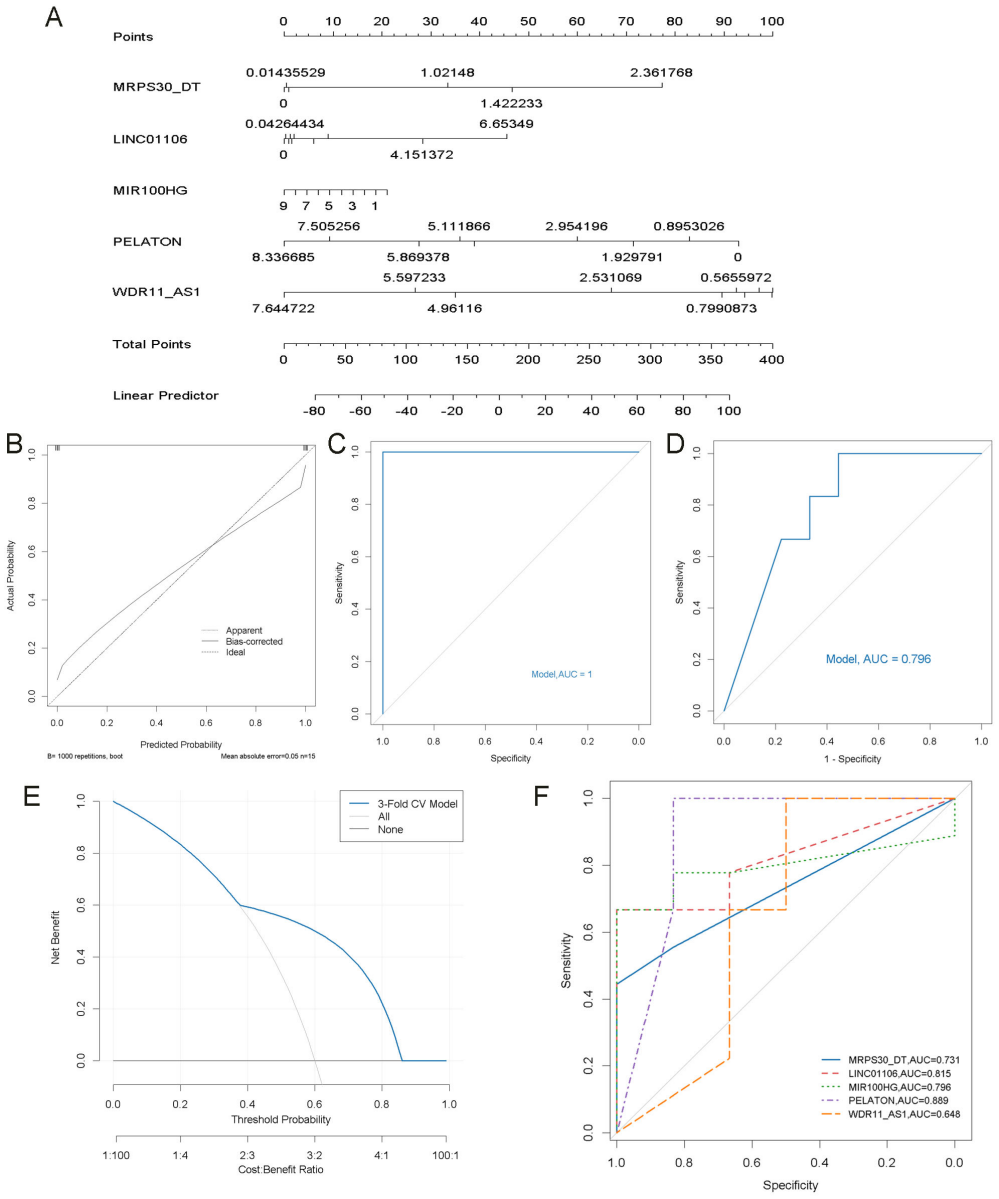
Construction of the nomogram model and its ROC curve. **(A)** Construction of a nomogram model based on 5 key lncRNAs to predict the risk in OA patients. **(B)** Calibration curve to assess the prediction accuracy of the nomogram model. **(C)** ROC curve analysis of the nomogram model. **(D)** ROC curve of the nomogram model based on 3-fold cross-validated predictions. **(E)** DCA based on cross-validated predictions. **(F)** ROC curve analysis of each key lncRNA.

A nomogram incorporating five key lncRNAs was constructed (**Figure 8A**). Calibration curve analysis demonstrated minimal discrepancies between the predicted and observed risks (**Figure 8B**). The diagnostic performance of the model and each individual lncRNA was assessed by ROC analysis. The combined model demonstrated excellent discriminatory power, achieving an AUC of 1.00 on the training data (**Figure 8C**). To reduce overfitting and assess generalization, 3-fold cross-validation was performed, and the pooled cross-validated predictions were used to draw an overall ROC curve, yielding an AUC of 0.796 (**Figure 8D**). DCA analysis based on the same predictions showed a net clinical benefit over default strategies in the 0.4–0.8 threshold range (**Figure 8E**). The AUC values for the five lncRNAs were as follows: MRPS30_DT = 0.731, LINC01106 = 0.815, MIR100HG = 0.796, PELATON = 0.889, and WDR11_AS1 = 0.648 (**Figure 8F**). Although the combined model’s AUC was lower than that of some individual lncRNAs, this is likely due to the use of cross-validation, which provides a more realistic and conservative estimate. These findings support the robustness and clinical relevance of the multivariable model compared with individual biomarkers.

### 3.8 Validation of key lncRNAs risk prediction mode

**Figure 9:**
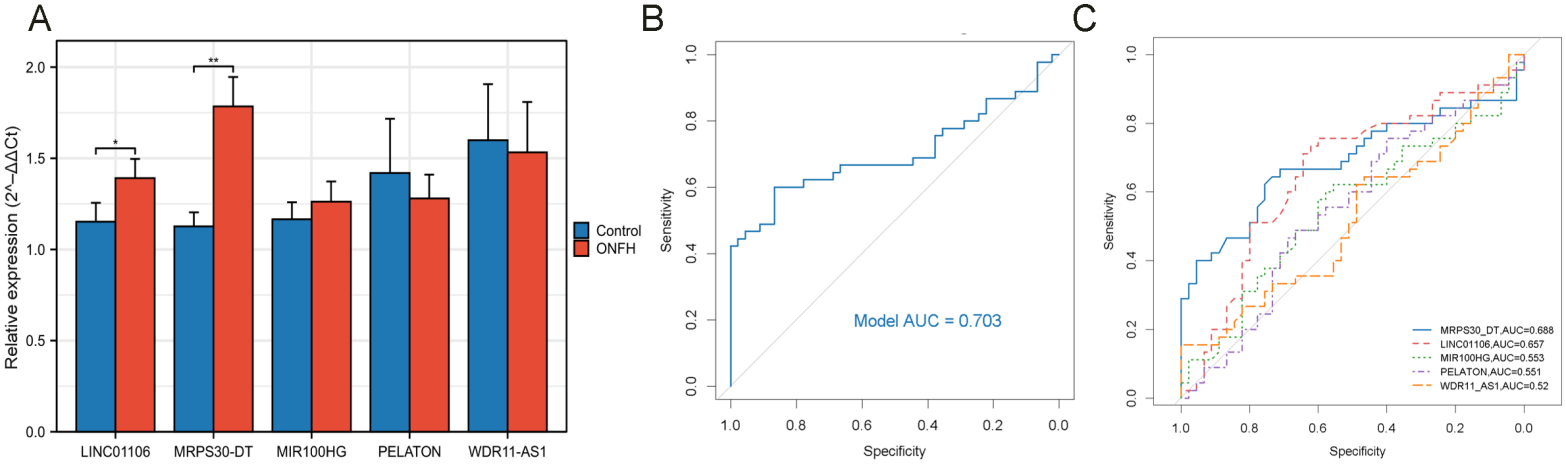
qRT-PCR validation of key lncRNAs. **(A)** Relative expression of five lncRNAs detected by qRT-PCR (ns: no significance, *p < 0.05, **p < 0.01). **(B)** ROC curve analysis of five lncRNAs. **(C)** ROC curve analysis of each key lncRNA.

To further validate the accuracy of the integrated bioinformatics analysis, qRT-PCR was conducted to measure the expression levels of five candidate lncRNA diagnostic biomarkers in plasma samples from 45 patients with ONFH and 45 healthy controls. The results revealed that MRPS30-DT and LINC01106 were significantly upregulated in patients with ONFH, while MIR100HG exhibited an increasing trend. In comparison, PELATON and WDR11-AS1 show a downward trend (**Figure 9A**). These expression trends are consistent with those observed in the RNA sequencing data, confirming the robustness of the findings. The combined lncRNA model achieved an AUC greater than 0.7 (**Figure 9B**), surpassing the AUCs of individual lncRNAs (**Figure 9C**), indicating the potential clinical utility and translational value of this diagnostic model.

### 3.9 Immune infiltration analysis

The CIBERSORT algorithm was employed to estimate the relative proportions of 22 immune cell types in each sample. Compared to the control group, the ONFH group exhibited elevated proportions of resting dendritic cells and monocytes, and a decreased proportion of M2 macrophages, indicating a substantial alteration in the immune microenvironment (**Figure 3A**). Further analysis revealed associations between the 11 hub mRNAs and immune cell infiltration. Specifically, BIRC5, BTRC, CD1D, CD22, KIF14, SEM1, SPOP, and TNF were correlated with various immune cell types, suggesting potential roles in immune regulation (**Figure 3B**). A ceRNA subnetwork comprising five lncRNAs and eight immune-related mRNAs was constructed (**Figure 3C**), highlighting possible lncRNA-mediated regulation of immune signaling pathways in ONFH.

## 4 Discussion

ONFH is a debilitating orthopedic condition that markedly impairs patients’ quality of life (64). Owing to poorly understood molecular mechanisms and a lack of reliable biomarkers, early diagnosis and targeted therapy for ONFH remain challenging (65). Although several lncRNAs, such as MALAT1 (66) and AWPPH (67), have been implicated in the regulation of ONFH, comprehensive analyses of peripheral blood lncRNA profiles in ONFH patients versus healthy controls remain limited.

MetS, which has been increasingly associated with ONFH, involves immune dysregulation and disordered lipid metabolism—both of which are also important features of ONFH—suggesting a potential molecular link between the two conditions. However, existing studies have largely overlooked the association between ONFH and MetS, limiting the diagnostic potential of lncRNAs in metabolic contexts. Notably, several lncRNAs, including MALAT1, HOTAIR, and GAS5, exhibit dysregulated expression in both ONFH and MetS, suggesting that they may serve as molecular bridges linking the two diseases. Our functional enrichment analysis revealed that the 506 mRNAs overlapping between the lncRNA ceRNA network and MetS-related WGCNA modules were significantly enriched in metabolism-related pathways, including fatty acid degradation, Wnt signaling, and Hedgehog signaling, indicating potential shared regulatory mechanisms between ONFH and MetS. Impaired fatty acid degradation may contribute to lipid accumulation (16, 17), while disturbances in Wnt and Hedgehog signaling could affect bone formation (18, 24). Additionally, altered protein degradation pathways suggest potential disruption of cellular homeostasis, leading to chronic inflammation and tissue damage (68, 69). These findings provide a theoretical basis and new perspective for early ONFH screening in the context of metabolic dysfunction.

In this study, we performed high-throughput sequencing of peripheral blood lncRNAs and mRNAs from ONFH patients and healthy controls. By integrating bioinformatics and machine learning approaches, we identified five key lncRNAs (MRPS30_DT, LINC01106, MIR100HG, PELATON, and WDR11_AS1) and their immune-related ceRNA networks, and constructed a predictive nomogram specific to ONFH in patients with MetS. Given that all samples in this study and the MetS dataset from the GEO database were derived from peripheral blood, assessing the expression levels of these five lncRNAs in MetS patients represents a feasible strategy for ONFH risk prediction. Peripheral blood testing is widely used in the diagnosis of various diseases (70, 71). Although these lncRNAs have shown potential as independent diagnostic biomarkers, we aim to further refine the model by developing a quantitative scoring system based on their expression levels (72). Higher scores would indicate greater predictive value, thus enabling early monitoring and intervention in MetS patients—critical for timely diagnosis and management of ONFH.

The five lncRNAs identified in this study as potentially associated with ONFH have not been previously reported in the context of this disease. LINC01106 is upregulated in lung adenocarcinoma, non-small cell lung cancer, and bladder cancer (73–75) and functions as a novel diagnostic and prognostic marker in colorectal and gastric adenocarcinomas (76, 77). LINC01106 regulates mRNA expression by acting as a sponge for hsa-miR-34a-5p (78). lncRNA Tmem235 modulates BIRC5 expression by competitively binding to miR-34a-3p (79). Our results suggest that LINC01106 upregulation enhances the expression of BIRC5 and SEM1 by sequestering hsa-miR-34a-5p. In ONFH samples, BIRC5 and SEM1 expression is closely correlated with immune cell infiltration, suggesting a potential role in modulating the immune microenvironment of ONFH.

MIR100HG is implicated in a variety of human diseases. It is highly expressed in the blood of patients with herniated discs and in several cancers, including colorectal, gastric, and osteosarcoma (80–83). MIR100HG promotes the proliferation of nasopharyngeal carcinoma cells via the miR-136-5p/IL-6 axis (84) and enhances the growth of triple-negative breast cancer cells through the miR-5590-3p/OTX1 axis (85). TGFβ induces MIR100HG expression, amplifying TGFβ signaling by promoting the expression and secretion of TGFβ1 (86). TGFβ also promotes Th17 cell differentiation (87), with elevated levels of Th17 cells and IL-17 observed in the peripheral blood of ONFH patients (88). Th17 cells secrete IL-9 (89), which is elevated in ONFH cartilage and contributes to cartilage degradation via activation of the JAK-STAT signaling pathway *in vitro* (90). MRPS30-DT is significantly upregulated in breast cancer (91), and its co-expressed genes are enriched in pathways related to Th17 cell differentiation (92). Collectively, MIR100HG and MRPS30-DT may play pivotal roles in ONFH pathogenesis.

WDR11-AS1 expression is downregulated in osteoarthritic cartilage and inhibits inflammation-induced extracellular matrix (ECM) degradation by directly binding to PABPC1, highlighting its potential as a therapeutic target for osteoarthritis (93). WDR11-AS1 is positively co-expressed with TNF (94). In our ceRNA network, downregulation of WDR11-AS1 reduces its sequestration of hsa-miR-34a-5p, thereby diminishing TNF expression. TNF activates the NF-κB signaling pathway, which regulates the production of pro-inflammatory cytokines and the recruitment of inflammatory cells, thereby promoting inflammation (95). Therefore, we hypothesize that WDR11-AS1 may be critical in ONFH pathogenesis.

PELATON is upregulated in the tissues and plasma of patients with inflammatory bowel disease and gastric cancer (96, 97) and may serve as a potential biomarker for assessing the incidence and prognosis of acute coronary syndrome (ACS) (98). PELATON functions as an inhibitor of ferroptosis. Knockdown of PELATON enhances reactive oxygen species (ROS) production and induces ferroptosis (99). Ferroptosis is critically involved in the pathogenesis of steroid-induced ONFH (SONFH), while SIRT6 suppresses ferroptosis, mitigates vascular endothelial damage, promotes osteogenic differentiation, and prevents femoral head necrosis (100). Inhibiting ferroptosis may protect bone cells from oxidative damage, enhance bone repair in necrotic regions, and improve skeletal outcomes in SONFH patients, offering a promising strategy for disease intervention (101–103). Accordingly, reduced PELATON expression may promote ferroptosis and contribute to ONFH development. Thus, PELATON may serve as a diagnostic biomarker and a therapeutic target for ONFH.

Growing evidence indicates that inflammatory osteoimmunology plays a critical role in the pathogenesis of ONFH (104–107). ONFH is a chronic inflammatory disorder in which persistent inflammation within and around the lesions disrupts the dynamic balance between bone formation and resorption, enhancing osteoclastic activity, suppressing osteogenesis, and ultimately accelerating femoral head collapse (108–111). Studies have demonstrated that a macrophage-mediated chronic inflammatory immune microenvironment plays a pivotal role in ONFH progression. During the progression of ONFH, macrophages infiltrating necrotic bone tissue predominantly undergo polarization toward the pro-inflammatory M1 phenotype, leading to a disrupted M1/M2 macrophage ratio and elevated secretion of pro-inflammatory cytokines, including IL-1β, TNF-α, and IL-6. This immunological shift contributes to local immune dysregulation, perpetuates chronic inflammatory responses, and ultimately impairs bone tissue regeneration (18, 112). In addition to macrophages, neutrophils, T cells, and B cells are also implicated in ONFH pathogenesis. Activated neutrophils release neutrophil extracellular traps (NETs), which are web-like structures composed of chromatin and antimicrobial proteins. In ONFH patients, NET formation within small blood vessels surrounding the femoral head disrupts local microcirculation, contributing to ischemia (113). Imbalances in T cell subsets, B cell populations, and cytokine expression have been observed in ONFH tissues (107, 110, 114). Consistent with our findings, ONFH patients exhibit increased proportions of resting dendritic cells and monocytes, along with reduced levels of M2 macrophages. Elucidating the inflammatory signaling pathways and immune cell interactions underlying ONFH is essential for advancing diagnostic and therapeutic strategies.

Our study has several limitations. First, although high-throughput sequencing of lncRNAs and mRNAs was performed on samples from nine ONFH patients and six healthy controls, the overall sample size remains relatively limited, which may affect the generalizability of the findings. Second, the findings from our bioinformatics analyses—including the ceRNA network, the CIBERSORT-based immune cell differences between ONFH patients and healthy controls, and the correlations between hub genes and immune cell subsets—were all derived from computational predictions and have not been experimentally validated. Therefore, these findings require further investigation through *in vitro* and *in vivo* experiments. Finally, ONFH and MetS are multifactorial conditions with complex biological interactions. As this study focused on a select number of plasma-derived transcripts, it may not reflect the full range of molecular mechanisms involved.

## 5 Conclusions

High-throughput sequencing was performed to profile lncRNA and mRNA expression in plasma samples from 9 ONFH patients and six healthy controls. Through integrated bioinformatics and machine learning approaches, five ONFH-associated lncRNAs (MRPS30-DT, LINC01106, MIR100HG, WDR11-AS1, and PELATON) were systematically identified, and a diagnostic nomogram specific to ONFH in MetS patients was established. Moreover, immune dysregulation was observed in ONFH patients with MetS, and an immune-related lncRNA ceRNA network was constructed. This study identifies peripheral blood lncRNAs with diagnostic potential for ONFH in MetS patients and highlights novel molecular pathways and targets for future therapeutic strategies and precision medicine.

## Data Availability

In this study, the lncRNA and mRNA sequencing data of the ONFH and control groups are publicly available. These data can be found at: 10.5281/zenodo.16890593.
