# Supplementary material for: Diagnostic lncRNA biomarkers and immune-related ceRNA networks for osteonecrosis of the femoral head in metabolic syndrome identified by plasma RNA sequencing and machine learning
Source: Front Immunol. 2025 Sep 3;16:1640657. doi: 10.3389/fimmu.2025.1640657 (PMC12440747; doi:10.3389/fimmu.2025.1640657)
Supplement: Supplementary file 1 [file DataSheet1.docx]

**Supplementary Figures and Tables**

**1. Supplementary Figures**


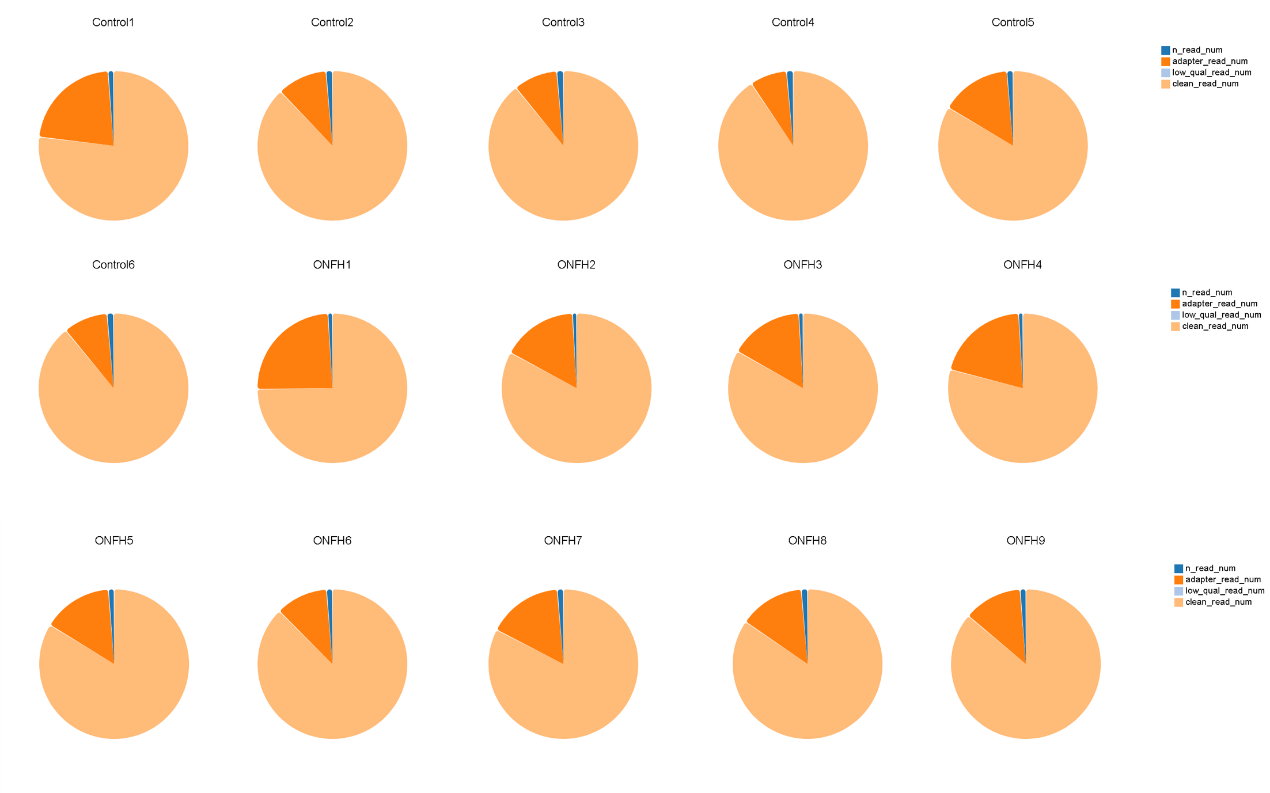


**Supplementary Figure 1.** Raw reads filter component statistics. N, proportion of reads with a high content of unknown base “N”; Adapter, proportion of reads contaminated with sequencing adapters; Low quality, proportion of low-quality reads; Clean reads, proportion of reads retained after quality filtering.


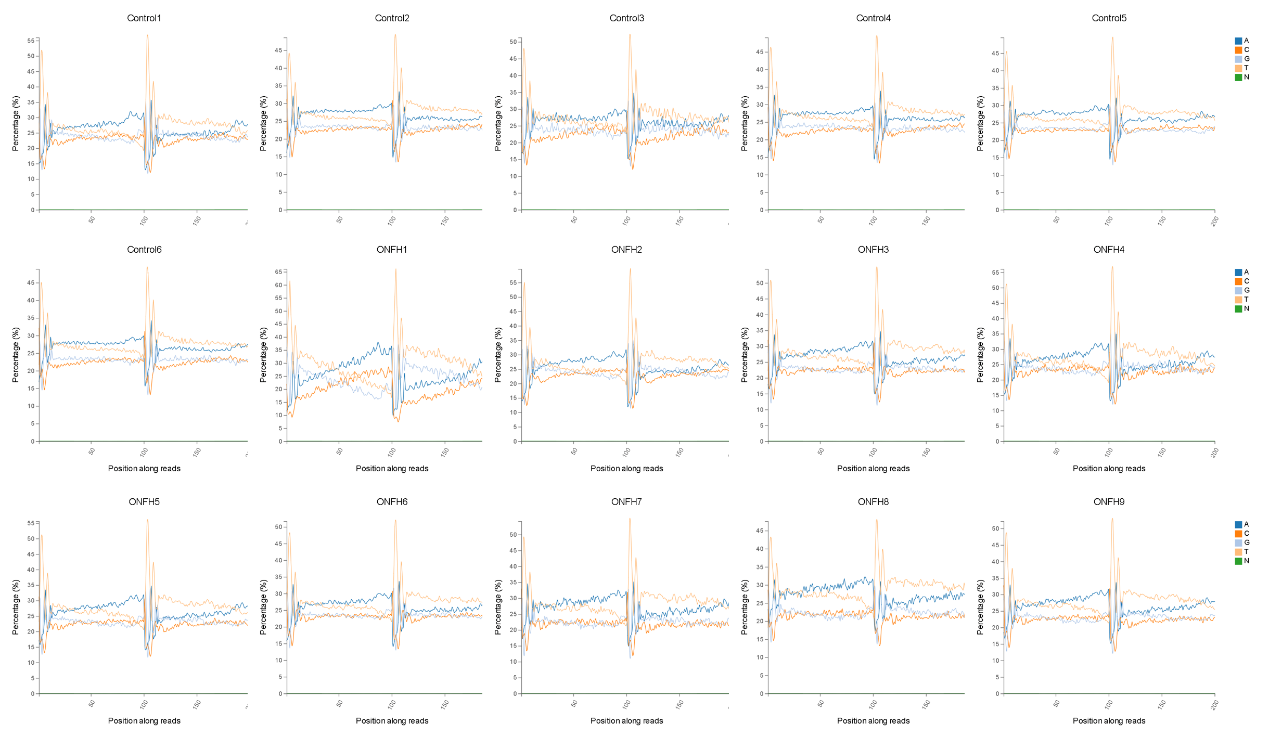


**Supplementary Figure 2.** Clean reads base content distribution. X-axis, position of bases within the reads; Y-axis, proportion of each type of base at the corresponding position.


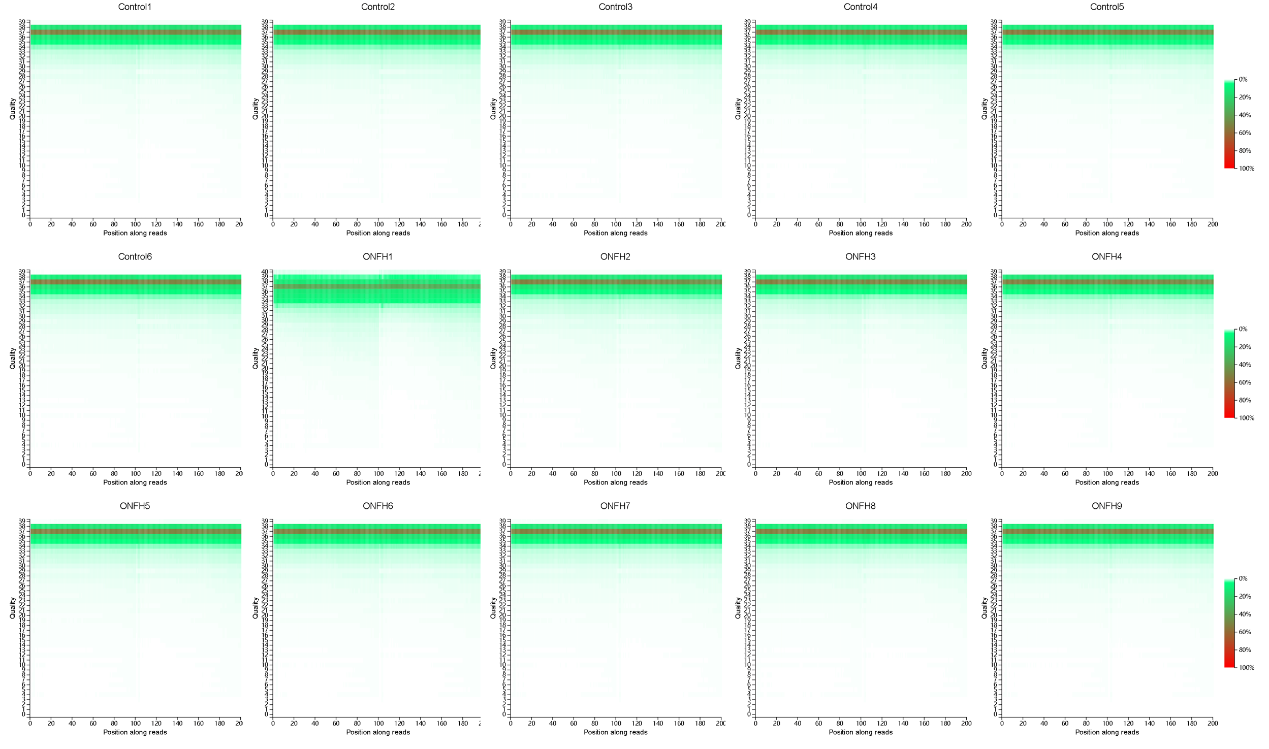


**Supplementary Figure 3.** Clean reads base mass distribution. X-axis, position of bases within the reads; Y-axis, base quality score.


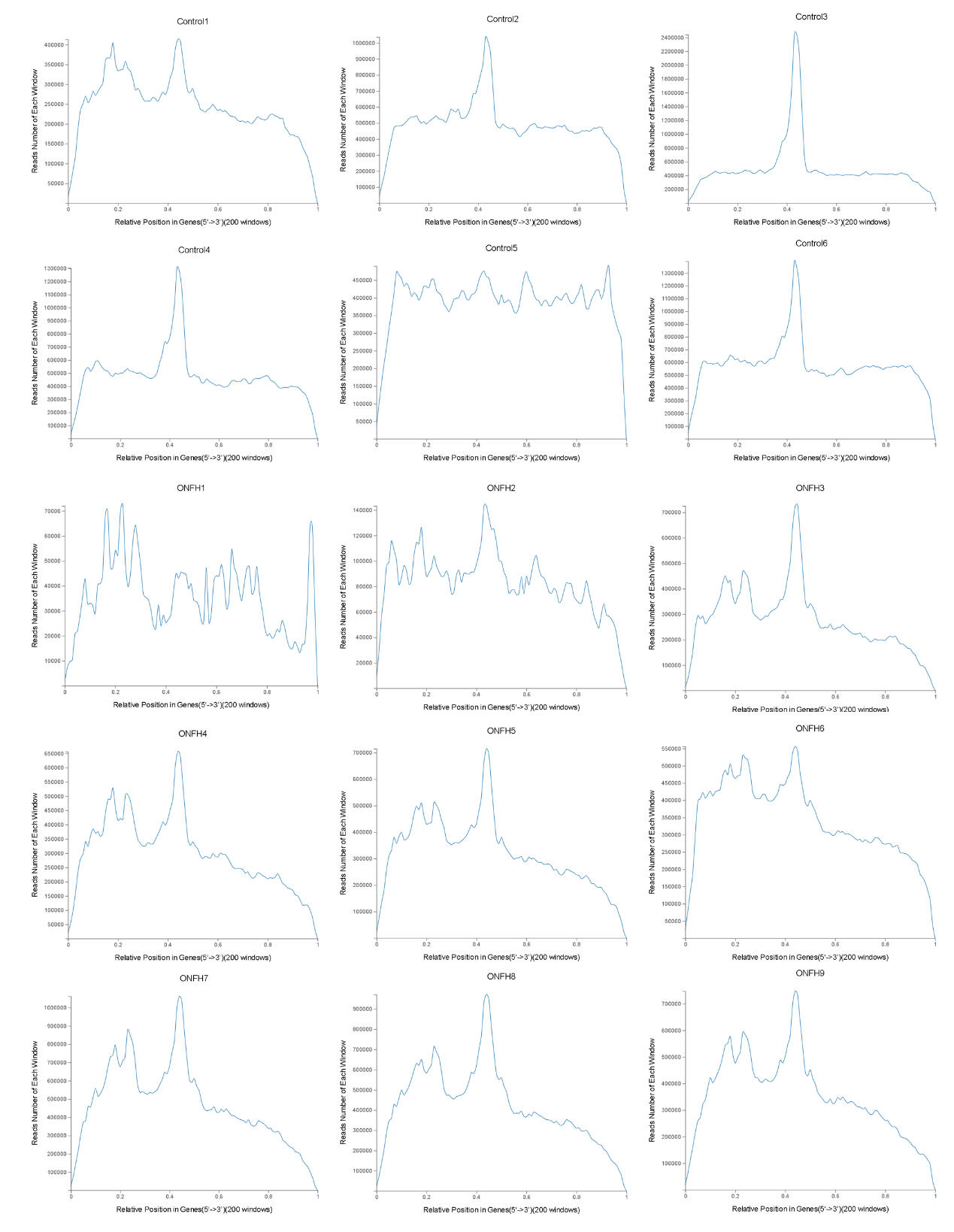


**Supplementary Figure 4.** Distribution of reads on transcripts. X-axis, transcript position (200 sliding windows); Y-axis, number of reads calculated within each sliding window.


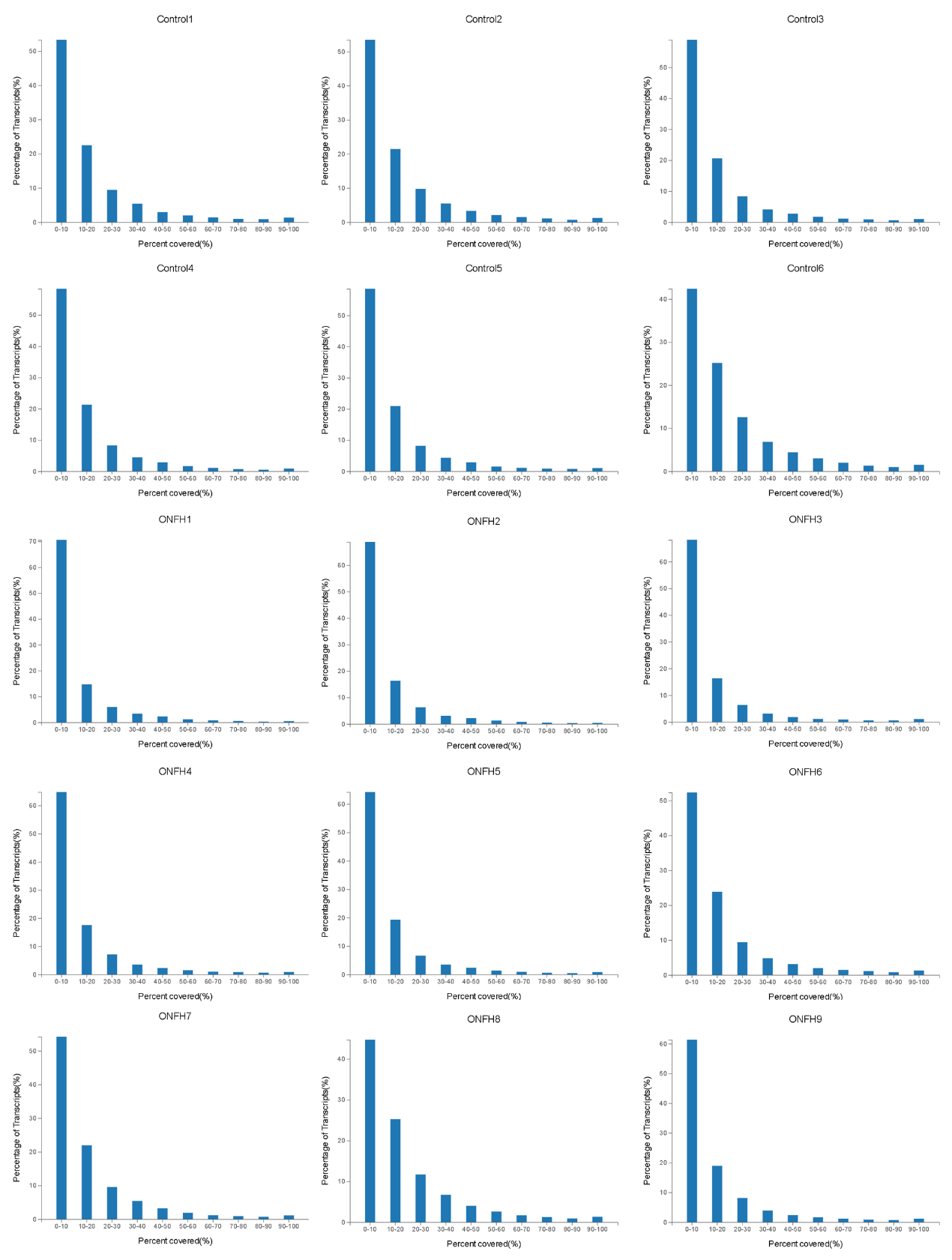


**Supplementary Figure 5.** Reads coverage of transcripts. X-axis, coverage of transcripts by reads; Y-axis, proportion of transcripts.


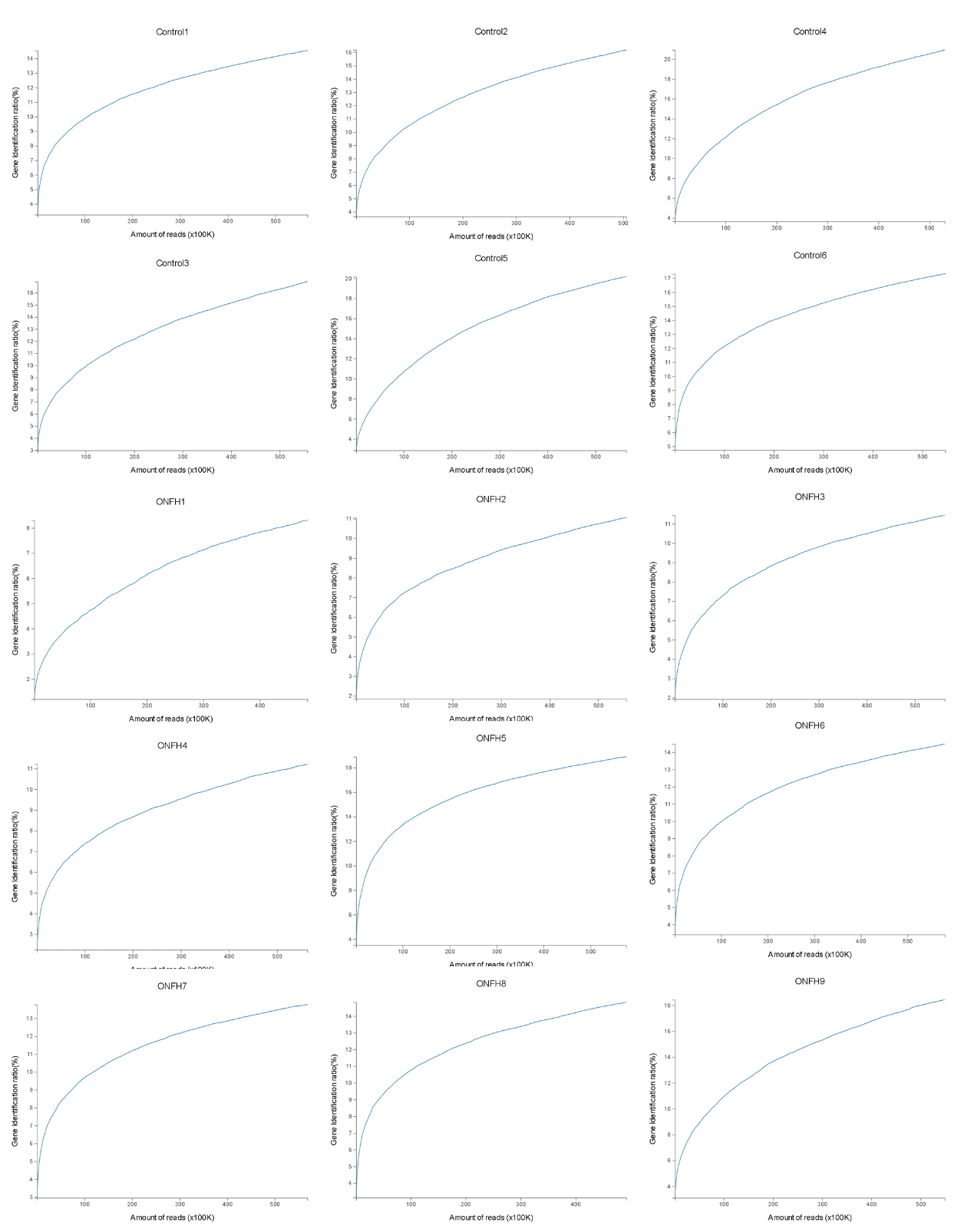


**Supplementary Figure 6.** Sequencing saturation curve.


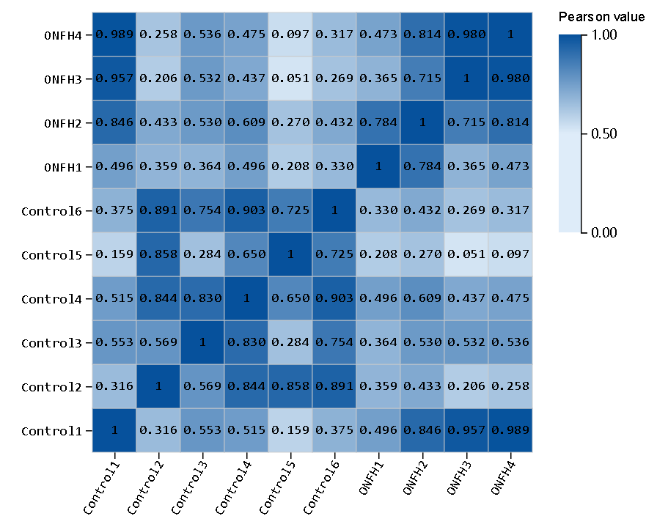


**Supplementary Figure 7.** Sample correlation heat map.


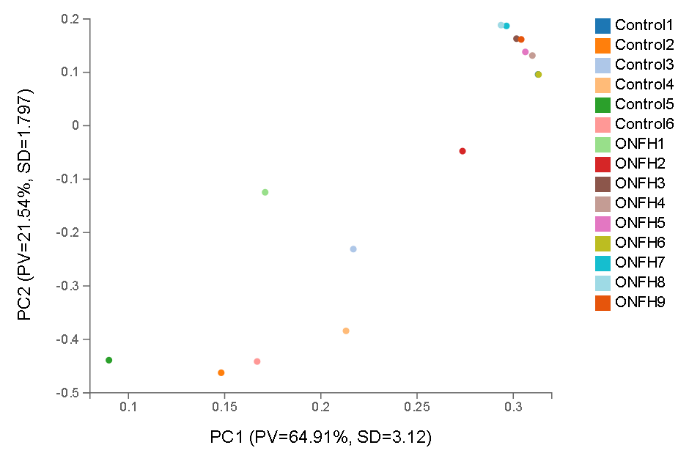


**Supplementary Figure 8.** principal component analysis (PCA).


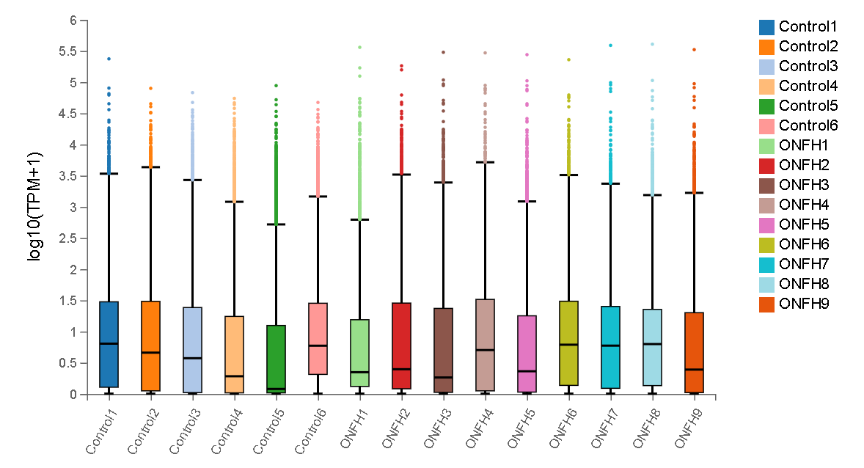


**Supplementary Figure 9.** Expression box plot.


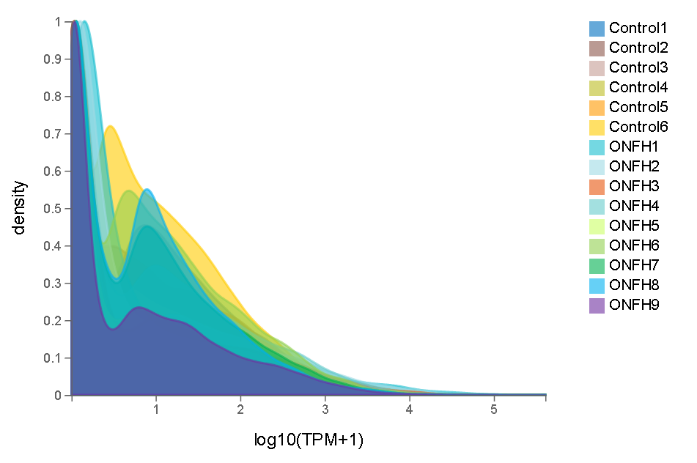


**Supplementary Figure 10.** Expression Density Chart.


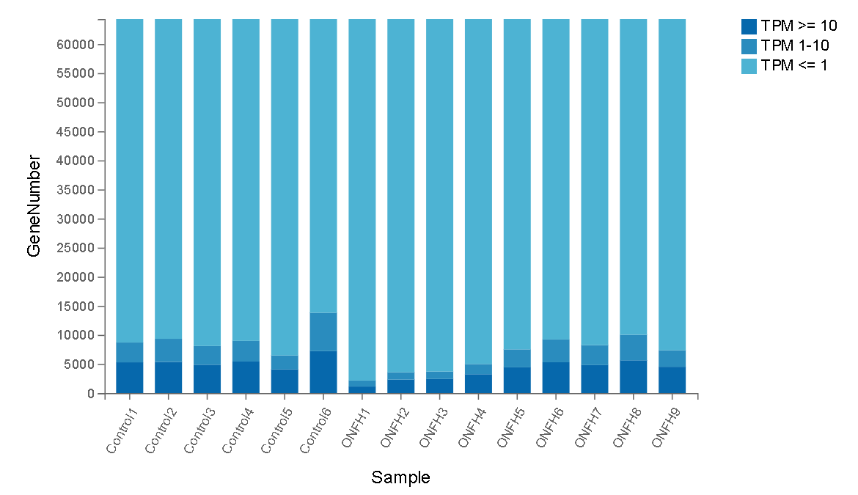


**Supplementary Figure 11.** Expression Stacked Bar Chart.

**2.** **Supplementary Tables**

**Supplementary Table 1.** Clinical Information of Samples

| ID | Age (years) | Sex |
| --- | --- | --- |
| Patient-1 | 52 | Male |
| Patient-2 | 51 | Male |
| Patient-3 | 61 | Male |
| Patient-4 | 64 | Female |
| Patient-5 | 48 | Female |
| Patient-6 | 66 | Male |
| Patient-7 | 77 | Female |
| Patient-8 | 50 | Male |
| Patient-9 | 59 | Male |
| Mean | 58.67 | -- |
| SD | 9.46 | -- |
| Control-1 | 68 | Female |
| Control-2 | 67 | Female |
| Control-3 | 55 | Male |
| Control-4 | 68 | Male |
| Control-5 | 49 | Male |
| Control-6 | 73 | Male |
| Mean | 63.33 | -- |
| SD | 9.22 | -- |

**Supplementary Table 2.** Clean Reads Quality statistics.

| Sample | Total Raw  Reads (M) | Total Clean  Reads (M) | Total Clean  Bases (Gb) | Clean Reads  Q20 (%) | Clean Reads  Q30 (%) | Clean Reads  Ratio (%) |
| --- | --- | --- | --- | --- | --- | --- |
| Control1 | 147.43 | 113.37 | 11.34 | 98.02 | 94.41 | 76.9 |
| Control2 | 115.09 | 101.11 | 10.11 | 97.81 | 93.87 | 87.85 |
| Control3 | 124.94 | 111.32 | 11.13 | 97.89 | 94.11 | 89.1 |
| Control4 | 116.97 | 105.95 | 10.6 | 97.88 | 94.08 | 90.58 |
| Control5 | 134.93 | 112.67 | 11.27 | 97.96 | 94.25 | 83.5 |
| Control6 | 122.53 | 109.07 | 10.91 | 97.91 | 94.11 | 89.01 |
| ONFH1 | 129.21 | 96.66 | 9.67 | 97.52 | 92.95 | 74.81 |
| ONFH2 | 134.64 | 111.56 | 11.16 | 97.73 | 93.69 | 82.86 |
| ONFH3 | 134.93 | 112.23 | 11.22 | 98.01 | 94.48 | 83.18 |
| ONFH4 | 142.43 | 112.55 | 11.26 | 97.94 | 94.23 | 79.02 |
| ONFH5 | 137.43 | 115.07 | 11.51 | 98.04 | 94.44 | 83.73 |
| ONFH6 | 132.43 | 115.87 | 11.59 | 97.95 | 94.16 | 87.5 |
| ONFH7 | 137.43 | 113.47 | 11.35 | 98.14 | 94.69 | 82.57 |
| ONFH8 | 116.39 | 98.39 | 9.84 | 98.05 | 94.48 | 84.53 |
| ONFH9 | 127.44 | 109.73 | 10.97 | 98.05 | 94.42 | 86.1 |

*Total Raw Reads, total amount of raw sequencing data; Total Clean Reads, number of reads retained after filtering; Total Clean Bases, number of bases in the filtered data; Clean Reads Q20, proportion of bases in clean reads with a sequencing accuracy >99%; Clean Reads Q30, proportion of bases in clean reads with a sequencing accuracy >99.9%; Clean Reads Ratio, proportion of total clean reads to total raw reads. For all samples, clean reads Q20 exceeded 95%, and clean reads Q30 exceeded 90%.*

**Supplementary Table 3.** Reference genome matching results.

| Sample | Total Clean Reads (M) | Total Mapping (%) | Uniquely Mapping (%) |
| --- | --- | --- | --- |
| Control1 | 113.37 | 56.93 | 44.14 |
| Control2 | 101.11 | 73.53 | 65.27 |
| Control3 | 111.32 | 76.21 | 64.84 |
| Control4 | 105.95 | 72.84 | 65.29 |
| Control5 | 112.67 | 51.44 | 45.65 |
| Control6 | 109.07 | 79.34 | 70.65 |
| ONFH1 | 96.66 | 44.86 | 36.83 |
| ONFH2 | 111.56 | 37.35 | 29.76 |
| ONFH3 | 112.23 | 56.69 | 46.78 |
| ONFH4 | 112.55 | 65.57 | 50.72 |
| ONFH5 | 115.07 | 68.63 | 54.41 |
| ONFH6 | 115.87 | 55.75 | 45.03 |
| ONFH7 | 113.47 | 75.45 | 57.75 |
| ONFH8 | 98.39 | 74.36 | 59.91 |
| ONFH9 | 109.73 | 64.96 | 54.18 |

*Total Clean Reads, number of reads retained after filtering; Total Mapping, percentage of reads mapped to the reference genome; Uniquely Mapped, percentage of reads mapped to a single unique location on the reference genome.*

**Supplementary Table 4.** Reference gene matching results.

| Sample | Total Clean Reads (M) | Total Mapping(%) | Uniquely Mapping(%) |
| --- | --- | --- | --- |
| Control1 | 113.37 | 21.51 | 9.89 |
| Control2 | 101.11 | 48.58 | 32.07 |
| Control3 | 111.32 | 45.68 | 25.45 |
| Control4 | 105.95 | 45.79 | 28.94 |
| Control5 | 112.67 | 34.65 | 23.33 |
| Control6 | 109.07 | 53.39 | 34.46 |
| ONFH1 | 96.66 | 3.69 | 2.01 |
| ONFH2 | 111.56 | 7.48 | 4.21 |
| ONFH3 | 112.23 | 24.84 | 7.63 |
| ONFH4 | 112.55 | 26.98 | 9.01 |
| ONFH5 | 115.07 | 28.11 | 10.23 |
| ONFH6 | 115.87 | 29.69 | 13.89 |
| ONFH7 | 113.47 | 41.77 | 10.71 |
| ONFH8 | 98.39 | 43.19 | 11.52 |
| ONFH9 | 109.73 | 32.47 | 11.12 |

*Total Clean Reads, number of reads retained after filtering; Total Mapping, percentage of reads mapped to the reference genome; Uniquely Mapped, percentage of reads mapped to a single unique location on the reference genome.*

**Supplementary Table 5.** Differential expression of immune-related lncRNAs and mRNAs in the ceRNA network.

| Symbol | Type | Regulation | log2FoldChange | Q-value |
| --- | --- | --- | --- | --- |
| MRPS30-DT | lncRNA | UP | 6.527571158 | 0.024635353 |
| LINC01106 | lncRNA | UP | 6.631867245 | 0.035579441 |
| MIR100HG | lncRNA | UP | 7.103866475 | 6.66E-04 |
| PELATON | lncRNA | DOWN | -6.954937628 | 0.039044083 |
| WDR11-AS1 | lncRNA | DOWN | -6.123165599 | 0.027309225 |
| BIRC5 | mRNA | UP | 11.40692567 | 0.00108962 |
| KIF14 | mRNA | UP | 7.030447288 | 0.031986185 |
| SEM1 | mRNA | UP | 7.643349733 | 0.037937887 |
| SPOP | mRNA | UP | 9.033501565 | 0.006052912 |
| BTRC | mRNA | DOWN | -7.722097485 | 0.004505844 |
| CD1D | mRNA | DOWN | -21.60084346 | 1.85E-11 |
| CD22 | mRNA | DOWN | -25.24718828 | 6.04E-15 |
| TNF | mRNA | DOWN | -13.76242018 | 9.96E-05 |

**Supplementary Table 6.** lncRNA ceRNA.

| lncRNA | miRNA | mRNA | lncRNA | miRNA | mRNA |
| --- | --- | --- | --- | --- | --- |
| LINC00630 | hsa-miR-542-3p | FHIP2A | PELATON | hsa-miR-449c-5p | TSEN15 |
| LINC00630 | hsa-miR-542-3p | AFAP1L1 | PELATON | hsa-miR-449c-5p | DIPK2A |
| LINC00630 | hsa-miR-542-3p | DPY19L3 | PELATON | hsa-miR-449c-5p | BAALC |
| LINC00630 | hsa-miR-24-3p | VASH1 | PELATON | hsa-miR-449c-5p | BCLAF3 |
| LINC00630 | hsa-miR-24-3p | NMNAT1 | PELATON | hsa-miR-449c-5p | MON2 |
| LINC00630 | hsa-miR-24-3p | PARN | PELATON | hsa-miR-449c-5p | CASP6 |
| LINC00630 | hsa-miR-24-3p | PLCH1 | PELATON | hsa-miR-449c-5p | MIER3 |
| LINC00630 | hsa-miR-24-3p | POLR2F | PELATON | hsa-miR-449c-5p | MFSD8 |
| LINC00630 | hsa-miR-24-3p | PABIR3 | PELATON | hsa-miR-449c-5p | ZNF548 |
| LINC00630 | hsa-miR-24-3p | CASK | PELATON | hsa-miR-449c-5p | ST6GALNAC3 |
| LINC00630 | hsa-miR-24-3p | MFF | PELATON | hsa-miR-449c-5p | TNS2 |
| LINC00630 | hsa-miR-24-3p | ZSCAN26 | PELATON | hsa-miR-449c-5p | CD200R1 |
| LINC00630 | hsa-miR-24-3p | P2RY13 | PELATON | hsa-miR-449c-5p | RIMKLA |
| LINC00630 | hsa-miR-24-3p | AGO3 | PELATON | hsa-miR-449c-5p | C16orf54 |
| LINC00630 | hsa-miR-24-3p | ARL5B | PELATON | hsa-miR-449c-5p | CFAP418 |
| LINC00630 | hsa-miR-24-3p | PRICKLE2 | PELATON | hsa-miR-449c-5p | AGBL3 |
| LINC00630 | hsa-miR-24-3p | SPOP | PELATON | hsa-miR-449c-5p | NAA20 |
| LINC00630 | hsa-miR-24-3p | PREPL | PELATON | hsa-miR-449c-5p | MCM4 |
| LINC00630 | hsa-miR-24-3p | ZFP30 | PELATON | hsa-miR-449c-5p | PRNP |
| LINC00630 | hsa-miR-24-3p | PNKD | PELATON | hsa-miR-449c-5p | UBE2J2 |
| LINC00630 | hsa-miR-24-3p | GPATCH2L | PELATON | hsa-miR-449c-5p | ADAMTS6 |
| LINC00630 | hsa-miR-24-3p | TRIM68 | PELATON | hsa-miR-449c-5p | POP5 |
| LINC00630 | hsa-miR-24-3p | APPL2 | PELATON | hsa-miR-449c-5p | LANCL3 |
| LINC00630 | hsa-miR-24-3p | HHAT | PELATON | hsa-miR-449c-5p | CENPS |
| LINC00630 | hsa-miR-24-3p | BCL7B | PELATON | hsa-miR-449c-5p | IL17REL |
| LINC00630 | hsa-miR-24-3p | ASH2L | PELATON | hsa-miR-449c-5p | PIGV |
| LINC00630 | hsa-miR-24-3p | COP1 | PELATON | hsa-miR-449c-5p | ZNF142 |
| LINC00630 | hsa-miR-24-3p | FLACC1 | PELATON | hsa-miR-449c-5p | CCR5 |
| LINC00630 | hsa-miR-24-3p | OR14J1 | PELATON | hsa-miR-449c-5p | CARHSP1 |
| LINC00630 | hsa-miR-24-3p | SLC25A28 | PELATON | hsa-miR-449c-5p | TMEM97 |
| LINC00630 | hsa-miR-24-3p | SHISA9 | PELATON | hsa-miR-449c-5p | DEPDC5 |
| LINC00630 | hsa-miR-24-3p | BIRC5 | PELATON | hsa-miR-449c-5p | DELE1 |
| LINC00630 | hsa-miR-24-3p | SLC5A3 | PELATON | hsa-miR-449c-5p | EPM2AIP1 |
| LINC00630 | hsa-miR-24-3p | MCTS1 | PELATON | hsa-miR-449c-5p | RPRD2 |
| LINC00630 | hsa-miR-24-3p | RAB3D | PELATON | hsa-miR-449c-5p | NCAPH |
| LINC00630 | hsa-miR-24-3p | NTPCR | PELATON | hsa-miR-449c-5p | GORASP2 |
| LINC00630 | hsa-miR-24-3p | MIEN1 | PELATON | hsa-miR-449c-5p | TRPC4AP |
| LINC00630 | hsa-miR-24-3p | ZNF441 | PELATON | hsa-miR-449c-5p | RBM7 |
| MRPS30-DT | hsa-miR-27b-3p | PLCH1 | PELATON | hsa-miR-449c-5p | PPME1 |
| MRPS30-DT | hsa-miR-27b-3p | KNDC1 | PELATON | hsa-miR-449c-5p | GPN3 |
| MRPS30-DT | hsa-miR-27b-3p | ARMCX5-GPRASP2 | PELATON | hsa-miR-449c-5p | ZNF571 |
| MRPS30-DT | hsa-miR-27b-3p | CMC2 | PELATON | hsa-miR-449c-5p | AMMECR1L |
| MRPS30-DT | hsa-miR-27b-3p | BTNL9 | PELATON | hsa-miR-449c-5p | EGR3 |
| MRPS30-DT | hsa-miR-27b-3p | HHAT | PELATON | hsa-miR-449c-5p | ADK |
| MRPS30-DT | hsa-miR-27b-3p | PARN | PELATON | hsa-miR-449c-5p | ATF7 |
| MRPS30-DT | hsa-miR-27b-3p | IKZF5 | PELATON | hsa-miR-449c-5p | LRCH2 |
| MRPS30-DT | hsa-miR-27b-3p | PTGFRN | PELATON | hsa-miR-449c-5p | AP4E1 |
| MRPS30-DT | hsa-miR-27b-3p | DPY19L3 | PELATON | hsa-miR-449c-5p | C19orf12 |
| MRPS30-DT | hsa-miR-27b-3p | MCTS1 | PELATON | hsa-miR-449c-5p | TMCO1 |
| MRPS30-DT | hsa-miR-27b-3p | NTPCR | PELATON | hsa-miR-449c-5p | ZNF43 |
| MRPS30-DT | hsa-miR-27b-3p | DEDD | PELATON | hsa-miR-449c-5p | URM1 |
| MRPS30-DT | hsa-miR-27b-3p | OARD1 | PELATON | hsa-miR-449c-5p | CRIP2 |
| MRPS30-DT | hsa-miR-27b-3p | CYP1B1 | PELATON | hsa-miR-449c-5p | PUS7L |
| MRPS30-DT | hsa-miR-103a-3p | MICU2 | PELATON | hsa-miR-449c-5p | ARFGAP1 |
| MRPS30-DT | hsa-miR-103a-3p | TFR2 | PELATON | hsa-miR-449c-5p | SIGMAR1 |
| MRPS30-DT | hsa-miR-103a-3p | MGAT4C | PELATON | hsa-miR-449c-5p | HECTD2 |
| MRPS30-DT | hsa-miR-103a-3p | CASK | PELATON | hsa-miR-449c-5p | RAB30 |
| MRPS30-DT | hsa-miR-103a-3p | ZSCAN26 | PELATON | hsa-miR-449c-5p | LOXL3 |
| MRPS30-DT | hsa-miR-103a-3p | KAT6B | PELATON | hsa-miR-449c-5p | CHST11 |
| MRPS30-DT | hsa-miR-103a-3p | SPOP | PELATON | hsa-miR-449c-5p | KLHL7 |
| MRPS30-DT | hsa-miR-103a-3p | PABIR3 | PELATON | hsa-miR-449c-5p | SIPA1L2 |
| MRPS30-DT | hsa-miR-103a-3p | PNKD | PELATON | hsa-miR-449c-5p | DIDO1 |
| MRPS30-DT | hsa-miR-103a-3p | MFF | PELATON | hsa-miR-449c-5p | RRAGC |
| MRPS30-DT | hsa-miR-103a-3p | NOLC1 | PELATON | hsa-miR-449c-5p | FEZ1 |
| MRPS30-DT | hsa-miR-103a-3p | TBCK | PELATON | hsa-miR-449c-5p | SIKE1 |
| MRPS30-DT | hsa-miR-103a-3p | GPC6 | PELATON | hsa-miR-449c-5p | PHAF1 |
| MRPS30-DT | hsa-miR-103a-3p | SLC5A3 | PELATON | hsa-miR-449c-5p | ARHGAP39 |
| MRPS30-DT | hsa-miR-103a-3p | MCTS1 | PELATON | hsa-miR-449c-5p | B4GALT2 |
| MRPS30-DT | hsa-miR-103a-3p | ING2 | PELATON | hsa-miR-449c-5p | FCRL5 |
| MRPS30-DT | hsa-miR-103a-3p | DEDD | PELATON | hsa-miR-449c-5p | NGLY1 |
| MRPS30-DT | hsa-miR-103a-3p | PARN | PELATON | hsa-miR-449c-5p | DSN1 |
| MRPS30-DT | hsa-miR-103a-3p | SLC3A1 | PELATON | hsa-miR-449c-5p | NRG1 |
| MRPS30-DT | hsa-miR-103a-3p | POLR2J2 | PELATON | hsa-miR-449c-5p | SMIM12 |
| MRPS30-DT | hsa-miR-103a-3p | GPATCH2L | PELATON | hsa-miR-449c-5p | TADA2A |
| MRPS30-DT | hsa-miR-27a-3p | CMC2 | PELATON | hsa-miR-449c-5p | PLEKHG7 |
| MRPS30-DT | hsa-miR-27a-3p | SCFD2 | PELATON | hsa-miR-449c-5p | FCRLA |
| MRPS30-DT | hsa-miR-27a-3p | CNBD1 | PELATON | hsa-miR-449c-5p | PEX16 |
| MRPS30-DT | hsa-miR-27a-3p | POLR3H | PELATON | hsa-miR-449c-5p | ABCG2 |
| MRPS30-DT | hsa-miR-27a-3p | AGO3 | PELATON | hsa-miR-449c-5p | LPAR4 |
| MRPS30-DT | hsa-miR-27a-3p | OR14J1 | PELATON | hsa-miR-449c-5p | LIPG |
| MRPS30-DT | hsa-miR-27a-3p | OARD1 | PELATON | hsa-miR-449c-5p | PPP2R5D |
| MRPS30-DT | hsa-miR-107 | BCL7B | PELATON | hsa-miR-449c-5p | TUBGCP3 |
| MRPS30-DT | hsa-miR-107 | TFR2 | PELATON | hsa-miR-449c-5p | PHTF1 |
| MRPS30-DT | hsa-miR-107 | RIMS2 | PELATON | hsa-miR-449c-5p | LYVE1 |
| MRPS30-DT | hsa-miR-107 | MGAT4C | PELATON | hsa-miR-449c-5p | ZNF212 |
| MRPS30-DT | hsa-miR-107 | CMC2 | PELATON | hsa-miR-449c-5p | MAPRE1 |
| MRPS30-DT | hsa-miR-107 | CASK | PELATON | hsa-miR-449c-5p | HSBP1 |
| MRPS30-DT | hsa-miR-107 | ZSCAN26 | PELATON | hsa-miR-449c-5p | EZH1 |
| MRPS30-DT | hsa-miR-107 | POLR3G | PELATON | hsa-miR-449c-5p | EIF6 |
| MRPS30-DT | hsa-miR-107 | SPOP | PELATON | hsa-miR-449c-5p | RNASE2 |
| MRPS30-DT | hsa-miR-107 | VASH1 | PELATON | hsa-miR-449c-5p | SRPK1 |
| MRPS30-DT | hsa-miR-107 | PNKD | PELATON | hsa-miR-449c-5p | UGDH |
| MRPS30-DT | hsa-miR-107 | GPATCH2L | PELATON | hsa-miR-449c-5p | ZNF264 |
| MRPS30-DT | hsa-miR-107 | ASH2L | PELATON | hsa-miR-449c-5p | CDC14A |
| MRPS30-DT | hsa-miR-107 | MFF | PELATON | hsa-miR-449c-5p | GALNT2 |
| MRPS30-DT | hsa-miR-107 | DHRS4 | PELATON | hsa-miR-449c-5p | HUS1 |
| MRPS30-DT | hsa-miR-107 | PTGFRN | PELATON | hsa-miR-449c-5p | C1orf174 |
| MRPS30-DT | hsa-miR-107 | SHISA9 | PELATON | hsa-miR-449c-5p | EEF1AKMT2 |
| MRPS30-DT | hsa-miR-107 | PREPL | PELATON | hsa-miR-449c-5p | PALS2 |
| MRPS30-DT | hsa-miR-107 | SLC5A3 | PELATON | hsa-miR-449c-5p | MPDU1 |
| MRPS30-DT | hsa-miR-107 | ING2 | PELATON | hsa-miR-449c-5p | BEX4 |
| MRPS30-DT | hsa-miR-107 | DEDD | PELATON | hsa-miR-449c-5p | PLEKHM3 |
| MRPS30-DT | hsa-miR-107 | PARN | PELATON | hsa-miR-449c-5p | TOX2 |
| MRPS30-DT | hsa-miR-107 | SLC3A1 | PELATON | hsa-miR-449c-5p | TMEM192 |
| MRPS30-DT | hsa-miR-107 | FHIP2A | PELATON | hsa-miR-449c-5p | CLDN22 |
| MRPS30-DT | hsa-miR-107 | NTPCR | PELATON | hsa-miR-449c-5p | NCBP3 |
| MRPS30-DT | hsa-miR-1301-3p | CMC2 | PELATON | hsa-miR-449c-5p | SLC2A5 |
| MRPS30-DT | hsa-miR-1301-3p | GPC6 | PELATON | hsa-miR-449c-5p | RAMAC |
| MRPS30-DT | hsa-miR-1301-3p | PRICKLE2 | PELATON | hsa-miR-449c-5p | MAK16 |
| MRPS30-DT | hsa-miR-1301-3p | SEM1 | PELATON | hsa-miR-449c-5p | CASP10 |
| MRPS30-DT | hsa-miR-1301-3p | KIF14 | PELATON | hsa-miR-449c-5p | PURB |
| MRPS30-DT | hsa-miR-1301-3p | ZFP30 | PELATON | hsa-miR-449c-5p | ZFHX2 |
| MRPS30-DT | hsa-miR-1301-3p | DAAM2 | PELATON | hsa-miR-449c-5p | INPP5K |
| MRPS30-DT | hsa-miR-1301-3p | LSM4 | PELATON | hsa-miR-449c-5p | ZNF483 |
| MRPS30-DT | hsa-miR-1301-3p | DDIT4 | PELATON | hsa-miR-449c-5p | MPLKIP |
| MRPS30-DT | hsa-miR-1301-3p | AGO3 | PELATON | hsa-miR-449c-5p | TSTD2 |
| MRPS30-DT | hsa-miR-1301-3p | SLC25A28 | PELATON | hsa-miR-449c-5p | C15orf40 |
| MRPS30-DT | hsa-miR-1301-3p | ZNF605 | PELATON | hsa-miR-449c-5p | HMG20A |
| MRPS30-DT | hsa-miR-1301-3p | BIRC5 | PELATON | hsa-miR-449c-5p | DOK3 |
| MRPS30-DT | hsa-miR-1301-3p | HHAT | PELATON | hsa-miR-449c-5p | ASB8 |
| MRPS30-DT | hsa-miR-1301-3p | PREPL | PELATON | hsa-miR-449c-5p | BLMH |
| MRPS30-DT | hsa-miR-1301-3p | SLC5A3 | PELATON | hsa-miR-449c-5p | SLC25A20 |
| MRPS30-DT | hsa-miR-1301-3p | BCL7B | PELATON | hsa-miR-449c-5p | IGF2R |
| MRPS30-DT | hsa-miR-1301-3p | FZD5 | PELATON | hsa-miR-449c-5p | C17orf97 |
| MRPS30-DT | hsa-miR-1301-3p | RAB3D | PELATON | hsa-miR-449c-5p | LAT |
| MRPS30-DT | hsa-miR-1301-3p | SLC3A1 | PELATON | hsa-miR-449c-5p | GOLGA8B |
| MRPS30-DT | hsa-miR-1301-3p | OARD1 | PELATON | hsa-miR-449c-5p | TM2D2 |
| LINC01106 | hsa-miR-34a-5p | MGAT4C | PELATON | hsa-miR-449c-5p | TRAF5 |
| LINC01106 | hsa-miR-34a-5p | MAP1A | PELATON | hsa-miR-449c-5p | HEY1 |
| LINC01106 | hsa-miR-34a-5p | FBH1 | PELATON | hsa-miR-941 | TMCO1 |
| LINC01106 | hsa-miR-34a-5p | PPARGC1A | PELATON | hsa-miR-941 | ATF7 |
| LINC01106 | hsa-miR-34a-5p | FAM114A1 | PELATON | hsa-miR-941 | ABL1 |
| LINC01106 | hsa-miR-34a-5p | PLCH1 | PELATON | hsa-miR-941 | AMMECR1L |
| LINC01106 | hsa-miR-34a-5p | ARMCX5-GPRASP2 | PELATON | hsa-miR-941 | ASCC1 |
| LINC01106 | hsa-miR-34a-5p | CMC2 | PELATON | hsa-miR-941 | EFCAB11 |
| LINC01106 | hsa-miR-34a-5p | CASK | PELATON | hsa-miR-941 | GOT2 |
| LINC01106 | hsa-miR-34a-5p | RASA4B | PELATON | hsa-miR-941 | UBE4A |
| LINC01106 | hsa-miR-34a-5p | ARL5B | PELATON | hsa-miR-941 | CLUAP1 |
| LINC01106 | hsa-miR-34a-5p | DHX57 | PELATON | hsa-miR-941 | NSUN7 |
| LINC01106 | hsa-miR-34a-5p | SEM1 | PELATON | hsa-miR-941 | RNF144A |
| LINC01106 | hsa-miR-34a-5p | GPATCH2L | PELATON | hsa-miR-941 | GDAP1 |
| LINC01106 | hsa-miR-34a-5p | PTPRB | PELATON | hsa-miR-941 | MEA1 |
| LINC01106 | hsa-miR-34a-5p | NVL | PELATON | hsa-miR-941 | HUS1 |
| LINC01106 | hsa-miR-34a-5p | ZNF512 | PELATON | hsa-miR-941 | ZNF763 |
| LINC01106 | hsa-miR-34a-5p | IKZF5 | PELATON | hsa-miR-941 | AGBL3 |
| LINC01106 | hsa-miR-34a-5p | POLR3H | PELATON | hsa-miR-941 | DEFB112 |
| LINC01106 | hsa-miR-34a-5p | AFAP1L1 | PELATON | hsa-miR-941 | ZC3H12C |
| LINC01106 | hsa-miR-34a-5p | TBCK | PELATON | hsa-miR-941 | HDAC8 |
| LINC01106 | hsa-miR-34a-5p | ERAL1 | PELATON | hsa-miR-941 | ANKS1A |
| LINC01106 | hsa-miR-34a-5p | SLC5A3 | PELATON | hsa-miR-941 | RNF32 |
| LINC01106 | hsa-miR-34a-5p | RAB3D | PELATON | hsa-miR-941 | CASP6 |
| LINC01106 | hsa-miR-34a-5p | PABIR3 | PELATON | hsa-miR-941 | SLC1A3 |
| LINC01106 | hsa-miR-34a-5p | ADH5 | PELATON | hsa-miR-941 | GSTM4 |
| LINC01106 | hsa-miR-34a-5p | BIRC5 | PELATON | hsa-miR-941 | IL12RB2 |
| FAM201A | hsa-miR-708-5p | DEDD | PELATON | hsa-miR-941 | ST7L |
| FAM201A | hsa-miR-708-5p | FAM114A1 | PELATON | hsa-miR-941 | ZNF610 |
| FAM201A | hsa-miR-708-5p | KNDC1 | PELATON | hsa-miR-941 | TCF3 |
| FAM201A | hsa-miR-708-5p | RIMS2 | PELATON | hsa-miR-941 | MBD5 |
| FAM201A | hsa-miR-708-5p | RASA4B | PELATON | hsa-miR-941 | RAPH1 |
| FAM201A | hsa-miR-708-5p | PRICKLE2 | PELATON | hsa-miR-941 | GORASP1 |
| FAM201A | hsa-miR-708-5p | ABCG1 | PELATON | hsa-miR-941 | PTGR2 |
| FAM201A | hsa-miR-708-5p | CMC2 | PELATON | hsa-miR-941 | MIER3 |
| FAM201A | hsa-miR-708-5p | CRTC3 | PELATON | hsa-miR-941 | ELAPOR2 |
| FAM201A | hsa-miR-708-5p | NMNAT1 | PELATON | hsa-miR-941 | ST6GALNAC3 |
| FAM201A | hsa-miR-708-5p | OR14J1 | PELATON | hsa-miR-941 | IQCK |
| FAM201A | hsa-miR-708-5p | SHISA9 | PELATON | hsa-miR-941 | MED19 |
| FAM201A | hsa-miR-708-5p | NTPCR | PELATON | hsa-miR-941 | NAP1L5 |
| FAM201A | hsa-miR-221-3p | GPC6 | PELATON | hsa-miR-941 | TNS2 |
| FAM201A | hsa-miR-221-3p | KNDC1 | PELATON | hsa-miR-941 | CD200R1 |
| FAM201A | hsa-miR-221-3p | RIMS2 | PELATON | hsa-miR-941 | MRPL42 |
| FAM201A | hsa-miR-221-3p | MGAT4C | PELATON | hsa-miR-941 | UNC45B |
| FAM201A | hsa-miR-221-3p | CMC2 | PELATON | hsa-miR-941 | TYSND1 |
| FAM201A | hsa-miR-221-3p | CBWD5 | PELATON | hsa-miR-941 | NOMO2 |
| FAM201A | hsa-miR-221-3p | AGO3 | PELATON | hsa-miR-941 | SLC9A9 |
| FAM201A | hsa-miR-221-3p | ZNF605 | PELATON | hsa-miR-941 | DPY19L2 |
| FAM201A | hsa-miR-221-3p | PRDM10 | PELATON | hsa-miR-941 | IL5RA |
| FAM201A | hsa-miR-221-3p | TRIM68 | PELATON | hsa-miR-941 | MARCHF3 |
| FAM201A | hsa-miR-221-3p | POLR3H | PELATON | hsa-miR-941 | GEN1 |
| FAM201A | hsa-miR-221-3p | OR14J1 | PELATON | hsa-miR-941 | PLCG1 |
| FAM201A | hsa-miR-221-3p | SLC5A3 | PELATON | hsa-miR-941 | GDPD4 |
| FAM201A | hsa-miR-221-3p | FZD5 | PELATON | hsa-miR-941 | SHC1 |
| FAM201A | hsa-miR-221-3p | DAAM2 | PELATON | hsa-miR-941 | MITF |
| FAM201A | hsa-miR-221-3p | MIEN1 | PELATON | hsa-miR-941 | C1orf122 |
| FAM201A | hsa-miR-221-3p | OARD1 | PELATON | hsa-miR-941 | THAP1 |
| FAM201A | hsa-miR-101-3p | ARL5B | PELATON | hsa-miR-941 | PIGV |
| FAM201A | hsa-miR-101-3p | ZNF512 | PELATON | hsa-miR-941 | MMAA |
| FAM201A | hsa-miR-28-5p | RIMS2 | PELATON | hsa-miR-941 | ZSCAN20 |
| FAM201A | hsa-miR-28-5p | ZNF512 | PELATON | hsa-miR-941 | ZNF142 |
| FAM201A | hsa-miR-28-5p | PPARGC1A | PELATON | hsa-miR-941 | XCR1 |
| FAM201A | hsa-miR-28-5p | KNDC1 | PELATON | hsa-miR-941 | ADPRH |
| FAM201A | hsa-miR-28-5p | MGAT4C | PELATON | hsa-miR-941 | PPFIA4 |
| FAM201A | hsa-miR-28-5p | CMC2 | PELATON | hsa-miR-941 | PVRIG |
| FAM201A | hsa-miR-28-5p | EXOC8 | PELATON | hsa-miR-941 | NOMO1 |
| FAM201A | hsa-miR-28-5p | ZNF227 | PELATON | hsa-miR-941 | ZNF623 |
| FAM201A | hsa-miR-28-5p | PNKD | PELATON | hsa-miR-941 | CEP162 |
| FAM201A | hsa-miR-28-5p | GPATCH2L | PELATON | hsa-miR-941 | KLHDC10 |
| FAM201A | hsa-miR-28-5p | HHAT | PELATON | hsa-miR-941 | ACSBG1 |
| FAM201A | hsa-miR-28-5p | PARN | PELATON | hsa-miR-941 | RPRD2 |
| FAM201A | hsa-miR-28-5p | POLR2F | PELATON | hsa-miR-941 | PARM1 |
| FAM201A | hsa-miR-28-5p | CRTC3 | PELATON | hsa-miR-941 | MTMR9 |
| FAM201A | hsa-miR-28-5p | OR14J1 | PELATON | hsa-miR-941 | ZBTB7A |
| FAM201A | hsa-miR-28-5p | GPC6 | PELATON | hsa-miR-941 | NIP7 |
| FAM201A | hsa-miR-28-5p | MCTS1 | PELATON | hsa-miR-941 | SUFU |
| FAM201A | hsa-miR-28-5p | RAB3D | PELATON | hsa-miR-941 | GDE1 |
| FAM201A | hsa-miR-28-5p | LURAP1L | PELATON | hsa-miR-941 | TERF1 |
| FAM201A | hsa-miR-28-5p | DAAM2 | PELATON | hsa-miR-941 | GOLPH3L |
| FAM201A | hsa-miR-28-5p | MIEN1 | PELATON | hsa-miR-941 | DIDO1 |
| MIR100HG | hsa-miR-19b-3p | CASK | PELATON | hsa-miR-941 | TGFBR3 |
| MIR100HG | hsa-miR-19b-3p | CBLB | PELATON | hsa-miR-941 | C19orf12 |
| MIR100HG | hsa-miR-19b-3p | POLR3H | PELATON | hsa-miR-941 | KCTD1 |
| MIR100HG | hsa-miR-19b-3p | DPY19L3 | PELATON | hsa-miR-941 | PSMD9 |
| MIR100HG | hsa-miR-19b-3p | POLR3G | PELATON | hsa-miR-941 | JAM2 |
| MIR100HG | hsa-miR-19b-3p | RWDD3 | PELATON | hsa-miR-941 | ADGRE2 |
| MIR100HG | hsa-miR-19b-3p | RIOK1 | PELATON | hsa-miR-941 | PUS7L |
| MIR100HG | hsa-miR-19b-3p | GPATCH2L | PELATON | hsa-miR-941 | SLC39A1 |
| MIR100HG | hsa-miR-19b-3p | OARD1 | PELATON | hsa-miR-941 | CNIH4 |
| MIR100HG | hsa-miR-130b-3p | AGO3 | PELATON | hsa-miR-941 | LPAR4 |
| MIR100HG | hsa-miR-130b-3p | MGAT4C | PELATON | hsa-miR-941 | E2F6 |
| MIR100HG | hsa-miR-130b-3p | ZNF605 | PELATON | hsa-miR-941 | CD22 |
| MIR100HG | hsa-miR-130b-3p | PTGFRN | PELATON | hsa-miR-941 | HECTD2 |
| MIR100HG | hsa-miR-130b-3p | NMNAT1 | PELATON | hsa-miR-941 | RAB30 |
| MIR100HG | hsa-miR-130b-3p | SHISA9 | PELATON | hsa-miR-941 | STX6 |
| MIR100HG | hsa-miR-130b-3p | PREPL | PELATON | hsa-miR-941 | CDK2 |
| MIR100HG | hsa-miR-130b-3p | FZD5 | PELATON | hsa-miR-941 | PECR |
| MIR100HG | hsa-miR-130b-3p | RIOK1 | PELATON | hsa-miR-941 | KLHL4 |
| MIR100HG | hsa-miR-130b-3p | HMCN1 | PELATON | hsa-miR-941 | PNO1 |
| MIR100HG | hsa-miR-452-5p | KNDC1 | PELATON | hsa-miR-941 | PAIP2B |
| MIR100HG | hsa-miR-452-5p | CMC2 | PELATON | hsa-miR-941 | ABO |
| MIR100HG | hsa-miR-452-5p | ZNF66 | PELATON | hsa-miR-941 | NRIP3 |
| MIR100HG | hsa-miR-452-5p | CBLB | PELATON | hsa-miR-941 | ZBTB21 |
| MIR100HG | hsa-miR-452-5p | AGO3 | PELATON | hsa-miR-941 | RANBP10 |
| MIR100HG | hsa-miR-452-5p | PABIR3 | PELATON | hsa-miR-941 | CHD8 |
| MIR100HG | hsa-miR-452-5p | KLHL36 | PELATON | hsa-miR-941 | EPG5 |
| MIR100HG | hsa-miR-452-5p | OR14J1 | PELATON | hsa-miR-941 | TRIB3 |
| MIR100HG | hsa-miR-452-5p | SHISA9 | PELATON | hsa-miR-941 | RRAGD |
| MIR100HG | hsa-miR-452-5p | TBCK | PELATON | hsa-miR-941 | RBSN |
| MIR100HG | hsa-miR-452-5p | PPARGC1A | PELATON | hsa-miR-941 | USP46 |
| MIR100HG | hsa-miR-452-5p | MCTS1 | PELATON | hsa-miR-941 | DDA1 |
| MIR100HG | hsa-miR-219a-5p | PTPRB | PELATON | hsa-miR-941 | ZMAT4 |
| MIR100HG | hsa-miR-148b-3p | RIMS2 | PELATON | hsa-miR-941 | KLHL18 |
| MIR100HG | hsa-miR-148b-3p | SEM1 | PELATON | hsa-miR-941 | SIKE1 |
| MIR100HG | hsa-miR-148b-3p | ASH2L | PELATON | hsa-miR-941 | EFHC2 |
| MIR100HG | hsa-miR-148b-3p | PTGFRN | PELATON | hsa-miR-941 | TMX1 |
| MIR100HG | hsa-miR-148b-3p | CRTC3 | PELATON | hsa-miR-941 | DIS3L |
| MIR100HG | hsa-miR-148b-3p | KLHL36 | PELATON | hsa-miR-941 | DOK3 |
| MIR100HG | hsa-miR-148b-3p | OR14J1 | PELATON | hsa-miR-941 | NRG1 |
| MIR100HG | hsa-miR-148b-3p | DEDD | PELATON | hsa-miR-941 | ARHGAP4 |
| MIR100HG | hsa-miR-19a-3p | POLR3H | PELATON | hsa-miR-941 | SMIM12 |
| MIR100HG | hsa-miR-19a-3p | GPC6 | PELATON | hsa-miR-941 | TADA2A |
| MIR100HG | hsa-miR-19a-3p | RIOK1 | PELATON | hsa-miR-941 | SIGLEC10 |
| MIR100HG | hsa-miR-671-5p | CMC2 | PELATON | hsa-miR-941 | TK2 |
| MIR100HG | hsa-miR-671-5p | GPATCH2L | PELATON | hsa-miR-941 | AIM2 |
| MIR100HG | hsa-miR-671-5p | TBCK | PELATON | hsa-miR-941 | IL27RA |
| MIR100HG | hsa-miR-671-5p | PREPL | PELATON | hsa-miR-941 | NRF1 |
| MIR100HG | hsa-miR-671-5p | PABIR3 | PELATON | hsa-miR-941 | OMD |
| MIR100HG | hsa-miR-671-5p | PNKD | PELATON | hsa-miR-941 | UNC119 |
| MIR100HG | hsa-miR-671-5p | ARMCX5-GPRASP2 | PELATON | hsa-miR-941 | TRIM13 |
| MIR100HG | hsa-miR-671-5p | MGAT4C | PELATON | hsa-miR-941 | LIPG |
| MIR100HG | hsa-miR-671-5p | AGO3 | PELATON | hsa-miR-941 | PPP2R5D |
| MIR100HG | hsa-miR-671-5p | ARL5B | PELATON | hsa-miR-941 | LRRC41 |
| MIR100HG | hsa-miR-671-5p | ZNF605 | PELATON | hsa-miR-941 | BATF |
| MIR100HG | hsa-miR-671-5p | SPOP | PELATON | hsa-miR-941 | RSC1A1 |
| MIR100HG | hsa-miR-671-5p | SEM1 | PELATON | hsa-miR-941 | PNMA2 |
| MIR100HG | hsa-miR-671-5p | VASH1 | PELATON | hsa-miR-941 | ACOX1 |
| MIR100HG | hsa-miR-671-5p | HHAT | PELATON | hsa-miR-941 | TIMM10B |
| MIR100HG | hsa-miR-671-5p | SELPLG | PELATON | hsa-miR-941 | MID2 |
| MIR100HG | hsa-miR-671-5p | ZNF512 | PELATON | hsa-miR-941 | SPCS1 |
| MIR100HG | hsa-miR-671-5p | DCTN1 | PELATON | hsa-miR-941 | MRTFB |
| MIR100HG | hsa-miR-671-5p | MCTS1 | PELATON | hsa-miR-941 | ACP5 |
| MIR100HG | hsa-miR-671-5p | SLC3A1 | PELATON | hsa-miR-941 | ATP2B4 |
| MIR100HG | hsa-miR-671-5p | BTNL9 | PELATON | hsa-miR-941 | DLST |
| MIR100HG | hsa-miR-671-5p | FLACC1 | PELATON | hsa-miR-941 | ENO2 |
| MIR100HG | hsa-miR-671-5p | LMLN | PELATON | hsa-miR-941 | GZMK |
| MIR100HG | hsa-miR-671-5p | OCIAD2 | PELATON | hsa-miR-941 | RYK |
| PELATON | hsa-miR-34a-5p | FAM161B | PELATON | hsa-miR-941 | DVL3 |
| PELATON | hsa-miR-34a-5p | NF2 | PELATON | hsa-miR-941 | GALNT2 |
| PELATON | hsa-miR-34a-5p | PUS7L | PELATON | hsa-miR-941 | XPR1 |
| PELATON | hsa-miR-34a-5p | GNPDA1 | PELATON | hsa-miR-941 | EEF1AKMT2 |
| PELATON | hsa-miR-34a-5p | LRRIQ3 | PELATON | hsa-miR-941 | AFF3 |
| PELATON | hsa-miR-34a-5p | ZNF440 | PELATON | hsa-miR-941 | MTARC1 |
| PELATON | hsa-miR-34a-5p | ST7L | PELATON | hsa-miR-941 | TP53I11 |
| PELATON | hsa-miR-34a-5p | CPT2 | PELATON | hsa-miR-941 | ZNF483 |
| PELATON | hsa-miR-34a-5p | C16orf87 | PELATON | hsa-miR-941 | N4BP2L1 |
| PELATON | hsa-miR-34a-5p | DBN1 | PELATON | hsa-miR-941 | TMEM87A |
| PELATON | hsa-miR-34a-5p | MEA1 | PELATON | hsa-miR-941 | NCBP3 |
| PELATON | hsa-miR-34a-5p | TSEN15 | PELATON | hsa-miR-941 | PLIN2 |
| PELATON | hsa-miR-34a-5p | HUS1 | PELATON | hsa-miR-941 | ABRAXAS2 |
| PELATON | hsa-miR-34a-5p | MSR1 | PELATON | hsa-miR-941 | CCDC115 |
| PELATON | hsa-miR-34a-5p | MTX3 | PELATON | hsa-miR-941 | KBTBD8 |
| PELATON | hsa-miR-34a-5p | BAALC | PELATON | hsa-miR-941 | CBR4 |
| PELATON | hsa-miR-34a-5p | CDK5RAP1 | PELATON | hsa-miR-941 | CASP2 |
| PELATON | hsa-miR-34a-5p | ADGRL2 | PELATON | hsa-miR-941 | EAF1 |
| PELATON | hsa-miR-34a-5p | ATF7 | PELATON | hsa-miR-941 | IMP4 |
| PELATON | hsa-miR-34a-5p | ZNF763 | PELATON | hsa-miR-941 | GALNT15 |
| PELATON | hsa-miR-34a-5p | CRISP3 | PELATON | hsa-miR-941 | COX15 |
| PELATON | hsa-miR-34a-5p | ASCC1 | PELATON | hsa-miR-941 | PABIR1 |
| PELATON | hsa-miR-34a-5p | ALDH3A2 | PELATON | hsa-miR-941 | GABPB2 |
| PELATON | hsa-miR-34a-5p | ADK | PELATON | hsa-miR-941 | UPF1 |
| PELATON | hsa-miR-34a-5p | ZC3H12C | PELATON | hsa-miR-941 | LSAMP |
| PELATON | hsa-miR-34a-5p | MON2 | PELATON | hsa-miR-941 | EZH1 |
| PELATON | hsa-miR-34a-5p | ZNF233 | PELATON | hsa-miR-941 | DHRS7 |
| PELATON | hsa-miR-34a-5p | HDAC8 | PELATON | hsa-miR-941 | AGA |
| PELATON | hsa-miR-34a-5p | ANKS1A | PELATON | hsa-miR-941 | UMPS |
| PELATON | hsa-miR-34a-5p | TTC39B | PELATON | hsa-miR-941 | PHEX |
| PELATON | hsa-miR-34a-5p | CASP6 | PELATON | hsa-miR-941 | MED11 |
| PELATON | hsa-miR-34a-5p | IL12RB2 | PELATON | hsa-miR-941 | LMBRD2 |
| PELATON | hsa-miR-34a-5p | GLI3 | PELATON | hsa-miR-941 | TCAIM |
| PELATON | hsa-miR-34a-5p | ZNF706 | PELATON | hsa-miR-941 | FOXK1 |
| PELATON | hsa-miR-34a-5p | PLEKHA2 | PELATON | hsa-miR-941 | LINS1 |
| PELATON | hsa-miR-34a-5p | CAMSAP1 | WDR11-AS1 | hsa-miR-34a-5p | FAM161B |
| PELATON | hsa-miR-34a-5p | ACBD6 | WDR11-AS1 | hsa-miR-34a-5p | NF2 |
| PELATON | hsa-miR-34a-5p | MRTFB | WDR11-AS1 | hsa-miR-34a-5p | PUS7L |
| PELATON | hsa-miR-34a-5p | ZNF610 | WDR11-AS1 | hsa-miR-34a-5p | GNPDA1 |
| PELATON | hsa-miR-34a-5p | TCF3 | WDR11-AS1 | hsa-miR-34a-5p | LRRIQ3 |
| PELATON | hsa-miR-34a-5p | SIGLEC10 | WDR11-AS1 | hsa-miR-34a-5p | ZNF440 |
| PELATON | hsa-miR-34a-5p | GALM | WDR11-AS1 | hsa-miR-34a-5p | ST7L |
| PELATON | hsa-miR-34a-5p | CNEP1R1 | WDR11-AS1 | hsa-miR-34a-5p | CPT2 |
| PELATON | hsa-miR-34a-5p | MMAA | WDR11-AS1 | hsa-miR-34a-5p | C16orf87 |
| PELATON | hsa-miR-34a-5p | RIMKLA | WDR11-AS1 | hsa-miR-34a-5p | DBN1 |
| PELATON | hsa-miR-34a-5p | ARHGEF15 | WDR11-AS1 | hsa-miR-34a-5p | MEA1 |
| PELATON | hsa-miR-34a-5p | IL1RN | WDR11-AS1 | hsa-miR-34a-5p | TSEN15 |
| PELATON | hsa-miR-34a-5p | IL5RA | WDR11-AS1 | hsa-miR-34a-5p | HUS1 |
| PELATON | hsa-miR-34a-5p | CCDC71L | WDR11-AS1 | hsa-miR-34a-5p | MSR1 |
| PELATON | hsa-miR-34a-5p | SESTD1 | WDR11-AS1 | hsa-miR-34a-5p | MTX3 |
| PELATON | hsa-miR-34a-5p | PPP2R5D | WDR11-AS1 | hsa-miR-34a-5p | BAALC |
| PELATON | hsa-miR-34a-5p | CMTM7 | WDR11-AS1 | hsa-miR-34a-5p | CDK5RAP1 |
| PELATON | hsa-miR-34a-5p | ZNF714 | WDR11-AS1 | hsa-miR-34a-5p | ADGRL2 |
| PELATON | hsa-miR-34a-5p | GEN1 | WDR11-AS1 | hsa-miR-34a-5p | ATF7 |
| PELATON | hsa-miR-34a-5p | EFNA4 | WDR11-AS1 | hsa-miR-34a-5p | ZNF763 |
| PELATON | hsa-miR-34a-5p | TPRG1L | WDR11-AS1 | hsa-miR-34a-5p | CRISP3 |
| PELATON | hsa-miR-34a-5p | ELMO2 | WDR11-AS1 | hsa-miR-34a-5p | ASCC1 |
| PELATON | hsa-miR-34a-5p | ZDHHC16 | WDR11-AS1 | hsa-miR-34a-5p | ALDH3A2 |
| PELATON | hsa-miR-34a-5p | POP5 | WDR11-AS1 | hsa-miR-34a-5p | ADK |
| PELATON | hsa-miR-34a-5p | MLX | WDR11-AS1 | hsa-miR-34a-5p | ZC3H12C |
| PELATON | hsa-miR-34a-5p | CPM | WDR11-AS1 | hsa-miR-34a-5p | MON2 |
| PELATON | hsa-miR-34a-5p | LANCL3 | WDR11-AS1 | hsa-miR-34a-5p | ZNF233 |
| PELATON | hsa-miR-34a-5p | DSTYK | WDR11-AS1 | hsa-miR-34a-5p | HDAC8 |
| PELATON | hsa-miR-34a-5p | EEF2KMT | WDR11-AS1 | hsa-miR-34a-5p | ANKS1A |
| PELATON | hsa-miR-34a-5p | ZBTB46 | WDR11-AS1 | hsa-miR-34a-5p | TTC39B |
| PELATON | hsa-miR-34a-5p | IL17REL | WDR11-AS1 | hsa-miR-34a-5p | CASP6 |
| PELATON | hsa-miR-34a-5p | CD1D | WDR11-AS1 | hsa-miR-34a-5p | IL12RB2 |
| PELATON | hsa-miR-34a-5p | ZNF142 | WDR11-AS1 | hsa-miR-34a-5p | GLI3 |
| PELATON | hsa-miR-34a-5p | ADAM17 | WDR11-AS1 | hsa-miR-34a-5p | ZNF706 |
| PELATON | hsa-miR-34a-5p | USP49 | WDR11-AS1 | hsa-miR-34a-5p | PLEKHA2 |
| PELATON | hsa-miR-34a-5p | DPYS | WDR11-AS1 | hsa-miR-34a-5p | CAMSAP1 |
| PELATON | hsa-miR-34a-5p | ZFHX3 | WDR11-AS1 | hsa-miR-34a-5p | ACBD6 |
| PELATON | hsa-miR-34a-5p | CXCL16 | WDR11-AS1 | hsa-miR-34a-5p | MRTFB |
| PELATON | hsa-miR-34a-5p | ADPRH | WDR11-AS1 | hsa-miR-34a-5p | ZNF610 |
| PELATON | hsa-miR-34a-5p | SMIM20 | WDR11-AS1 | hsa-miR-34a-5p | TCF3 |
| PELATON | hsa-miR-34a-5p | ZBTB34 | WDR11-AS1 | hsa-miR-34a-5p | SIGLEC10 |
| PELATON | hsa-miR-34a-5p | RNF7 | WDR11-AS1 | hsa-miR-34a-5p | GALM |
| PELATON | hsa-miR-34a-5p | LAT | WDR11-AS1 | hsa-miR-34a-5p | CNEP1R1 |
| PELATON | hsa-miR-34a-5p | LCMT2 | WDR11-AS1 | hsa-miR-34a-5p | MMAA |
| PELATON | hsa-miR-34a-5p | EPM2AIP1 | WDR11-AS1 | hsa-miR-34a-5p | RIMKLA |
| PELATON | hsa-miR-34a-5p | SUPT7L | WDR11-AS1 | hsa-miR-34a-5p | ARHGEF15 |
| PELATON | hsa-miR-34a-5p | CMTR1 | WDR11-AS1 | hsa-miR-34a-5p | IL1RN |
| PELATON | hsa-miR-34a-5p | MTMR9 | WDR11-AS1 | hsa-miR-34a-5p | IL5RA |
| PELATON | hsa-miR-34a-5p | AAR2 | WDR11-AS1 | hsa-miR-34a-5p | CCDC71L |
| PELATON | hsa-miR-34a-5p | GADD45B | WDR11-AS1 | hsa-miR-34a-5p | SESTD1 |
| PELATON | hsa-miR-34a-5p | MRPS17 | WDR11-AS1 | hsa-miR-34a-5p | PPP2R5D |
| PELATON | hsa-miR-34a-5p | NIP7 | WDR11-AS1 | hsa-miR-34a-5p | CMTM7 |
| PELATON | hsa-miR-34a-5p | IRAK4 | WDR11-AS1 | hsa-miR-34a-5p | ZNF714 |
| PELATON | hsa-miR-34a-5p | SUFU | WDR11-AS1 | hsa-miR-34a-5p | GEN1 |
| PELATON | hsa-miR-34a-5p | PHF20 | WDR11-AS1 | hsa-miR-34a-5p | EFNA4 |
| PELATON | hsa-miR-34a-5p | OSER1 | WDR11-AS1 | hsa-miR-34a-5p | TPRG1L |
| PELATON | hsa-miR-34a-5p | GDE1 | WDR11-AS1 | hsa-miR-34a-5p | ELMO2 |
| PELATON | hsa-miR-34a-5p | NKIRAS2 | WDR11-AS1 | hsa-miR-34a-5p | ZDHHC16 |
| PELATON | hsa-miR-34a-5p | THUMPD1 | WDR11-AS1 | hsa-miR-34a-5p | POP5 |
| PELATON | hsa-miR-34a-5p | POLR3E | WDR11-AS1 | hsa-miR-34a-5p | MLX |
| PELATON | hsa-miR-34a-5p | TMEM33 | WDR11-AS1 | hsa-miR-34a-5p | CPM |
| PELATON | hsa-miR-34a-5p | HMG20A | WDR11-AS1 | hsa-miR-34a-5p | LANCL3 |
| PELATON | hsa-miR-34a-5p | ARFGAP1 | WDR11-AS1 | hsa-miR-34a-5p | DSTYK |
| PELATON | hsa-miR-34a-5p | SIRT2 | WDR11-AS1 | hsa-miR-34a-5p | EEF2KMT |
| PELATON | hsa-miR-34a-5p | DIDO1 | WDR11-AS1 | hsa-miR-34a-5p | ZBTB46 |
| PELATON | hsa-miR-34a-5p | VCAM1 | WDR11-AS1 | hsa-miR-34a-5p | IL17REL |
| PELATON | hsa-miR-34a-5p | HSPE1-MOB4 | WDR11-AS1 | hsa-miR-34a-5p | CD1D |
| PELATON | hsa-miR-34a-5p | GK | WDR11-AS1 | hsa-miR-34a-5p | ZNF142 |
| PELATON | hsa-miR-34a-5p | CASP10 | WDR11-AS1 | hsa-miR-34a-5p | ADAM17 |
| PELATON | hsa-miR-34a-5p | NFIC | WDR11-AS1 | hsa-miR-34a-5p | USP49 |
| PELATON | hsa-miR-34a-5p | CDC6 | WDR11-AS1 | hsa-miR-34a-5p | DPYS |
| PELATON | hsa-miR-34a-5p | CERS6 | WDR11-AS1 | hsa-miR-34a-5p | ZFHX3 |
| PELATON | hsa-miR-34a-5p | TMCO1 | WDR11-AS1 | hsa-miR-34a-5p | CXCL16 |
| PELATON | hsa-miR-34a-5p | CYBRD1 | WDR11-AS1 | hsa-miR-34a-5p | ADPRH |
| PELATON | hsa-miR-34a-5p | UCK1 | WDR11-AS1 | hsa-miR-34a-5p | SMIM20 |
| PELATON | hsa-miR-34a-5p | TTPAL | WDR11-AS1 | hsa-miR-34a-5p | ZBTB34 |
| PELATON | hsa-miR-34a-5p | EIF6 | WDR11-AS1 | hsa-miR-34a-5p | RNF7 |
| PELATON | hsa-miR-34a-5p | TFAM | WDR11-AS1 | hsa-miR-34a-5p | LAT |
| PELATON | hsa-miR-34a-5p | TREML1 | WDR11-AS1 | hsa-miR-34a-5p | LCMT2 |
| PELATON | hsa-miR-34a-5p | AAGAB | WDR11-AS1 | hsa-miR-34a-5p | EPM2AIP1 |
| PELATON | hsa-miR-34a-5p | TARS2 | WDR11-AS1 | hsa-miR-34a-5p | SUPT7L |
| PELATON | hsa-miR-34a-5p | STK25 | WDR11-AS1 | hsa-miR-34a-5p | CMTR1 |
| PELATON | hsa-miR-34a-5p | LPAR4 | WDR11-AS1 | hsa-miR-34a-5p | MTMR9 |
| PELATON | hsa-miR-34a-5p | ACVR2A | WDR11-AS1 | hsa-miR-34a-5p | AAR2 |
| PELATON | hsa-miR-34a-5p | SEC13 | WDR11-AS1 | hsa-miR-34a-5p | GADD45B |
| PELATON | hsa-miR-34a-5p | IDH1 | WDR11-AS1 | hsa-miR-34a-5p | MRPS17 |
| PELATON | hsa-miR-34a-5p | HEY1 | WDR11-AS1 | hsa-miR-34a-5p | NIP7 |
| PELATON | hsa-miR-34a-5p | CARF | WDR11-AS1 | hsa-miR-34a-5p | IRAK4 |
| PELATON | hsa-miR-34a-5p | EFCAB11 | WDR11-AS1 | hsa-miR-34a-5p | SUFU |
| PELATON | hsa-miR-34a-5p | GOT2 | WDR11-AS1 | hsa-miR-34a-5p | PHF20 |
| PELATON | hsa-miR-34a-5p | HMOX2 | WDR11-AS1 | hsa-miR-34a-5p | OSER1 |
| PELATON | hsa-miR-34a-5p | ZKSCAN7 | WDR11-AS1 | hsa-miR-34a-5p | GDE1 |
| PELATON | hsa-miR-34a-5p | LOXL3 | WDR11-AS1 | hsa-miR-34a-5p | NKIRAS2 |
| PELATON | hsa-miR-34a-5p | ZNF717 | WDR11-AS1 | hsa-miR-34a-5p | THUMPD1 |
| PELATON | hsa-miR-34a-5p | ZNF304 | WDR11-AS1 | hsa-miR-34a-5p | POLR3E |
| PELATON | hsa-miR-34a-5p | STYK1 | WDR11-AS1 | hsa-miR-34a-5p | TMEM33 |
| PELATON | hsa-miR-34a-5p | UNC45A | WDR11-AS1 | hsa-miR-34a-5p | HMG20A |
| PELATON | hsa-miR-34a-5p | NIT2 | WDR11-AS1 | hsa-miR-34a-5p | ARFGAP1 |
| PELATON | hsa-miR-34a-5p | TIGAR | WDR11-AS1 | hsa-miR-34a-5p | SIRT2 |
| PELATON | hsa-miR-34a-5p | REXO4 | WDR11-AS1 | hsa-miR-34a-5p | DIDO1 |
| PELATON | hsa-miR-34a-5p | PAIP2B | WDR11-AS1 | hsa-miR-34a-5p | VCAM1 |
| PELATON | hsa-miR-34a-5p | NRIP3 | WDR11-AS1 | hsa-miR-34a-5p | HSPE1-MOB4 |
| PELATON | hsa-miR-34a-5p | TRMT5 | WDR11-AS1 | hsa-miR-34a-5p | GK |
| PELATON | hsa-miR-34a-5p | RNASEL | WDR11-AS1 | hsa-miR-34a-5p | CASP10 |
| PELATON | hsa-miR-34a-5p | TP53INP2 | WDR11-AS1 | hsa-miR-34a-5p | NFIC |
| PELATON | hsa-miR-34a-5p | PPCDC | WDR11-AS1 | hsa-miR-34a-5p | CDC6 |
| PELATON | hsa-miR-34a-5p | MRPL17 | WDR11-AS1 | hsa-miR-34a-5p | CERS6 |
| PELATON | hsa-miR-34a-5p | ITGB1BP1 | WDR11-AS1 | hsa-miR-34a-5p | TMCO1 |
| PELATON | hsa-miR-34a-5p | XRCC4 | WDR11-AS1 | hsa-miR-34a-5p | CYBRD1 |
| PELATON | hsa-miR-34a-5p | MRPL57 | WDR11-AS1 | hsa-miR-34a-5p | UCK1 |
| PELATON | hsa-miR-34a-5p | WDR77 | WDR11-AS1 | hsa-miR-34a-5p | TTPAL |
| PELATON | hsa-miR-34a-5p | THAP9 | WDR11-AS1 | hsa-miR-34a-5p | EIF6 |
| PELATON | hsa-miR-34a-5p | JHY | WDR11-AS1 | hsa-miR-34a-5p | TFAM |
| PELATON | hsa-miR-34a-5p | CPED1 | WDR11-AS1 | hsa-miR-34a-5p | TREML1 |
| PELATON | hsa-miR-34a-5p | NUP210 | WDR11-AS1 | hsa-miR-34a-5p | AAGAB |
| PELATON | hsa-miR-34a-5p | ARHGAP39 | WDR11-AS1 | hsa-miR-34a-5p | TARS2 |
| PELATON | hsa-miR-34a-5p | NELL2 | WDR11-AS1 | hsa-miR-34a-5p | STK25 |
| PELATON | hsa-miR-34a-5p | NGLY1 | WDR11-AS1 | hsa-miR-34a-5p | LPAR4 |
| PELATON | hsa-miR-34a-5p | DSN1 | WDR11-AS1 | hsa-miR-34a-5p | ACVR2A |
| PELATON | hsa-miR-34a-5p | LSS | WDR11-AS1 | hsa-miR-34a-5p | SEC13 |
| PELATON | hsa-miR-34a-5p | PLA2G4C | WDR11-AS1 | hsa-miR-34a-5p | IDH1 |
| PELATON | hsa-miR-34a-5p | NRG1 | WDR11-AS1 | hsa-miR-34a-5p | HEY1 |
| PELATON | hsa-miR-34a-5p | SMIM12 | WDR11-AS1 | hsa-miR-34a-5p | CARF |
| PELATON | hsa-miR-34a-5p | LEF1 | WDR11-AS1 | hsa-miR-34a-5p | EFCAB11 |
| PELATON | hsa-miR-34a-5p | XIAP | WDR11-AS1 | hsa-miR-34a-5p | GOT2 |
| PELATON | hsa-miR-34a-5p | ZNF548 | WDR11-AS1 | hsa-miR-34a-5p | HMOX2 |
| PELATON | hsa-miR-34a-5p | CFAP251 | WDR11-AS1 | hsa-miR-34a-5p | ZKSCAN7 |
| PELATON | hsa-miR-34a-5p | NRF1 | WDR11-AS1 | hsa-miR-34a-5p | LOXL3 |
| PELATON | hsa-miR-34a-5p | OMD | WDR11-AS1 | hsa-miR-34a-5p | ZNF717 |
| PELATON | hsa-miR-34a-5p | PDIA3 | WDR11-AS1 | hsa-miR-34a-5p | ZNF304 |
| PELATON | hsa-miR-34a-5p | PDCD7 | WDR11-AS1 | hsa-miR-34a-5p | STYK1 |
| PELATON | hsa-miR-34a-5p | TRIM13 | WDR11-AS1 | hsa-miR-34a-5p | UNC45A |
| PELATON | hsa-miR-34a-5p | RRAS | WDR11-AS1 | hsa-miR-34a-5p | NIT2 |
| PELATON | hsa-miR-34a-5p | ZNHIT1 | WDR11-AS1 | hsa-miR-34a-5p | TIGAR |
| PELATON | hsa-miR-34a-5p | PNMA2 | WDR11-AS1 | hsa-miR-34a-5p | REXO4 |
| PELATON | hsa-miR-34a-5p | GTF3C4 | WDR11-AS1 | hsa-miR-34a-5p | PAIP2B |
| PELATON | hsa-miR-34a-5p | ZNF180 | WDR11-AS1 | hsa-miR-34a-5p | NRIP3 |
| PELATON | hsa-miR-34a-5p | PRRC2B | WDR11-AS1 | hsa-miR-34a-5p | TRMT5 |
| PELATON | hsa-miR-34a-5p | ZBTB43 | WDR11-AS1 | hsa-miR-34a-5p | RNASEL |
| PELATON | hsa-miR-34a-5p | LRP10 | WDR11-AS1 | hsa-miR-34a-5p | TP53INP2 |
| PELATON | hsa-miR-34a-5p | DLST | WDR11-AS1 | hsa-miR-34a-5p | PPCDC |
| PELATON | hsa-miR-34a-5p | EZH1 | WDR11-AS1 | hsa-miR-34a-5p | MRPL17 |
| PELATON | hsa-miR-34a-5p | SLC2A5 | WDR11-AS1 | hsa-miR-34a-5p | ITGB1BP1 |
| PELATON | hsa-miR-34a-5p | SOX4 | WDR11-AS1 | hsa-miR-34a-5p | XRCC4 |
| PELATON | hsa-miR-34a-5p | UGDH | WDR11-AS1 | hsa-miR-34a-5p | MRPL57 |
| PELATON | hsa-miR-34a-5p | ZNF264 | WDR11-AS1 | hsa-miR-34a-5p | WDR77 |
| PELATON | hsa-miR-34a-5p | ZNF76 | WDR11-AS1 | hsa-miR-34a-5p | THAP9 |
| PELATON | hsa-miR-34a-5p | H2BC21 | WDR11-AS1 | hsa-miR-34a-5p | JHY |
| PELATON | hsa-miR-34a-5p | BCL10 | WDR11-AS1 | hsa-miR-34a-5p | CPED1 |
| PELATON | hsa-miR-34a-5p | ATP6V1F | WDR11-AS1 | hsa-miR-34a-5p | NUP210 |
| PELATON | hsa-miR-34a-5p | MED17 | WDR11-AS1 | hsa-miR-34a-5p | ARHGAP39 |
| PELATON | hsa-miR-34a-5p | LY86 | WDR11-AS1 | hsa-miR-34a-5p | NELL2 |
| PELATON | hsa-miR-34a-5p | EGR3 | WDR11-AS1 | hsa-miR-34a-5p | NGLY1 |
| PELATON | hsa-miR-34a-5p | PKP2 | WDR11-AS1 | hsa-miR-34a-5p | DSN1 |
| PELATON | hsa-miR-34a-5p | MAPKAPK2 | WDR11-AS1 | hsa-miR-34a-5p | LSS |
| PELATON | hsa-miR-34a-5p | RTN2 | WDR11-AS1 | hsa-miR-34a-5p | PLA2G4C |
| PELATON | hsa-miR-34a-5p | CRACDL | WDR11-AS1 | hsa-miR-34a-5p | NRG1 |
| PELATON | hsa-miR-34a-5p | AREL1 | WDR11-AS1 | hsa-miR-34a-5p | SMIM12 |
| PELATON | hsa-miR-34a-5p | PLEKHM3 | WDR11-AS1 | hsa-miR-34a-5p | LEF1 |
| PELATON | hsa-miR-34a-5p | SMIM15 | WDR11-AS1 | hsa-miR-34a-5p | XIAP |
| PELATON | hsa-miR-34a-5p | UBXN2B | WDR11-AS1 | hsa-miR-34a-5p | ZNF548 |
| PELATON | hsa-miR-34a-5p | TMEM192 | WDR11-AS1 | hsa-miR-34a-5p | CFAP251 |
| PELATON | hsa-miR-34a-5p | ATP6V0E2 | WDR11-AS1 | hsa-miR-34a-5p | NRF1 |
| PELATON | hsa-miR-34a-5p | ADH6 | WDR11-AS1 | hsa-miR-34a-5p | OMD |
| PELATON | hsa-miR-34a-5p | NCBP3 | WDR11-AS1 | hsa-miR-34a-5p | PDIA3 |
| PELATON | hsa-miR-34a-5p | DDAH1 | WDR11-AS1 | hsa-miR-34a-5p | PDCD7 |
| PELATON | hsa-miR-34a-5p | DIPK2A | WDR11-AS1 | hsa-miR-34a-5p | TRIM13 |
| PELATON | hsa-miR-34a-5p | XPR1 | WDR11-AS1 | hsa-miR-34a-5p | RRAS |
| PELATON | hsa-miR-34a-5p | ALS2 | WDR11-AS1 | hsa-miR-34a-5p | ZNHIT1 |
| PELATON | hsa-miR-34a-5p | KBTBD8 | WDR11-AS1 | hsa-miR-34a-5p | PNMA2 |
| PELATON | hsa-miR-34a-5p | RSPO3 | WDR11-AS1 | hsa-miR-34a-5p | GTF3C4 |
| PELATON | hsa-miR-34a-5p | DIXDC1 | WDR11-AS1 | hsa-miR-34a-5p | ZNF180 |
| PELATON | hsa-miR-34a-5p | PABIR1 | WDR11-AS1 | hsa-miR-34a-5p | PRRC2B |
| PELATON | hsa-miR-34a-5p | MALSU1 | WDR11-AS1 | hsa-miR-34a-5p | ZBTB43 |
| PELATON | hsa-miR-34a-5p | MPLKIP | WDR11-AS1 | hsa-miR-34a-5p | LRP10 |
| PELATON | hsa-miR-34a-5p | ZNF561 | WDR11-AS1 | hsa-miR-34a-5p | DLST |
| PELATON | hsa-miR-34a-5p | CCR1 | WDR11-AS1 | hsa-miR-34a-5p | EZH1 |
| PELATON | hsa-miR-34a-5p | LSAMP | WDR11-AS1 | hsa-miR-34a-5p | SLC2A5 |
| PELATON | hsa-miR-34a-5p | ASB8 | WDR11-AS1 | hsa-miR-34a-5p | SOX4 |
| PELATON | hsa-miR-34a-5p | MOV10 | WDR11-AS1 | hsa-miR-34a-5p | UGDH |
| PELATON | hsa-miR-34a-5p | PUS10 | WDR11-AS1 | hsa-miR-34a-5p | ZNF264 |
| PELATON | hsa-miR-34a-5p | TOR1A | WDR11-AS1 | hsa-miR-34a-5p | ZNF76 |
| PELATON | hsa-miR-34a-5p | TIMP3 | WDR11-AS1 | hsa-miR-34a-5p | H2BC21 |
| PELATON | hsa-miR-34a-5p | UMPS | WDR11-AS1 | hsa-miR-34a-5p | BCL10 |
| PELATON | hsa-miR-34a-5p | TNF | WDR11-AS1 | hsa-miR-34a-5p | ATP6V1F |
| PELATON | hsa-miR-34a-5p | PDE3A | WDR11-AS1 | hsa-miR-34a-5p | MED17 |
| PELATON | hsa-miR-34a-5p | MED11 | WDR11-AS1 | hsa-miR-34a-5p | LY86 |
| PELATON | hsa-miR-34a-5p | PM20D2 | WDR11-AS1 | hsa-miR-34a-5p | EGR3 |
| PELATON | hsa-miR-34a-5p | RTL5 | WDR11-AS1 | hsa-miR-34a-5p | PKP2 |
| PELATON | hsa-miR-34a-5p | ZNF16 | WDR11-AS1 | hsa-miR-34a-5p | MAPKAPK2 |
| PELATON | hsa-miR-34a-5p | CASTOR1 | WDR11-AS1 | hsa-miR-34a-5p | RTN2 |
| PELATON | hsa-miR-34a-5p | LINS1 | WDR11-AS1 | hsa-miR-34a-5p | CRACDL |
| PELATON | hsa-miR-449b-5p | TOR1A | WDR11-AS1 | hsa-miR-34a-5p | AREL1 |
| PELATON | hsa-miR-449b-5p | ZNF706 | WDR11-AS1 | hsa-miR-34a-5p | PLEKHM3 |
| PELATON | hsa-miR-449b-5p | MTMR9 | WDR11-AS1 | hsa-miR-34a-5p | SMIM15 |
| PELATON | hsa-miR-449b-5p | PHF20 | WDR11-AS1 | hsa-miR-34a-5p | UBXN2B |
| PELATON | hsa-miR-449b-5p | STX6 | WDR11-AS1 | hsa-miR-34a-5p | TMEM192 |
| PELATON | hsa-miR-449b-5p | UBE4A | WDR11-AS1 | hsa-miR-34a-5p | ATP6V0E2 |
| PELATON | hsa-miR-449b-5p | ST7L | WDR11-AS1 | hsa-miR-34a-5p | ADH6 |
| PELATON | hsa-miR-449b-5p | CYB561 | WDR11-AS1 | hsa-miR-34a-5p | NCBP3 |
| PELATON | hsa-miR-449b-5p | CD300A | WDR11-AS1 | hsa-miR-34a-5p | DDAH1 |
| PELATON | hsa-miR-449b-5p | RNF157 | WDR11-AS1 | hsa-miR-34a-5p | DIPK2A |
| PELATON | hsa-miR-449b-5p | DDAH1 | WDR11-AS1 | hsa-miR-34a-5p | XPR1 |
| PELATON | hsa-miR-449b-5p | R3HCC1L | WDR11-AS1 | hsa-miR-34a-5p | ALS2 |
| PELATON | hsa-miR-449b-5p | TLE4 | WDR11-AS1 | hsa-miR-34a-5p | KBTBD8 |
| PELATON | hsa-miR-449b-5p | PBX1 | WDR11-AS1 | hsa-miR-34a-5p | RSPO3 |
| PELATON | hsa-miR-449b-5p | N4BP2L1 | WDR11-AS1 | hsa-miR-34a-5p | DIXDC1 |
| PELATON | hsa-miR-449b-5p | MEA1 | WDR11-AS1 | hsa-miR-34a-5p | PABIR1 |
| PELATON | hsa-miR-449b-5p | DIPK2A | WDR11-AS1 | hsa-miR-34a-5p | MALSU1 |
| PELATON | hsa-miR-449b-5p | BCLAF3 | WDR11-AS1 | hsa-miR-34a-5p | MPLKIP |
| PELATON | hsa-miR-449b-5p | ADK | WDR11-AS1 | hsa-miR-34a-5p | ZNF561 |
| PELATON | hsa-miR-449b-5p | ZC3H12C | WDR11-AS1 | hsa-miR-34a-5p | CCR1 |
| PELATON | hsa-miR-449b-5p | TTC39B | WDR11-AS1 | hsa-miR-34a-5p | LSAMP |
| PELATON | hsa-miR-449b-5p | CASP6 | WDR11-AS1 | hsa-miR-34a-5p | ASB8 |
| PELATON | hsa-miR-449b-5p | GNPDA1 | WDR11-AS1 | hsa-miR-34a-5p | MOV10 |
| PELATON | hsa-miR-449b-5p | MIER3 | WDR11-AS1 | hsa-miR-34a-5p | PUS10 |
| PELATON | hsa-miR-449b-5p | GLE1 | WDR11-AS1 | hsa-miR-34a-5p | TOR1A |
| PELATON | hsa-miR-449b-5p | SH3GLB2 | WDR11-AS1 | hsa-miR-34a-5p | TIMP3 |
| PELATON | hsa-miR-449b-5p | ALG2 | WDR11-AS1 | hsa-miR-34a-5p | UMPS |
| PELATON | hsa-miR-449b-5p | NABP2 | WDR11-AS1 | hsa-miR-34a-5p | TNF |
| PELATON | hsa-miR-449b-5p | LLGL2 | WDR11-AS1 | hsa-miR-34a-5p | PDE3A |
| PELATON | hsa-miR-449b-5p | ADGRE2 | WDR11-AS1 | hsa-miR-34a-5p | MED11 |
| PELATON | hsa-miR-449b-5p | OSER1 | WDR11-AS1 | hsa-miR-34a-5p | PM20D2 |
| PELATON | hsa-miR-449b-5p | FAHD2A | WDR11-AS1 | hsa-miR-34a-5p | RTL5 |
| PELATON | hsa-miR-449b-5p | TRMT61B | WDR11-AS1 | hsa-miR-34a-5p | ZNF16 |
| PELATON | hsa-miR-449b-5p | FAM161B | WDR11-AS1 | hsa-miR-34a-5p | CASTOR1 |
| PELATON | hsa-miR-449b-5p | CFAP57 | WDR11-AS1 | hsa-miR-34a-5p | LINS1 |
| PELATON | hsa-miR-449b-5p | ELAPOR2 | WDR11-AS1 | hsa-miR-20b-5p | ZNF264 |
| PELATON | hsa-miR-449b-5p | ST6GALNAC3 | WDR11-AS1 | hsa-miR-20b-5p | CASP6 |
| PELATON | hsa-miR-449b-5p | CADM2 | WDR11-AS1 | hsa-miR-20b-5p | PLPP5 |
| PELATON | hsa-miR-449b-5p | CD200R1 | WDR11-AS1 | hsa-miR-20b-5p | MOSMO |
| PELATON | hsa-miR-449b-5p | KCNQ4 | WDR11-AS1 | hsa-miR-20b-5p | SLC25A45 |
| PELATON | hsa-miR-449b-5p | UNC45B | WDR11-AS1 | hsa-miR-20b-5p | PTGR2 |
| PELATON | hsa-miR-449b-5p | RTTN | WDR11-AS1 | hsa-miR-20b-5p | GALNT15 |
| PELATON | hsa-miR-449b-5p | RIMKLA | WDR11-AS1 | hsa-miR-20b-5p | BTRC |
| PELATON | hsa-miR-449b-5p | PPARD | WDR11-AS1 | hsa-miR-20b-5p | FOLH1 |
| PELATON | hsa-miR-449b-5p | CFAP418 | WDR11-AS1 | hsa-miR-20b-5p | RNF157 |
| PELATON | hsa-miR-449b-5p | BTN2A2 | WDR11-AS1 | hsa-miR-20b-5p | CPT2 |
| PELATON | hsa-miR-449b-5p | TPRG1L | WDR11-AS1 | hsa-miR-20b-5p | NSUN7 |
| PELATON | hsa-miR-449b-5p | LANCL3 | WDR11-AS1 | hsa-miR-20b-5p | RHBDD2 |
| PELATON | hsa-miR-449b-5p | PLEKHG7 | WDR11-AS1 | hsa-miR-20b-5p | ODF2 |
| PELATON | hsa-miR-449b-5p | SPDYE15 | WDR11-AS1 | hsa-miR-20b-5p | MFSD8 |
| PELATON | hsa-miR-449b-5p | PRDM11 | WDR11-AS1 | hsa-miR-20b-5p | UCK2 |
| PELATON | hsa-miR-449b-5p | TDRP | WDR11-AS1 | hsa-miR-20b-5p | TSEN15 |
| PELATON | hsa-miR-449b-5p | PPFIA4 | WDR11-AS1 | hsa-miR-20b-5p | MRTFB |
| PELATON | hsa-miR-449b-5p | SMIM20 | WDR11-AS1 | hsa-miR-20b-5p | ADGRL2 |
| PELATON | hsa-miR-449b-5p | EIF3I | WDR11-AS1 | hsa-miR-20b-5p | FCRLA |
| PELATON | hsa-miR-449b-5p | ZBTB34 | WDR11-AS1 | hsa-miR-20b-5p | RNF212 |
| PELATON | hsa-miR-449b-5p | MTCH2 | WDR11-AS1 | hsa-miR-20b-5p | FOXO3B |
| PELATON | hsa-miR-449b-5p | RNF144A | WDR11-AS1 | hsa-miR-20b-5p | ADK |
| PELATON | hsa-miR-449b-5p | LCMT2 | WDR11-AS1 | hsa-miR-20b-5p | MON2 |
| PELATON | hsa-miR-449b-5p | EPM2AIP1 | WDR11-AS1 | hsa-miR-20b-5p | MIER3 |
| PELATON | hsa-miR-449b-5p | SUPT7L | WDR11-AS1 | hsa-miR-20b-5p | SIPA1L2 |
| PELATON | hsa-miR-449b-5p | RNF44 | WDR11-AS1 | hsa-miR-20b-5p | CDH5 |
| PELATON | hsa-miR-449b-5p | ADNP2 | WDR11-AS1 | hsa-miR-20b-5p | GORASP1 |
| PELATON | hsa-miR-449b-5p | ANKS1A | WDR11-AS1 | hsa-miR-20b-5p | ZFP3 |
| PELATON | hsa-miR-449b-5p | KHNYN | WDR11-AS1 | hsa-miR-20b-5p | SLC9A9 |
| PELATON | hsa-miR-449b-5p | NCAPH | WDR11-AS1 | hsa-miR-20b-5p | ZNF620 |
| PELATON | hsa-miR-449b-5p | CNRIP1 | WDR11-AS1 | hsa-miR-20b-5p | CBFA2T3 |
| PELATON | hsa-miR-449b-5p | TMEM251 | WDR11-AS1 | hsa-miR-20b-5p | NR2C2AP |
| PELATON | hsa-miR-449b-5p | IRAK4 | WDR11-AS1 | hsa-miR-20b-5p | SESTD1 |
| PELATON | hsa-miR-449b-5p | PALS2 | WDR11-AS1 | hsa-miR-20b-5p | GEN1 |
| PELATON | hsa-miR-449b-5p | PSMC3IP | WDR11-AS1 | hsa-miR-20b-5p | RNF144B |
| PELATON | hsa-miR-449b-5p | PAXBP1 | WDR11-AS1 | hsa-miR-20b-5p | KIF9 |
| PELATON | hsa-miR-449b-5p | RWDD2B | WDR11-AS1 | hsa-miR-20b-5p | PRNP |
| PELATON | hsa-miR-449b-5p | TERF1 | WDR11-AS1 | hsa-miR-20b-5p | CPM |
| PELATON | hsa-miR-449b-5p | THUMPD1 | WDR11-AS1 | hsa-miR-20b-5p | JHY |
| PELATON | hsa-miR-449b-5p | LRRC8D | WDR11-AS1 | hsa-miR-20b-5p | CENPS |
| PELATON | hsa-miR-449b-5p | DIDO1 | WDR11-AS1 | hsa-miR-20b-5p | POLQ |
| PELATON | hsa-miR-449b-5p | KAT7 | WDR11-AS1 | hsa-miR-20b-5p | DSTYK |
| PELATON | hsa-miR-449b-5p | VCAM1 | WDR11-AS1 | hsa-miR-20b-5p | MOB3C |
| PELATON | hsa-miR-449b-5p | TNFSF8 | WDR11-AS1 | hsa-miR-20b-5p | GSTCD |
| PELATON | hsa-miR-449b-5p | ARNTL2 | WDR11-AS1 | hsa-miR-20b-5p | IL17REL |
| PELATON | hsa-miR-449b-5p | CYBRD1 | WDR11-AS1 | hsa-miR-20b-5p | HDAC5 |
| PELATON | hsa-miR-449b-5p | URM1 | WDR11-AS1 | hsa-miR-20b-5p | PRDM11 |
| PELATON | hsa-miR-449b-5p | TFAM | WDR11-AS1 | hsa-miR-20b-5p | ADPRH |
| PELATON | hsa-miR-449b-5p | PUS7L | WDR11-AS1 | hsa-miR-20b-5p | RUBCN |
| PELATON | hsa-miR-449b-5p | STK25 | WDR11-AS1 | hsa-miR-20b-5p | RNF44 |
| PELATON | hsa-miR-449b-5p | ACVR2A | WDR11-AS1 | hsa-miR-20b-5p | MESD |
| PELATON | hsa-miR-449b-5p | SMAP1 | WDR11-AS1 | hsa-miR-20b-5p | FBXO28 |
| PELATON | hsa-miR-449b-5p | TRIB1 | WDR11-AS1 | hsa-miR-20b-5p | PARM1 |
| PELATON | hsa-miR-449b-5p | HECTD2 | WDR11-AS1 | hsa-miR-20b-5p | HEATR5A |
| PELATON | hsa-miR-449b-5p | UTP15 | WDR11-AS1 | hsa-miR-20b-5p | EDRF1 |
| PELATON | hsa-miR-449b-5p | LOXL3 | WDR11-AS1 | hsa-miR-20b-5p | IRAK4 |
| PELATON | hsa-miR-449b-5p | MYADM | WDR11-AS1 | hsa-miR-20b-5p | DCTN4 |
| PELATON | hsa-miR-449b-5p | CTNNBIP1 | WDR11-AS1 | hsa-miR-20b-5p | HACD3 |
| PELATON | hsa-miR-449b-5p | ZBTB21 | WDR11-AS1 | hsa-miR-20b-5p | THUMPD1 |
| PELATON | hsa-miR-449b-5p | SIPA1L2 | WDR11-AS1 | hsa-miR-20b-5p | RMDN3 |
| PELATON | hsa-miR-449b-5p | USP37 | WDR11-AS1 | hsa-miR-20b-5p | RNF145 |
| PELATON | hsa-miR-449b-5p | BCL7A | WDR11-AS1 | hsa-miR-20b-5p | PTPRN |
| PELATON | hsa-miR-449b-5p | MTARC1 | WDR11-AS1 | hsa-miR-20b-5p | VCAM1 |
| PELATON | hsa-miR-449b-5p | NAA35 | WDR11-AS1 | hsa-miR-20b-5p | EGR3 |
| PELATON | hsa-miR-449b-5p | THAP9 | WDR11-AS1 | hsa-miR-20b-5p | GK |
| PELATON | hsa-miR-449b-5p | ASB13 | WDR11-AS1 | hsa-miR-20b-5p | IQSEC2 |
| PELATON | hsa-miR-449b-5p | JHY | WDR11-AS1 | hsa-miR-20b-5p | ARNTL2 |
| PELATON | hsa-miR-449b-5p | PHAF1 | WDR11-AS1 | hsa-miR-20b-5p | CERS6 |
| PELATON | hsa-miR-449b-5p | B4GALT2 | WDR11-AS1 | hsa-miR-20b-5p | IL12RB2 |
| PELATON | hsa-miR-449b-5p | DUSP16 | WDR11-AS1 | hsa-miR-20b-5p | PSMD9 |
| PELATON | hsa-miR-449b-5p | BHLHE41 | WDR11-AS1 | hsa-miR-20b-5p | TTPAL |
| PELATON | hsa-miR-449b-5p | DSN1 | WDR11-AS1 | hsa-miR-20b-5p | URM1 |
| PELATON | hsa-miR-449b-5p | SEMA7A | WDR11-AS1 | hsa-miR-20b-5p | WTAP |
| PELATON | hsa-miR-449b-5p | PTGR2 | WDR11-AS1 | hsa-miR-20b-5p | HYDIN |
| PELATON | hsa-miR-449b-5p | ZNF260 | WDR11-AS1 | hsa-miR-20b-5p | C11orf71 |
| PELATON | hsa-miR-449b-5p | LEF1 | WDR11-AS1 | hsa-miR-20b-5p | CARHSP1 |
| PELATON | hsa-miR-449b-5p | PGAM5 | WDR11-AS1 | hsa-miR-20b-5p | FBXO31 |
| PELATON | hsa-miR-449b-5p | SH2B3 | WDR11-AS1 | hsa-miR-20b-5p | CARF |
| PELATON | hsa-miR-449b-5p | MTF1 | WDR11-AS1 | hsa-miR-20b-5p | TRIB1 |
| PELATON | hsa-miR-449b-5p | RRAS | WDR11-AS1 | hsa-miR-20b-5p | SH3GLB2 |
| PELATON | hsa-miR-449b-5p | TUBGCP3 | WDR11-AS1 | hsa-miR-20b-5p | TBCE |
| PELATON | hsa-miR-449b-5p | TRIM27 | WDR11-AS1 | hsa-miR-20b-5p | USP49 |
| PELATON | hsa-miR-449b-5p | POLD3 | WDR11-AS1 | hsa-miR-20b-5p | RBBP6 |
| PELATON | hsa-miR-449b-5p | PHTF1 | WDR11-AS1 | hsa-miR-20b-5p | GNG12 |
| PELATON | hsa-miR-449b-5p | LYVE1 | WDR11-AS1 | hsa-miR-20b-5p | KLHL7 |
| PELATON | hsa-miR-449b-5p | ZNF16 | WDR11-AS1 | hsa-miR-20b-5p | TMCO1 |
| PELATON | hsa-miR-449b-5p | ZNF19 | WDR11-AS1 | hsa-miR-20b-5p | TIGAR |
| PELATON | hsa-miR-449b-5p | INTS6 | WDR11-AS1 | hsa-miR-20b-5p | NRIP3 |
| PELATON | hsa-miR-449b-5p | PHLDA3 | WDR11-AS1 | hsa-miR-20b-5p | TSPYL4 |
| PELATON | hsa-miR-449b-5p | OSTM1 | WDR11-AS1 | hsa-miR-20b-5p | DIDO1 |
| PELATON | hsa-miR-449b-5p | GCLC | WDR11-AS1 | hsa-miR-20b-5p | RBSN |
| PELATON | hsa-miR-449b-5p | HSBP1 | WDR11-AS1 | hsa-miR-20b-5p | MTARC1 |
| PELATON | hsa-miR-449b-5p | ATP2B4 | WDR11-AS1 | hsa-miR-20b-5p | USP46 |
| PELATON | hsa-miR-449b-5p | CDR2 | WDR11-AS1 | hsa-miR-20b-5p | UBE2Z |
| PELATON | hsa-miR-449b-5p | DLST | WDR11-AS1 | hsa-miR-20b-5p | TMEM106C |
| PELATON | hsa-miR-449b-5p | GFPT1 | WDR11-AS1 | hsa-miR-20b-5p | SAP30L |
| PELATON | hsa-miR-449b-5p | LAMC1 | WDR11-AS1 | hsa-miR-20b-5p | TRMT1L |
| PELATON | hsa-miR-449b-5p | P2RX1 | WDR11-AS1 | hsa-miR-20b-5p | NECAP2 |
| PELATON | hsa-miR-449b-5p | SDC1 | WDR11-AS1 | hsa-miR-20b-5p | ZNF737 |
| PELATON | hsa-miR-449b-5p | COX15 | WDR11-AS1 | hsa-miR-20b-5p | NRG1 |
| PELATON | hsa-miR-449b-5p | LIN7A | WDR11-AS1 | hsa-miR-20b-5p | PCYT1B |
| PELATON | hsa-miR-449b-5p | CRACDL | WDR11-AS1 | hsa-miR-20b-5p | ZFHX3 |
| PELATON | hsa-miR-449b-5p | SIGLEC15 | WDR11-AS1 | hsa-miR-20b-5p | ZBED1 |
| PELATON | hsa-miR-449b-5p | TSPAN4 | WDR11-AS1 | hsa-miR-20b-5p | TK2 |
| PELATON | hsa-miR-449b-5p | PEX7 | WDR11-AS1 | hsa-miR-20b-5p | ZNF347 |
| PELATON | hsa-miR-449b-5p | DELE1 | WDR11-AS1 | hsa-miR-20b-5p | KCNK6 |
| PELATON | hsa-miR-449b-5p | FMNL2 | WDR11-AS1 | hsa-miR-20b-5p | IL27RA |
| PELATON | hsa-miR-449b-5p | GZF1 | WDR11-AS1 | hsa-miR-20b-5p | FEZ2 |
| PELATON | hsa-miR-449b-5p | HYAL1 | WDR11-AS1 | hsa-miR-20b-5p | FEZ1 |
| PELATON | hsa-miR-449b-5p | SMIM15 | WDR11-AS1 | hsa-miR-20b-5p | ZMPSTE24 |
| PELATON | hsa-miR-449b-5p | BEX4 | WDR11-AS1 | hsa-miR-20b-5p | SIGMAR1 |
| PELATON | hsa-miR-449b-5p | TMEM192 | WDR11-AS1 | hsa-miR-20b-5p | MTF1 |
| PELATON | hsa-miR-449b-5p | NCBP3 | WDR11-AS1 | hsa-miR-20b-5p | TSPAN31 |
| PELATON | hsa-miR-449b-5p | SH2D1A | WDR11-AS1 | hsa-miR-20b-5p | LIPG |
| PELATON | hsa-miR-449b-5p | GEN1 | WDR11-AS1 | hsa-miR-20b-5p | VIL1 |
| PELATON | hsa-miR-449b-5p | CRYZ | WDR11-AS1 | hsa-miR-20b-5p | ZXDA |
| PELATON | hsa-miR-449b-5p | PDLIM4 | WDR11-AS1 | hsa-miR-20b-5p | INTS6 |
| PELATON | hsa-miR-449b-5p | CISD3 | WDR11-AS1 | hsa-miR-20b-5p | GTF3C4 |
| PELATON | hsa-miR-449b-5p | PHAX | WDR11-AS1 | hsa-miR-20b-5p | OSTM1 |
| PELATON | hsa-miR-449b-5p | RSPO3 | WDR11-AS1 | hsa-miR-20b-5p | SPCS1 |
| PELATON | hsa-miR-449b-5p | NFATC2IP | WDR11-AS1 | hsa-miR-20b-5p | GLE1 |
| PELATON | hsa-miR-449b-5p | CASP2 | WDR11-AS1 | hsa-miR-20b-5p | CD69 |
| PELATON | hsa-miR-449b-5p | PURB | WDR11-AS1 | hsa-miR-20b-5p | DUSP7 |
| PELATON | hsa-miR-449b-5p | ZFHX2 | WDR11-AS1 | hsa-miR-20b-5p | E2F4 |
| PELATON | hsa-miR-449b-5p | DIXDC1 | WDR11-AS1 | hsa-miR-20b-5p | RGS16 |
| PELATON | hsa-miR-449b-5p | CIPC | WDR11-AS1 | hsa-miR-20b-5p | TCF3 |
| PELATON | hsa-miR-449b-5p | ELMO1 | WDR11-AS1 | hsa-miR-20b-5p | LIN7A |
| PELATON | hsa-miR-449b-5p | ZNF483 | WDR11-AS1 | hsa-miR-20b-5p | VNN1 |
| PELATON | hsa-miR-449b-5p | MALSU1 | WDR11-AS1 | hsa-miR-20b-5p | NDUFAF7 |
| PELATON | hsa-miR-449b-5p | GTPBP8 | WDR11-AS1 | hsa-miR-20b-5p | GPRASP1 |
| PELATON | hsa-miR-449b-5p | EFCAB11 | WDR11-AS1 | hsa-miR-20b-5p | TMEM192 |
| PELATON | hsa-miR-449b-5p | CCDC127 | WDR11-AS1 | hsa-miR-20b-5p | CD55 |
| PELATON | hsa-miR-449b-5p | ZNF561 | WDR11-AS1 | hsa-miR-20b-5p | MET |
| PELATON | hsa-miR-449b-5p | PRH1-TAS2R14 | WDR11-AS1 | hsa-miR-20b-5p | TRMT10A |
| PELATON | hsa-miR-449b-5p | CINP | WDR11-AS1 | hsa-miR-20b-5p | ZMAT4 |
| PELATON | hsa-miR-449b-5p | NRG1 | WDR11-AS1 | hsa-miR-20b-5p | ZBTB43 |
| PELATON | hsa-miR-449b-5p | UMPS | WDR11-AS1 | hsa-miR-20b-5p | C19orf12 |
| PELATON | hsa-miR-449b-5p | PDE3A | WDR11-AS1 | hsa-miR-20b-5p | RAMAC |
| PELATON | hsa-miR-449b-5p | HMGXB4 | WDR11-AS1 | hsa-miR-20b-5p | FSD1L |
| PELATON | hsa-miR-449b-5p | C2orf68 | WDR11-AS1 | hsa-miR-20b-5p | TM2D2 |
| PELATON | hsa-miR-449b-5p | C17orf97 | WDR11-AS1 | hsa-miR-20b-5p | KBTBD8 |
| PELATON | hsa-miR-449b-5p | RTL5 | WDR11-AS1 | hsa-miR-20b-5p | EAF1 |
| PELATON | hsa-miR-449b-5p | FOXK1 | WDR11-AS1 | hsa-miR-20b-5p | PURB |
| PELATON | hsa-miR-449b-5p | HEY1 | WDR11-AS1 | hsa-miR-20b-5p | CDC14A |
| PELATON | hsa-miR-449c-5p | INTS6 | WDR11-AS1 | hsa-miR-20b-5p | DIXDC1 |
| PELATON | hsa-miR-449c-5p | MESD | WDR11-AS1 | hsa-miR-20b-5p | CIPC |
| PELATON | hsa-miR-449c-5p | THAP1 | WDR11-AS1 | hsa-miR-20b-5p | MPLKIP |
| PELATON | hsa-miR-449c-5p | CNIH4 | WDR11-AS1 | hsa-miR-20b-5p | ST7L |
| PELATON | hsa-miR-449c-5p | RUBCN | WDR11-AS1 | hsa-miR-20b-5p | CMBL |
| PELATON | hsa-miR-449c-5p | SCIN | WDR11-AS1 | hsa-miR-20b-5p | C15orf40 |
| PELATON | hsa-miR-449c-5p | HFE | WDR11-AS1 | hsa-miR-20b-5p | GABPB2 |
| PELATON | hsa-miR-449c-5p | MEA1 | WDR11-AS1 | hsa-miR-20b-5p | TRAF5 |
| PELATON | hsa-miR-449c-5p | CD300A | WDR11-AS1 | hsa-miR-20b-5p | CLEC12A |
| PELATON | hsa-miR-449c-5p | RHBDD2 | WDR11-AS1 | hsa-miR-20b-5p | TNF |
| PELATON | hsa-miR-449c-5p | C16orf87 | WDR11-AS1 | hsa-miR-20b-5p | GOLGA8B |
| PELATON | hsa-miR-449c-5p | PBX1 | WDR11-AS1 | hsa-miR-20b-5p | RNLS |
| PELATON | hsa-miR-449c-5p | SPTY2D1OS | WDR11-AS1 | hsa-miR-20b-5p | CBFA2T2 |
| PELATON | hsa-miR-449c-5p | POLD3 |  |  |  |

**Supplementary Table 7.** PPI Analysis.

| #node1 | node2 | #node1 | node2 | #node1 | node2 |
| --- | --- | --- | --- | --- | --- |
| ABCG1 | LIPG | CHD8 | PHAX | LIPG | TNF |
| ABCG1 | PPARD | CHD8 | ZBTB7A | LIPG | PLPP5 |
| ABCG1 | JAM2 | CHD8 | NFATC2IP | LRP10 | SDC1 |
| ABCG1 | TNF | CHD8 | RPRD2 | LRRC41 | RNF7 |
| ABCG2 | LYVE1 | CHD8 | PHF20 | LRRC8D | OMD |
| ABCG2 | MET | CHD8 | E2F6 | LRRIQ3 | R3HCC1L |
| ABCG2 | GCLC | CHD8 | HMG20A | LSAMP | SLC1A3 |
| ABO | FOXK1 | CHD8 | MBD5 | LSAMP | PRNP |
| ABRAXAS2 | LAMC1 | CHD8 | PPP2R5D | LSM4 | WDR77 |
| ABRAXAS2 | SH2B3 | CINP | POLR2F | LSM4 | WTAP |
| ACBD6 | ACOX1 | CINP | CLUAP1 | LSM4 | SRPK1 |
| ACBD6 | NTPCR | CIPC | ITGB1BP1 | LSM4 | UPF1 |
| ACOX1 | PEX16 | CLEC12A | STX6 | LSS | PNKD |
| ACOX1 | PPARGC1A | CLEC12A | XCR1 | LSS | SIGMAR1 |
| ACOX1 | PECR | CLUAP1 | NMNAT1 | LYVE1 | TNF |
| ACOX1 | TYSND1 | CLUAP1 | MAK16 | MAK16 | PNO1 |
| ACOX1 | ADH5 | CMBL | TK2 | MAK16 | UTP15 |
| ACOX1 | SLC25A20 | CMC2 | TP53I11 | MAK16 | PUS7L |
| ACOX1 | PPARD | CMTR1 | NCAPH | MAK16 | NOLC1 |
| ACOX1 | DHRS4 | CMTR1 | UPF1 | MAK16 | NSUN7 |
| ACOX1 | IDH1 | CMTR1 | ZNF76 | MAK16 | RIOK1 |
| ACOX1 | ALDH3A2 | COP1 | TRPC4AP | MALSU1 | MRPL17 |
| ACOX1 | CPT2 | COP1 | SEM1 | MALSU1 | MRPL57 |
| ACP5 | OSTM1 | COP1 | MYADM | MALSU1 | PUS7L |
| ACP5 | MITF | COP1 | TRIB3 | MALSU1 | NMNAT1 |
| ACP5 | EIF3I | CPT2 | PPARGC1A | MALSU1 | POLR2F |
| ACP5 | PHEX | CPT2 | PPARD | MALSU1 | MRPL42 |
| ACP5 | TNF | CPT2 | SLC25A20 | MAP1A | PPARGC1A |
| ACVR2A | TGFBR3 | CPT2 | TFAM | MAP1A | RUBCN |
| ADAM17 | TIMP3 | CPT2 | IDH1 | MARCHF3 | STX6 |
| ADAM17 | NRG1 | CRYZ | GCLC | MCM4 | NCAPH |
| ADAM17 | DDIT4 | CYBRD1 | SLC39A1 | MCM4 | POLD3 |
| ADAM17 | SDC1 | CYBRD1 | TFR2 | MEA1 | PPP2R5D |
| ADAM17 | TNF | CYBRD1 | HFE | MED11 | MED17 |
| ADGRL2 | BAALC | DAAM2 | DVL3 | MED11 | PPARGC1A |
| ADGRL2 | LRCH2 | DAAM2 | PRICKLE2 | MED11 | POLR2F |
| ADGRL2 | LRRC8D | DAAM2 | RYK | MED11 | MED19 |
| ADGRL2 | LRRIQ3 | DBN1 | SCIN | MED17 | PPARGC1A |
| ADGRL2 | OMD | DCTN1 | SIGMAR1 | MED17 | POLR2F |
| ADH5 | PECR | DCTN1 | GORASP1 | MED17 | MED19 |
| ADH5 | GSTM4 | DCTN1 | TNF | MED19 | PPARGC1A |
| ADH5 | ALDH3A2 | DCTN1 | PRICKLE2 | MED19 | POLR2F |
| ADH6 | PECR | DCTN1 | KIF14 | MET | RRAS |
| ADH6 | ALDH3A2 | DCTN1 | SHC1 | MET | NRG1 |
| ADH6 | GSTM4 | DCTN1 | ODF2 | MET | SDC1 |
| ADK | UMPS | DDIT4 | TRIB3 | MET | NF2 |
| ADK | TK2 | DEDD | PARN | MET | PTPRB |
| ADK | PDE3A | DELE1 | THAP1 | MET | MITF |
| ADK | NTPCR | DELE1 | TOR1A | MET | SLC9A9 |
| ADPRH | NECAP2 | DELE1 | RNF145 | MET | TNF |
| ADPRH | OARD1 | DHRS4 | TYSND1 | MET | SHC1 |
| AGO3 | ZNF264 | DLST | IDH1 | MFF | PEX16 |
| AGO3 | TERF1 | DPY19L3 | ZFHX3 | MFF | RMDN3 |
| AGO3 | ASH2L | DPY19L3 | RSPO3 | MFF | PPARGC1A |
| AGO3 | POP5 | DPYS | UMPS | MFF | PPARD |
| AGO3 | H2BC21 | DSTYK | NECAP2 | MFF | TFAM |
| ALDH3A2 | GDAP1 | DSTYK | USP49 | MFF | PGAM5 |
| ALDH3A2 | PEX16 | DUSP7 | KLHL18 | MICU2 | RMDN3 |
| ALDH3A2 | PECR | DUSP7 | SPOP | MICU2 | SIGMAR1 |
| ALDH3A2 | DLST | DVL3 | SEM1 | MICU2 | SMAP1 |
| ALDH3A2 | UCK2 | DVL3 | RYK | MIEN1 | POLR3H |
| ALDH3A2 | UBE2J2 | DVL3 | PPP2R5D | MITF | PAIP2B |
| ALS2 | SIGMAR1 | DVL3 | FZD5 | MLX | PPCDC |
| ALS2 | DCTN1 | DVL3 | PRICKLE2 | MOB3C | NAA20 |
| AP4E1 | RTN2 | E2F4 | E2F6 | MOSMO | RNF157 |
| AP4E1 | HEATR5A | E2F6 | PHF20 | MRPL17 | POLR2F |
| AP4E1 | C19orf12 | E2F6 | EZH1 | MRPL17 | PUS7L |
| AP4E1 | LMBRD2 | EEF1AKMT2 | NDUFAF7 | MRPL17 | MRPL57 |
| APPL2 | POLR3H | EEF1AKMT2 | EEF2KMT | MRPL17 | MRPL42 |
| ARFGAP1 | NECAP2 | EEF2KMT | NR2C2AP | MRPL42 | MRPL57 |
| ARFGAP1 | SMAP1 | EFNA4 | MET | MSR1 | RNASEL |
| ARFGAP1 | KIF9 | EGR3 | NRG1 | MSR1 | TNF |
| ARFGAP1 | DCTN1 | EIF3I | PNO1 | MTCH2 | SLC25A28 |
| ARHGAP39 | DBN1 | EIF3I | UPF1 | MTX3 | SLC39A1 |
| ARHGAP39 | ZNF623 | EIF3I | RTTN | NAA20 | NAA35 |
| ASB13 | RNF7 | EIF3I | EIF6 | NAA35 | UTP15 |
| ASB13 | UBE4A | EIF6 | IMP4 | NAA35 | TSTD2 |
| ASB8 | RNF7 | EIF6 | KCNQ4 | NAP1L5 | TSPYL4 |
| ASCC1 | MRPL17 | EIF6 | MAK16 | NCAPH | SRPK1 |
| ASCC1 | XRCC4 | EIF6 | MALSU1 | NCBP3 | PHAX |
| ASH2L | MED17 | EIF6 | NSUN7 | NECAP2 | UBE2J2 |
| ASH2L | ZNHIT1 | ELMO1 | ELMO2 | NF2 | NRG1 |
| ASH2L | TM2D2 | ELMO1 | KNDC1 | NF2 | NFIC |
| ASH2L | PLPP5 | ELMO2 | FEZ1 | NF2 | SDC1 |
| ASH2L | TLE4 | ELMO2 | INPP5K | NF2 | SUFU |
| ASH2L | BTRC | EPG5 | MON2 | NFATC2IP | ZNHIT1 |
| ASH2L | POLR2F | ERAL1 | TFAM | NFIC | SLC1A3 |
| ASH2L | E2F6 | ERAL1 | MALSU1 | NFIC | POLR3H |
| ASH2L | PHF20 | ERAL1 | GTPBP8 | NFIC | POLR2F |
| ASH2L | EZH1 | EZH1 | ZNF76 | NFIC | POLR3G |
| ASH2L | H2BC21 | FBH1 | POLQ | NFIC | ZFHX3 |
| ASH2L | CHD8 | FBH1 | NFATC2IP | NMNAT1 | SIRT2 |
| ASH2L | SDC1 | FBH1 | GEN1 | NOLC1 | UMPS |
| ATF7 | CHD8 | FCRL5 | SIGLEC10 | NOLC1 | UTP15 |
| B4GALT2 | OMD | FCRL5 | SDC1 | NOLC1 | PHAX |
| BATF | CD69 | FCRL5 | FCRLA | NRF1 | PPARGC1A |
| BATF | SOX4 | FEZ1 | SAP30L | NRF1 | PPARD |
| BATF | TCF3 | FEZ1 | UNC119 | NRF1 | ZNF212 |
| BATF | BIRC5 | FOXK1 | ING2 | NRF1 | TFAM |
| BATF | SH2D1A | FOXK1 | MBD5 | NRG1 | SHC1 |
| BATF | TOX2 | FZD5 | STX6 | NSUN7 | TRMT10A |
| BATF | TNF | FZD5 | RYK | NSUN7 | UGDH |
| BCL10 | DDAH1 | GADD45B | MCM4 | NSUN7 | PUS7L |
| BCL10 | CASP10 | GADD45B | TNF | P2RY13 | SELPLG |
| BCL10 | BTRC | GCLC | UGDH | PAIP2B | PRNP |
| BCL10 | XIAP | GCLC | GSTM4 | PARN | TERF1 |
| BCL10 | TNF | GCLC | TNF | PARN | PHAX |
| BCL7A | WDR66 | GDAP1 | PLEKHM3 | PARN | UPF1 |
| BCL7A | RPRD2 | GDAP1 | MFF | PBX1 | TCF3 |
| BCLAF3 | MED17 | GDAP1 | PEX16 | PDIA3 | PRNP |
| BCLAF3 | MED11 | GDPD4 | RAB30 | PECR | PNKD |
| BCLAF3 | MED19 | GEN1 | HUS1 | PECR | TYSND1 |
| BHLHE41 | RIOK1 | GEN1 | POLQ | PGAM5 | WDR77 |
| BHLHE41 | BTRC | GEN1 | SEM1 | PGAM5 | XIAP |
| BIRC5 | CDC6 | GFPT1 | UGDH | PHF20 | TRPC4AP |
| BIRC5 | NCAPH | GFPT1 | GNPDA1 | PLEKHG7 | ZNF483 |
| BIRC5 | MED17 | GLI3 | SUFU | PLEKHG7 | RIMS2 |
| BIRC5 | MCM4 | GLI3 | SPOP | PLPP5 | TM2D2 |
| BIRC5 | DDIT4 | GLI3 | SEM1 | PNKD | THAP1 |
| BIRC5 | GCLC | GNPDA1 | LAMC1 | PNKD | TOR1A |
| BIRC5 | CASP2 | GOLPH3L | RWDD2B | PNO1 | RIOK1 |
| BIRC5 | DSN1 | GORASP1 | GORASP2 | PNO1 | UTP15 |
| BIRC5 | KIF14 | GORASP1 | JAM2 | POLD3 | SEM1 |
| BIRC5 | XIAP | GORASP1 | STK25 | POLD3 | TERF1 |
| BLMH | PDIA3 | GORASP2 | STX6 | POLQ | XRCC4 |
| BLMH | HFE | GORASP2 | KIF14 | POLR2F | UMPS |
| BTRC | TUBGCP3 | GORASP2 | JAM2 | POLR2F | POLR3H |
| BTRC | RNF7 | GPC6 | LRP10 | POLR2F | WTAP |
| BTRC | DDIT4 | GPC6 | SDC1 | POLR2F | RPRD2 |
| BTRC | SUFU | GPN3 | POLR3H | POLR2F | POLR3G |
| BTRC | ZBTB46 | GPN3 | POLR2F | POLR3G | POLR3H |
| BTRC | SEM1 | GSTM4 | MYADM | POLR3H | TRMT10A |
| BTRC | SPOP | GTF3C4 | KAT7 | PPARD | PPARGC1A |
| BTRC | TNF | GTF3C4 | MCM4 | PPARD | TNF |
| BTRC | TLE4 | GTF3C4 | POLR3H | PPARD | TFAM |
| BTRC | PPP2R5D | GTF3C4 | POLR2F | PPARGC1A | SIRT2 |
| BTRC | GLI3 | GTF3C4 | POLR3G | PPARGC1A | SUFU |
| C15orf40 | KLHDC10 | GTPBP8 | MRPL17 | PPARGC1A | SLC25A20 |
| C16orf87 | MIER3 | GTPBP8 | PUS7L | PPARGC1A | TNF |
| C19orf12 | RTN2 | GTPBP8 | MALSU1 | PPARGC1A | TFAM |
| CASK | SDC1 | GZMK | TNF | PPFIA4 | RIMS2 |
| CASK | POLR2F | GZMK | SH2D1A | PRDM10 | RRAS |
| CASK | PPFIA4 | H2BC21 | HDAC5 | PRDM10 | SPOP |
| CASK | LIN7A | H2BC21 | SIRT2 | PRICKLE2 | RYK |
| CASP10 | UPF1 | H2BC21 | KAT7 | PRNP | TNF |
| CASP10 | DEDD | H2BC21 | TERF1 | PRNP | TRIB3 |
| CASP10 | TNF | H2BC21 | KAT6B | PTPRN | SH2B3 |
| CASP10 | CASP2 | H2BC21 | SAP30L | PTPRN | TOR1A |
| CASP10 | XIAP | H2BC21 | UNC119 | PUS10 | TRMT5 |
| CASP2 | TNF | H2BC21 | PSMC3IP | PUS10 | PUS7L |
| CASP2 | XIAP | H2BC21 | TADA2A | PVRIG | SPCS1 |
| CASTOR1 | DEPDC5 | H2BC21 | USP46 | PVRIG | SH2D1A |
| CBFA2T3 | TCF3 | H2BC21 | TNF | RAB3D | STX6 |
| CBLB | MET | H2BC21 | POLR2F | RAB3D | RIMS2 |
| CBLB | SHC1 | H2BC21 | USP49 | RBBP6 | WDR77 |
| CCDC115 | TMEM87A | HDAC5 | KAT7 | RBSN | STX6 |
| CCDC115 | CMTR1 | HDAC5 | SIRT2 | RHBDD2 | ZBTB34 |
| CCR5 | TNFSF8 | HFE | TERF1 | RIOK1 | WDR77 |
| CCR5 | CD69 | HFE | SLC39A1 | RMDN3 | SIGMAR1 |
| CCR5 | SELPLG | HFE | TFR2 | RNASEL | ZFHX3 |
| CCR5 | GZMK | HMG20A | PPCDC | RNASEL | TNF |
| CCR5 | MSR1 | HMGXB4 | HMOX2 | RNASEL | SPOP |
| CCR5 | P2RY13 | HUS1 | XRCC4 | RNF157 | UNC45B |
| CCR5 | XCR1 | HUS1 | SEM1 | RPRD2 | TARS2 |
| CCR5 | CD1D | HYDIN | KIF9 | RSPO3 | TGFBR3 |
| CCR5 | TNF | IDH1 | TIGAR | RTN2 | TMEM33 |
| CD1D | CD22 | IDH1 | SIRT2 | SDC1 | SOX4 |
| CD1D | CD69 | IDH1 | PPARGC1A | SDC1 | TNF |
| CD1D | SIGLEC10 | IDH1 | TYSND1 | SELPLG | TNF |
| CD1D | SH2D1A | IDH1 | SH2B3 | SEM1 | SUFU |
| CD1D | SDC1 | IDH1 | SPOP | SEM1 | SPOP |
| CD1D | TNF | IDH1 | TNF | SEM1 | TNF |
| CD1D | CDH5 | IL27RA | TNF | SESTD1 | TBCE |
| CD200R1 | CD22 | IMP4 | NSUN7 | SH2B3 | TOR1A |
| CD200R1 | MSR1 | IMP4 | NOLC1 | SH2D1A | XIAP |
| CD200R1 | SHC1 | IMP4 | PUS7L | SHC1 | TNF |
| CD200R1 | TNF | IMP4 | MRPL17 | SIGLEC10 | SIGLEC15 |
| CD200R1 | CD300A | IMP4 | MAK16 | SIGMAR1 | TMEM97 |
| CD22 | CLEC12A | IMP4 | PNO1 | SIKE1 | STK25 |
| CD22 | SIGLEC10 | IMP4 | UTP15 | SIRT2 | SPOP |
| CD22 | FCRL5 | ING2 | KAT7 | SLC1A3 | SLC3A1 |
| CD22 | TNF | ING2 | KAT6B | SMIM12 | UTP15 |
| CD22 | SDC1 | ING2 | SAP30L | SMIM12 | TCAIM |
| CD22 | FCRLA | INPP5K | SCIN | SPOP | SUFU |
| CD22 | CD69 | INPP5K | PLCH1 | STK25 | TNF |
| CD22 | SIGLEC15 | INPP5K | ZFHX2 | TADA2A | USP49 |
| CD300A | TREML1 | ITGB1BP1 | STK25 | TFAM | TK2 |
| CD69 | SELPLG | KAT6B | KAT7 | TFAM | TNF |
| CD69 | SH2D1A | KAT7 | SIRT2 | TGFBR3 | TNF |
| CD69 | SDC1 | KBTBD8 | KLHL7 | THAP1 | THAP9 |
| CD69 | GZMK | KBTBD8 | SPOP | THAP1 | TOR1A |
| CD69 | TNF | KBTBD8 | NOLC1 | TIMP3 | TNF |
| CDC6 | SEM1 | KHNYN | RNASEL | TK2 | UMPS |
| CDC6 | TERF1 | KIF14 | RAB3D | TK2 | UCK2 |
| CDC6 | E2F6 | KIF14 | NCAPH | TK2 | UCK1 |
| CDC6 | DDIT4 | KIF14 | MCM4 | TLE4 | XIAP |
| CDC6 | GEN1 | KIF14 | POLQ | TMEM192 | TUBGCP3 |
| CDC6 | KIF14 | KIF14 | KIF9 | TNF | TNFSF8 |
| CDC6 | NCAPH | KIF14 | MTX3 | TNF | TRAF5 |
| CDC6 | KAT7 | KIF9 | KLHL18 | TNF | XIAP |
| CDC6 | E2F4 | KLHL7 | SPOP | TNFSF8 | TRAF5 |
| CDC6 | MCM4 | KNDC1 | ZSCAN20 | TRAF5 | XIAP |
| CDH5 | LYVE1 | LAMC1 | LYVE1 | TRMT10A | TRMT5 |
| CDH5 | PTPRB | LAMC1 | MET | TSPAN31 | TSPAN4 |
| CDH5 | ITGB1BP1 | LAMC1 | TIMP3 | UBE4A | UNC45B |
| CDH5 | JAM2 | LAMC1 | PRNP | UBE4A | UNC45A |
| CDH5 | TNF | LCMT2 | TRMT5 | UCK1 | UMPS |
| CDH5 | SHC1 | LCMT2 | PNO1 | UCK1 | UCK2 |
| CDH5 | LIN7A | LIN7A | STYK1 | UCK2 | UMPS |
| CEP162 | RTTN | LIN7A | SDC1 | USP46 | WDR77 |
| CERS6 | MFF | LIN7A | PPFIA4 | XCR1 | ZBTB46 |
| CFAP57 | HYDIN | LIPG | TTC39B |  |  |
